# The Complexity of Dynamics in Small Neural Circuits

**DOI:** 10.1371/journal.pcbi.1004992

**Published:** 2016-08-05

**Authors:** Diego Fasoli, Anna Cattani, Stefano Panzeri

**Affiliations:** Laboratory of Neural Computation, Center for Neuroscience and Cognitive Systems @UniTn, Istituto Italiano di Tecnologia, Rovereto, Italy; University of Pittsburgh, UNITED STATES

## Abstract

Mean-field approximations are a powerful tool for studying large neural networks. However, they do not describe well the behavior of networks composed of a small number of neurons. In this case, major differences between the mean-field approximation and the real behavior of the network can arise. Yet, many interesting problems in neuroscience involve the study of mesoscopic networks composed of a few tens of neurons. Nonetheless, mathematical methods that correctly describe networks of small size are still rare, and this prevents us to make progress in understanding neural dynamics at these intermediate scales. Here we develop a novel systematic analysis of the dynamics of arbitrarily small networks composed of homogeneous populations of excitatory and inhibitory firing-rate neurons. We study the local bifurcations of their neural activity with an approach that is largely analytically tractable, and we numerically determine the global bifurcations. We find that for strong inhibition these networks give rise to very complex dynamics, caused by the formation of multiple branching solutions of the neural dynamics equations that emerge through spontaneous symmetry-breaking. This qualitative change of the neural dynamics is a finite-size effect of the network, that reveals qualitative and previously unexplored differences between mesoscopic cortical circuits and their mean-field approximation. The most important consequence of spontaneous symmetry-breaking is the ability of mesoscopic networks to regulate their degree of functional heterogeneity, which is thought to help reducing the detrimental effect of noise correlations on cortical information processing.

## Author Summary

The mesoscopic level of brain organization, describing the organization and dynamics of small circuits of neurons including from few tens to few thousands, has recently received considerable experimental attention. It is useful for describing small neural systems of invertebrates, and in mammalian neural systems it is often seen as a middle ground that is fundamental to link single neuron activity to complex functions and behavior. However, and somewhat counter-intuitively, the behavior of neural networks of small and intermediate size can be much more difficult to study mathematically than that of large networks, and appropriate mathematical methods to study the dynamics of such networks have not been developed yet. Here we consider a model of a network of firing-rate neurons with arbitrary finite size, and we study its local bifurcations using an analytical approach. This analysis, complemented by numerical studies for both the local and global bifurcations, shows the emergence of strong and previously unexplored finite-size effects that are particularly hard to detect in large networks. This study advances the tools available for the comprehension of finite-size neural circuits, going beyond the insights provided by the mean-field approximation and the current techniques for the quantification of finite-size effects.

## Introduction

The brain is a complex system organized at multiple spatial scales, and the concerted interactions between these multiple scales of organization are probably crucial for the emergence of its computational power [24]. At the coarsest level, the brain can be divided into macroscopic circuits spanning several areas and containing millions of neurons. These macroscopic circuits are believed to accomplish very complex functions, ranging from motor control to cognition. At the finest level of organization, basic computations are performed at the single cell level by neurons, which can be seen as elementary computational units [20]. However, there is an intermediate, mesoscopic level of organization between the macroscopic and microscopic one: neurons are organized in microcircuits, whose size can vary from several thousands of cells as in cortical columns, to a few tens of cells as in micro-columns [45–47]. This mesoscopic level of investigation has received considerable attention in recent years both from the theoretical [25–29] and experimental [21, 22] point of view, and it is often seen as a middle ground that is fundamental to link single neuron activity to behavior [23]. For this reason, finding an appropriate mathematical description of the brain at the mesoscopic scale is of fundamental importance for unveiling its emergent properties.

At the mesoscopic scale, the brain is often described as a collection of neural masses, i.e. homogeneous neuronal populations within a cortical column [30]. Usually, these groups of neurons are described by the so-called *neural-mass models* [31]. A typical example is the well-known *Jansen-Rit model* [32–34], which describes local cortical circuits as a population of excitatory pyramidal cells, which receive inhibitory and excitatory feedback from local interneurons, and excitatory input from other regions such as the thalamus. This class of models may be studied using mean-field theory [35]. Mean-field theory is a mathematical tool that approximates the behavior of large networks [36–38], and it is useful to study the mass activity of few thousands of neurons, which constitutes the upper limit of mesoscopic descriptions of neural circuits and can adapt well to describe for example measures based on LFP/MEG/EEG recordings. This approximation becomes exact in the limit of networks with an infinite number of neurons, the so-called *thermodynamic limit*. For finite-size networks, however, the mean-field theory provides only an approximation of the real behavior of the system, and therefore may neglect important phenomena, such as qualitative differences in the transitions between static regimes and chaos [39], or in the degree or nature of correlations among neurons [40]. Clearly, these macroscopic differences in the dynamical and statistical behavior of finite and infinite-size networks may have important consequences on the information processing capability of the system, as potential oversimplifications in the mean-field approximation may hide important neural processes that are fundamental for the comprehension of neural circuits of finite size.

To go beyond the mean field limit, several mathematical techniques have been developed to quantify finite-size effects. These finite-size methods (such as the linear noise approximation [41], the density functional approach [42], large-deviations theory [43] and path-integral methods [44]) typically can only be applied to mesoscopic circuits composed of a finite but large number of neurons.

On the other side, most of the literature about small neural circuits deals with networks composed of 2–4 neurons (e.g. [1–3]), while methods for the analysis of networks made of a few tens of neurons, which represent the lower bound of the mesoscopic scale, are still limited. The purpose of our work is to make progress in the mathematical methodology to study the dynamics of such networks. The analysis of the dynamics of small neural networks was pioneered by Beer, who studied the bifurcations of networks of arbitrary size with highly symmetric assumptions on the strength of the synaptic weights [48], and through asymptotic approximations of the bifurcation manifolds [4]. In our article we extend his analysis to a more biologically plausible network of arbitrary size by deriving exact expressions of the bifurcation manifolds, and with less rigid constraints on the synaptic weights. In more detail, we consider a deterministic finite-size firing-rate network model with excitatory and inhibitory populations composed of an arbitrary number of homogeneous neurons in each population. Moreover, the model is characterized by homogeneous and arbitrarily strong weights between the populations. Then, we perform a numerical analysis of the global bifurcations that emerge by varying the external input currents (i.e. the stimuli) to the network and the strength of inhibition, and we introduce a mathematical theory that describes local bifurcations analytically. We find qualitative differences with the mean-field approximation when the system has strong inhibitory synaptic weights. In this case, through a phenomenon of spontaneous symmetry-breaking, the neural network undergoes a special bifurcation known as *pitchfork* or *branching point* [49], from which multiple solutions of the neural equations emerge. On the new branches, new bifurcations can occur, enriching considerably the complexity of the bifurcation diagram of the neural network. This dynamics is not revealed by the mean-field approximation.

This article is organized as follows. In Materials and Methods we describe the neural model we use. We then explain intuitively in Results the formation of new branches of solutions through spontaneous symmetry-breaking, depending on the strength of inhibition. This is followed by a numerical and analytical study of the bifurcations of the network in weak and strong-inhibition regimes for different sizes of the inhibitory population. In Discussion we conclude by examining the biological implications of our results and by comparing our approach with previous work on homogeneous networks.

## Materials and Methods

**Fig 1:**
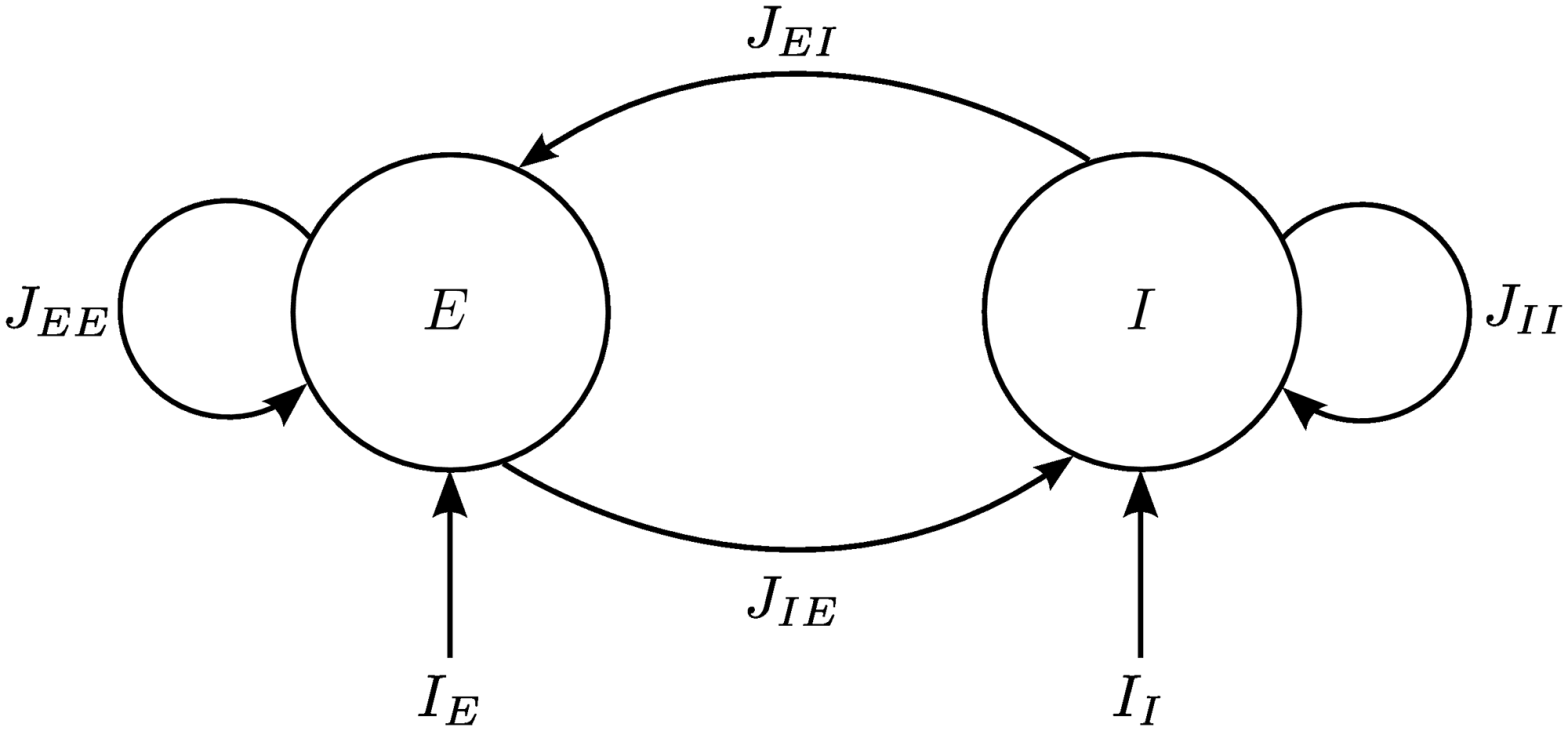
Structure of the analyzed network. Neurons are fully connected and grouped into one excitatory and one inhibitory population. *J*_*αβ*_ represents the set of synaptic weights from the population *β* to the population *α*, while *I*_*α*_ is the external current (i.e. the stimulus) to the population *α*.

Here we describe the assumptions we made about the model of finite-size neural circuits, whose structure is schematized in Fig 1, and whose dynamics we would like to investigate mathematically. These assumptions represent the best compromise we could find between biological detail, biological plausibility and mathematical tractability. We perform a numerical and analytical study considering the case of two neural populations of excitatory and inhibitory neurons respectively. The populations contain an arbitrary finite number of neurons, which are connected with each other through non-symmetric synaptic connections with arbitrarily strong weights. In order to make the network analytically tractable, we make some simplifying assumptions. In particular, we assume (as it is often made when considering local cortical circuits [30, 31]) that the neurons in each population have homogeneous properties and parameters, that all neurons in the network are connected to each other (fully-connected topology), and that the axonal delays are negligible. Moreover, we study the deterministic neural equations, as it is common practice in the analysis of bifurcations. Therefore we do not consider typical sources of randomness such as stochastic fluctuations in the firing rates and random distributions of the strength of the synaptic weights. However, we note that some of these assumptions can be overcome by extensions of this formalism (see Discussion).

In more detail, we consider a widely used rate model to describe the dynamics of single neurons [30, 35, 38, 40, 48, 53]:

$$\begin{array}{c} \frac{dV_{i}\left(t\right)}{dt}=-\frac{1}{\tau_{i}}V_{i}\left(t\right)+\frac{1}{M_{i}}\sum_{j=0}^{N-1}J_{ij}A_{j}\left(V_{j}\left(t\right)\right)+I_{i},\;i=0,...,N-1, \end{array}$$ (1)

where *N* ≥ 4 represents the number of neurons in the network. The function *V*_*i*_(*t*) is the membrane potential of the *i*th neuron, while *τ*_*i*_ is its membrane time constant. *M*_*i*_, the total number of connections to the *i*th neuron, is a normalization factor that has been introduced to avoid the explosion of the total synaptic input $\sum_{j=0}^{N-1}J_{ij}A_{j}(V_{j}(t))$ in Eq (1) in the thermodynamic limit *N* → ∞. The synaptic connectivity matrix *J* specifies the strength of the synaptic connections in the network. *J*_*ij*_ is the synaptic weight from the *j*th to the *i*th neuron, and for simplicity it is assumed to be deterministic and constant in time, for every pair of neurons. $A_{j}$ (·) is the activation function of the *j*th neuron and converts its membrane potential into the corresponding firing rate $\nu_{j}=A(V_{j})$. Typically, piecewise-linear activation functions are required in order to obtain analytical results [11, 55, 56]. However, here we will show how to obtain explicit expressions for the equilibrium points and the local bifurcations of the network when A(V) is a smooth (i.e. infinitely differentiable) function. In more detail, we will consider *S*-shaped (i.e. sigmoidal) activation functions, since they are biologically realistic [6, 54]. Particularly convenient from the mathematical point of view is the so-called *algebraic activation function*, which is defined as follows:

$$\begin{array}{c} A_{j}\left(V\right)=\frac{\nu_{j}^{\text{max}}}{2}\left[1+\frac{\frac{\Lambda_{j}}{2}\left(V-V_{j}^{T}\right)}{\sqrt{1+\frac{\Lambda_{j}^{2}}{4}{\left(V-V_{j}^{T}\right)}^{2}}}\right]. \end{array}$$ (2)

Here *ν*^max^ is the maximum firing rate, Λ determines the slope of the activation function when *ν*^max^ is fixed, and *V*^*T*^ is the horizontal shift, therefore it can be interpreted as a firing threshold for the membrane potentials. In Eq (1), *I*_*i*_ are external input currents, namely the stimuli to the network, which here are supposed to be constant in time, in order to perform the bifurcation analysis of the network with *I*_*i*_ as bifurcation parameters.

In order to make our analysis analytically tractable, we suppose that all the parameters of the system are homogeneous for each population of neurons considered. Thus, these parameters will be indexed only at the population level. By defining *N*_*E*_ (*N*_*I*_) to be the size of the excitatory (inhibitory) population (with *N*_*E*,*I*_ ≥ 2), and by indexing the neurons of the excitatory population as *i* = 0, …, *N*_*E*_ − 1 and the inhibitory neurons as *i* = *N*_*E*_, …, *N* − 1 (with *N* = *N*_*E*_ + *N*_*I*_), the synaptic connectivity matrix is structured as follows:

$$\begin{array}{ccc} J=\left[\begin{array}{cc} J_{EE} & J_{EI}\\ J_{IE} & J_{II} \end{array}\right], & J_{\alpha \beta }=\left\{ \begin{array}{l} J_{\alpha \alpha }\left(I_{N_{\alpha }}-{\text{Id}}_{N_{\alpha }}\right),\\ J_{\alpha \beta }I_{N_{\alpha },N_{\beta }}, \end{array}\right. & \begin{array}{l} \text{ for }\alpha =\beta \\ \text{ for }\alpha \neq \beta \end{array} \end{array}$$

where the block $J_{\alpha \beta }$ is a *N*_*α*_ × *N*_*β*_ matrix that represents the connections from the population *β* to the population *α*, with *α*, *β* ∈ {*E*, *I*}. Moreover, $I_{N_{\alpha },N_{\beta }}$ is the *N*_*α*_ × *N*_*β*_ all-ones matrix (here we use the simplified notation $I_{N_{\alpha }}\overset{\text{def}}{=}I_{N_{\alpha },N_{\alpha }}$), while Id_*N*_*α*__ is the *N*_*α*_ × *N*_*α*_ identity matrix. Since approximately 80% of neocortical neurons are excitatory and the remaining 20% are inhibitory [58], we set $\frac{N_{E}}{N_{I}}=4$. This is a typical choice in many theoretical works (e.g. [9, 10]). Then we set the diagonals of $J_{EE}$ and $J_{II}$ to zero because of the absence of self-connections in biological networks. *J*_*EE*_, *J*_*II*_, *J*_*EI*_, *J*_*IE*_ are free parameters that describe the strength of the interactions between and within the neural populations. Clearly we have *J*_*EE*_, *J*_*IE*_ > 0, and *J*_*II*_,*J*_*EI*_ < 0, which also means that *M*_*E*_ = *M*_*I*_ = *N* − 1. It is important to observe that, compared to [21] where the authors divided the synaptic weights by the population size, we have chosen a different normalization. In both cases, the total synaptic input $\sum_{j=0}^{N-1}J_{ij}A_{j}(V_{j}(t))$ is convergent in the thermodynamic limit, but the two normalizations behave in different ways when the network is mixed with both finite-size and infinite-size populations. According to our normalization, if one population has infinite size and the other has a finite size, then the contribution of the finite-size population to the total synaptic input of a neuron is negligible compared to the one of the infinite-size population. The normalization introduced by [38] leads instead to the infinite-size and finite-size populations both providing finite contributions to the total synaptic input. However, if required, it is possible to switch from our normalization to the other one by performing the following substitution:

$$\begin{array}{c} J_{\alpha \beta }\to \frac{N-1}{N_{\beta }}J_{\alpha \beta } \end{array}$$

in all the analytical results reported in Results and in S1 Text.

Given the notations described above, the external input currents are defined by two vectors, ***I***_*E*_ and ***I***_*I*_, representing the inputs to the excitatory and inhibitory populations:

$$\begin{array}{c} I_{\alpha }=I_{\alpha }1_{N_{\alpha }} \end{array}$$

where $1_{N_{\alpha }}\overset{\text{def}}{=}I_{N_{\alpha },1}$ is the *N*_*α*_ × 1 all-ones vector. The same subdivision between excitatory and inhibitory populations is performed for the other parameters of the network, namely *τ*, *ν*^max^, Λ, *V*_*T*_.

To summarize, the equations of our neural system (1) can be written explicitly as follows:

$$\{\begin{array}{c} \begin{array}{lll} E:\;\frac{dV_{i}(t)}{dt} & = & -\frac{1}{\tau_{E}}V_{i}(t)\;+\;\frac{J_{EE}}{N-1}\sum_{\begin{array}{l} j=0\\ j\neq i \end{array}}^{N_{E}-1}A_{E}(V_{j}(t))\;+\;\frac{J_{EI}}{N-1}\sum_{j=N_{E}}^{N-1}A_{I}(V_{j}(t))\;+\;I_{E}\\ & & i=0,\;\ldots ,N_{E}-1, \end{array}\\ \begin{array}{lll} I:\;\frac{dV_{i}(t)}{dt} & = & -\frac{1}{\tau_{I}}V_{i}(t)\;+\;\frac{J_{IE}}{N-1}\sum_{j=0}^{N_{E}-1}A_{E}(V_{j}(t))\;+\;\frac{J_{II}}{N-1}\sum_{\begin{array}{l} j=N_{E}\\ j\neq i \end{array}}^{N-1}A_{I}(V_{j}(t))\;+\;I_{I},\\ & & i=N_{E},\;\ldots ,N-1. \end{array} \end{array}$$ (3)

## Results

Here we study the dynamics of small networks with an arbitrary number of firing rate neurons. The system is composed of two fully-connected and homogeneous neural populations of excitatory and inhibitory neurons respectively, with negligible axonal delays and without random noise. We perform a bifurcation analysis of their dynamics to understand the mechanisms underlying the emergence of complex neural activity and how they relate to biological functions.

The bifurcation analysis characterizes the dynamics the model is able to exhibit, depending on two parameters: the static external currents *I*_*E*,*I*_ in Eq (3). In particular, we perform this analysis for increasing values of a third parameter: the self-inhibition strength *J*_*II*_. We focus on this parameter, instead of the other synaptic weights (i.e. *J*_*EE*_, *J*_*EI*_, *J*_*IE*_), since *J*_*II*_ is the one responsible for the formation of the branching-point bifurcations. For the remaining synaptic weights we will provide only a qualitative description of the effects they exert on the system. The non-varying network parameters for the bifurcation analysis are set as in Table 1. In particular, we consider a network made of *N*_*E*_ = 8 excitatory and *N*_*I*_ = 2 inhibitory neurons, since we want to study finite-size effects in small neural masses.

**Table 1:** Values of the parameters used in this article.

Population Sizes	Synaptic Weights	Activation Functions	Memb. Time Consts.
*N*_*E*_ = 8	*J*_*EE*_ = 10	$\nu_{E}^{\text{max}}=\nu_{I}^{\text{max}}=1$	*τ*_*E*_ = *τ*_*I*_ = 1
*N*_*I*_ = 2	*J*_*EI*_ = −70	Λ_*E*_ = Λ_*I*_ = 2	
	*J*_*IE*_ = 70	$V_{E}^{T}=V_{I}^{T}=2$	

*J*_*II*_ and *I*_*E*,*I*_ are not fixed, since they represent the bifurcation parameters of the network.

We perform a detailed bifurcation analysis by means of numerical tools and, whenever possible, through analytical techniques. The numerical analysis is performed with the Cl_MatCont Matlab toolbox [59] and XPPAUT [60], which are built up the mathematical theory of bifurcations described in [49, 61], while the analytical results are based on linear algebra. In principle, a study of bifurcations accomplished by means of such continuation toolboxes can be difficult due to the high symmetry of Eq (3). The eigenvalues of the Jacobian matrix of highly symmetric systems can have multiplicity larger than one, leading standard numerical toolboxes to fail in detecting all bifurcations [78]. Nevertheless, by assuming *N*_*I*_ = 2 throughout the article, the eigenvalues that generate the bifurcation points have always multiplicity one, thus ensuring a correct functioning of the continuation toolboxes. Furthermore, the choice *N*_*I*_ = 2 allows us to provide a thorough bifurcation study that would have become prohibitive, in terms of clarity, for *N*_*I*_ > 2. We also implement the analytical formulas in a standalone Python script (see S1 File), which performs the analysis of local bifurcations in a few seconds without a need of further more complex tools. This script is based on the bifurcation analytical formulas presented hereafter, which assume a sigmoidal activation function as in Eq (2). We however verified that different sigmoidal functions, such as the logistic function, provide qualitatively similar bifurcation diagrams [54].

Finally, it is important to underline that bifurcations are defined by many conditions. Nonetheless, in our analytical study we checked only the conditions on the eigenvalues of the network, since they proved sufficient to reproduce the numerical results. Due to the high variety of the bifurcations the system exhibits, a full check of all the remaining (non-degeneracy) conditions is beyond the purpose of this article.

### Intuitive interpretation of the branching points

In mathematics, the branching-point bifurcations are described by the so-called *equivariant bifurcation theory* [50], namely the study of bifurcations in symmetric systems. The latter being rather technical, here we prefer to follow a more intuitive approach to the problem. First of all, we observe that according to bifurcation theory, local bifurcations are calculated by means of the eigenvalues of the Jacobian matrix of the network evaluated at the equilibrium points. Therefore, we set $\frac{dV_{i}(t)}{dt}=0\;\forall i$ in Eq (3) to compute the stationary solutions of the system. For the subsequent analysis, it is useful to introduce the following parameter:

$$\begin{array}{c} \psi \overset{\text{def}}{=}\frac{\tau_{I}\left|J_{II}\right|\nu_{I}^{\text{max}}\Lambda_{I}}{4\left(N-1\right)} \end{array}$$ (4)

and we will show that, as long as inhibition is weak (*ψ* < 1), the equilibrium points in each population are always homogeneous:

$$\begin{array}{c} \mu =\left(\overset{N_{E}-\text{times}}{\overset{︷}{\mu_{E},\ldots ,\mu_{E}}},\overset{N_{I}-\text{times}}{\overset{︷}{\mu_{I},\ldots ,\mu_{I}}}\right). \end{array}$$ (5)

Specifically, *μ*_*E*_ and *μ*_*I*_ are the solutions of the following system of algebraic non-linear equations, obtained from Eq (3):

$$\begin{array}{c} \left\{\begin{array}{c} F\left(\mu_{E},\mu_{I}\right)\overset{\text{def}}{=}-\frac{1}{\tau_{E}}\mu_{E}+\frac{N_{E}-1}{N-1}J_{EE}A_{E}\left(\mu_{E}\right)+\frac{N_{I}}{N-1}J_{EI}A_{I}\left(\mu_{I}\right)+I_{E}=0\\ G\left(\mu_{E},\mu_{I}\right)\overset{\text{def}}{=}-\frac{1}{\tau_{I}}\mu_{I}+\frac{N_{E}}{N-1}J_{IE}A_{E}\left(\mu_{E}\right)+\frac{N_{I}-1}{N-1}J_{II}A_{I}\left(\mu_{I}\right)+I_{I}=0\;. \end{array}\right. \end{array}$$ (6)

**Fig 2:**
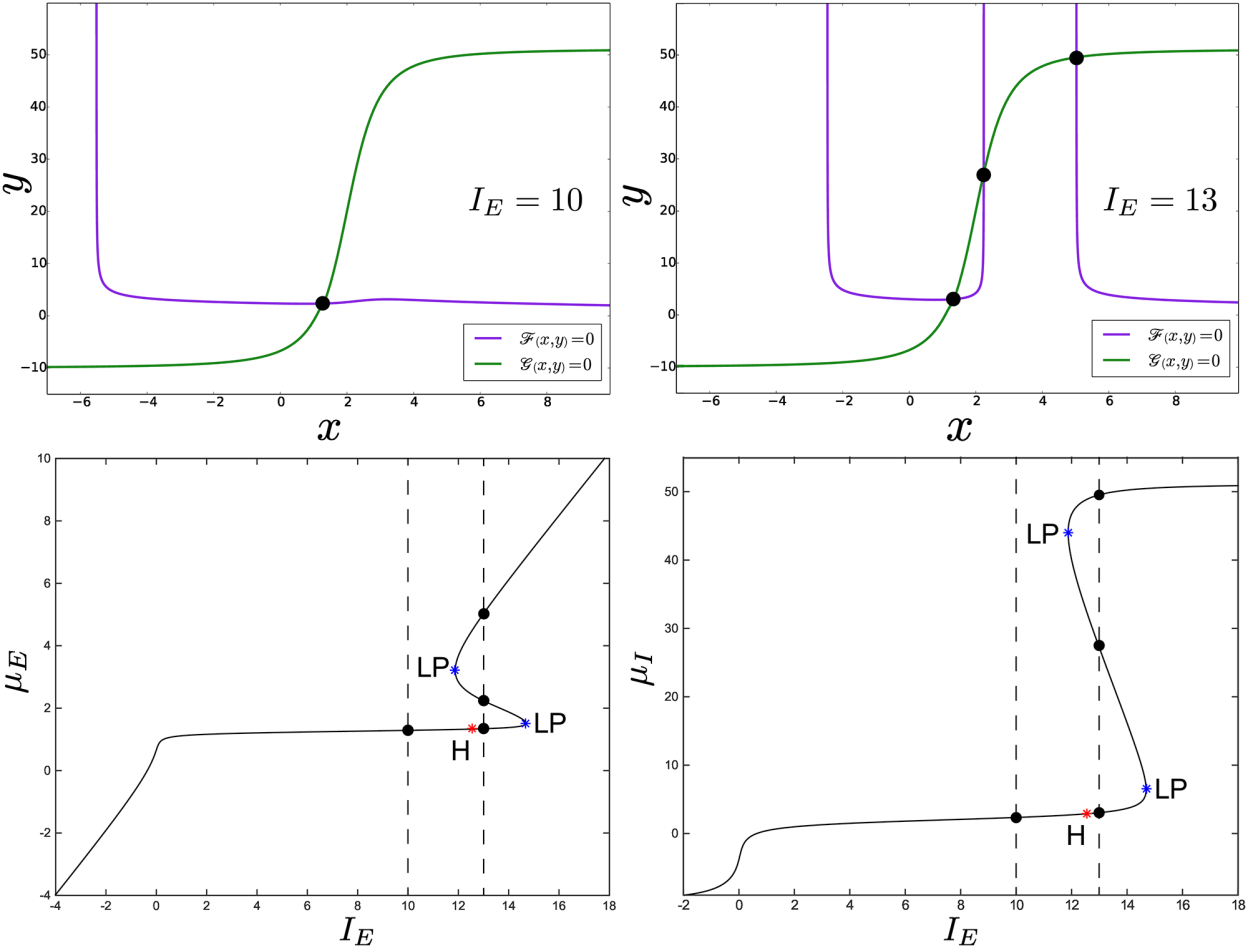
Formation of multiple stationary solutions in the weak-inhibition regime. The two top panels show the nullclines of the network for fixed values of *I*_*E*,*I*_ in a weak-inhibition regime, obtained for *J*_*II*_ = −10 and the values of the parameters in Table 1. Their intersection points (black dots) correspond to the solutions of the system (6). The top-left panel was obtained for *I*_*E*_ = 10, *I*_*I*_ = −10, while the top-right panel for *I*_*E*_ = 13, *I*_*I*_ = −10. The system (6) admits multiple solutions for specific values of the parameters. The two figures at the bottom show the solutions *μ*_*E*_ and *μ*_*I*_ (bottom-left and bottom-right panel, respectively) of the system (6) for the same values of the parameters, but with varying current *I*_*E*_. The black curve represents the primary branch of the network equations, and for *I*_*E*_ = 10 and *I*_*E*_ = 13 it admits one and three solutions respectively (see the black dots at the intersection with the vertical dashed lines). The (*μ*_*E*_,*μ*_*I*_) coordinates of these solutions correspond to those of the black dots in the top panels of the figure. The stability of these solutions is examined for the sake of completeness in S5 Fig.

The curves defined by Eqs. $F(x,y)=0\text{ and }G\left(x,y\right)=0\;\forall (x,y)\in {\mathbb{R}}^{2}$ are the so-called *nullclines* of the network [17, 18]. Fig 2 (top) shows an example obtained for *J*_*II*_ = −10, while the remaining parameters are chosen as in Table 1. For completeness, in S1 Text we show how to get approximate analytical solutions for *μ*_*E*,*I*_, even though our bifurcation analysis can be performed without knowing them explicitly.

From Eqs (3) + (5), we obtain that the Jacobian matrix J of the linearized system evaluated at the stationary solution on the primary branch Eq (5) is:

$$\begin{array}{l} J=\left[\begin{array}{cc} j_{EE} & j_{EI}\\ j_{IE} & j_{II} \end{array}\right],\\ j_{\alpha \beta }=\{\begin{array}{ll} -\frac{1}{\tau_{\alpha }}{\text{Id}}_{N_{\alpha }}+\frac{J_{\alpha \alpha }}{N-1}A_{\alpha }^{\prime }(\mu_{\alpha })(I_{N_{\alpha }}-{\text{Id}}_{N_{\alpha }}), & \text{for }\alpha =\beta \\ \frac{J_{\alpha \beta }}{N-1}A_{\beta }^{\prime }(\mu_{\beta })I_{N_{\alpha },N_{\beta }}, & \text{for }\alpha \neq \beta \end{array} \end{array}$$ (7)

From this we can prove (see S1 Text) that the eigenvalues of J are:

$$\begin{array}{cc} \lambda_{0,1}^{R} & =\frac{Y+Z\pm \sqrt{{\left(Y-Z\right)}^{2}+4x}}{2},\\ \lambda_{E} & =-\left[\frac{1}{\tau_{E}}+\frac{J_{EE}}{N-1}A_{E}^{\prime }\left(\mu_{E}\right)\right],\\ \lambda_{I} & =-\left[\frac{1}{\tau_{I}}+\frac{J_{II}}{N-1}A_{I}^{\prime }\left(\mu_{I}\right)\right], \end{array}$$ (8)

where:

$$\begin{array}{c} x=\frac{N_{E}N_{1}}{{\left(N-1\right)}^{2}}J_{EI}J_{IE}A_{E}^{\prime }\left(\mu_{E}\right)A_{I}^{\prime }\left(\mu_{I}\right),\\ Y=-\frac{1}{\tau_{E}}+\frac{N_{E}-1}{N-1}J_{EE}A_{E}^{\prime }\left(\mu_{E}\right),\\ Z=-\frac{1}{\tau_{I}}+\frac{N_{I}-1}{N-1}J_{II}A_{I}^{\prime }\left(\mu_{I}\right). \end{array}$$ (9)

The inequalities *ψ* < 1 and *λ*_*I*_ ≥ 0 imply:

$$\begin{array}{c} A_{I}^{\prime }\left(\mu_{I}\right)\geq \frac{N-1}{\tau_{I}\left|J_{II}\right|}>\frac{\nu_{I}^{\text{max}}\Lambda_{I}}{4} \end{array}$$

while, according to Eq (2), the derivative of the activation function cannot exceed $A_{I}^{\prime }(V_{I}^{T})=\frac{\nu_{I}^{\text{max}}\Lambda_{I}}{4}$. Therefore *λ*_*I*_ is always negative in the weak-inhibition regime.

**Fig 3:**
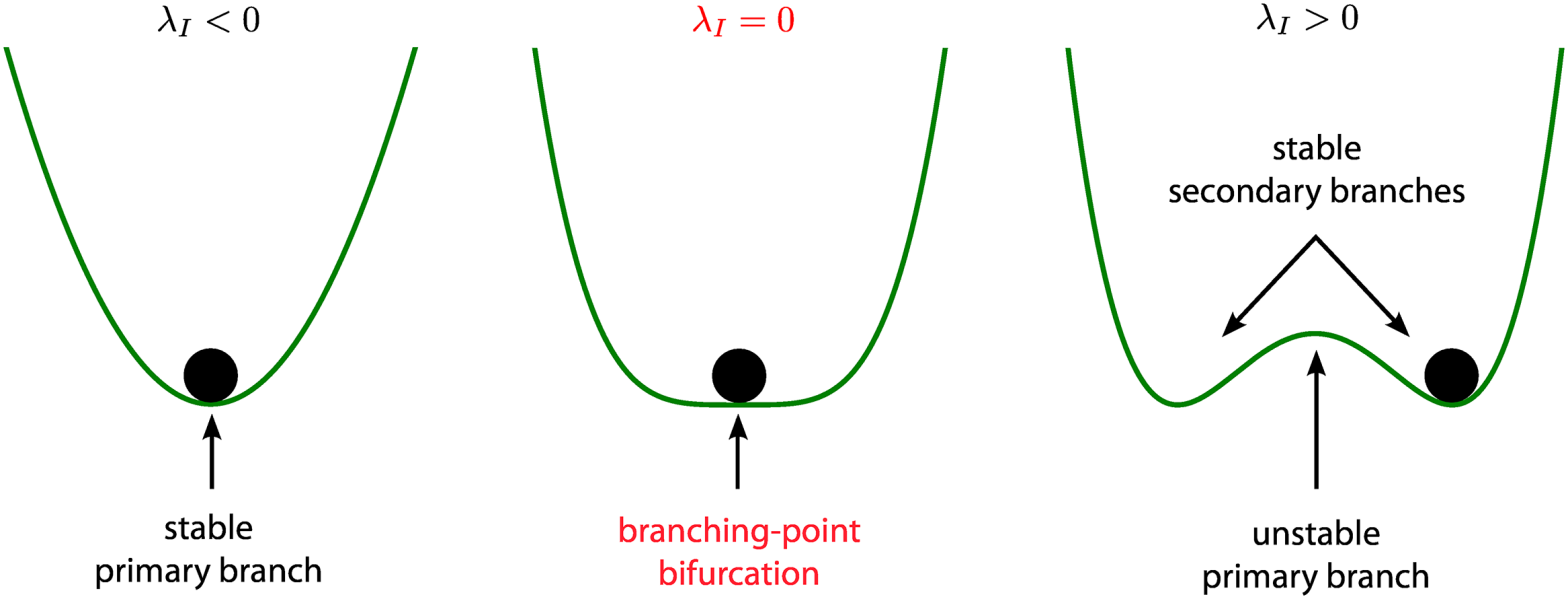
Spontaneous symmetry-breaking under strong inhibition. When *λ*_*I*_ (see Eq (8)) is negative, the system (3) has only one stationary solution for a fixed set of parameters. This solution is stable and symmetric, but if a network parameter (e.g. *I*_*E*_) is allowed to vary continuously, *λ*_*I*_ may change sign. If this occurs, the solution becomes unstable and the network chooses randomly between two new alternative states, breaking the symmetry. The new states can be stable (e.g. for *N*_*I*_ = 2) or unstable. In the former case the phenomenon of spontaneous symmetry-breaking may be understood intuitively as a ball that rolls in a double well potential and reaches a state of minimum energy, which corresponds to a stable stationary solution of Eq (3). In the weak-inhibition regime *λ*_*I*_ is always negative, therefore strong inhibition is a necessary condition for spontaneous symmetry-breaking.

According to bifurcation theory [49], the system undergoes special bifurcations when one of its eigenvalues is equal to zero. In particular, whenever *λ*_*I*_ = 0, the network may undergo a so-called *branching-point bifurcation*. Since *λ*_*I*_ is always negative in the weak-inhibition regime, branching-point bifurcations occur only in the strong-inhibition regime (*ψ* ≥ 1). This special bifurcation corresponds to the formation of heterogeneous membrane potentials in the inhibitory population. In other words, strong inhibition may break the symmetry of the system. Intuitively, this can be understood as in Fig 3. In the weak-inhibition regime there is only one valley or basin in the “energy landscape” of the network. However, strong inhibition may lead to the formation of multiple valleys, and a small perturbation determines the valley the inhibitory potential will converge to. For this reason, for strong inhibition multiple new branches of the equilibrium points may emerge, which are described by the following stationary solutions:

$$\begin{array}{c} \mu =\left(\overset{N_{E}-\text{times}}{\overset{︷}{\mu_{E},\ldots ,\mu_{E}}},\mu_{I,0},\ldots ,\mu_{I,N_{I}-1}\right) \end{array}$$ (10)

where *μ*_*E*_ and *μ*_*I*,*i*_ are the solutions of the following system of algebraic non-linear equations, obtained from Eq (3) in the stationary regime:

$$\begin{array}{c} \{\begin{array}{cc} -\frac{1}{\tau_{E}}\mu_{E}+\frac{N_{E}-1}{N-1}J_{EE}A_{E}\left(\mu_{E}\right)+\frac{J_{EI}}{N-1}\sum_{j=0}^{N_{I}-1}A_{I}\left(\mu_{I,j}\right)+I_{E}=0 & \\ -\frac{1}{\tau_{I}}\mu_{I,i}+\frac{N_{E}}{N-1}J_{IE}A_{E}\left(\mu_{E}\right)+\frac{J_{II}}{N-1}\sum_{\begin{array}{l} j=0\\ j\neq i \end{array}}^{N_{I}-1}A_{I}\left(\mu_{I,j}\right)+I_{I}=0 & \text{for }i=0,\ldots ,N_{I}-1\;. \end{array} \end{array}$$ (11)

For example, for *N*_*I*_ = 2, Eq (11) can be written more explicitly as follows:

$$\begin{array}{c} \left\{\begin{array}{c} F\left(\mu_{E},\mu_{I,0},\mu_{I,1}\right)\overset{\text{def}}{=}-\frac{1}{\tau_{E}}\mu_{E}+\frac{N_{E}-1}{N-1}J_{EE}A_{E}\left(\mu_{E}\right)+\frac{J_{EI}}{N-1}\left[A_{I}\left(\mu_{I,0}\right)+A_{I}\left(\mu_{I,1}\right)\right]+I_{E}=0\\ G\left(\mu_{E},\mu_{I,0},\mu_{I,1}\right)\overset{\text{def}}{=}-\frac{1}{\tau_{I}}\mu_{I,0}+\frac{N_{E}}{N-1}J_{IE}A_{E}\left(\mu_{E}\right)+\frac{J_{II}}{N-1}A_{I}\left(\mu_{I,1}\right)+I_{I}=0\\ H\left(\mu_{E},\mu_{I,0},\mu_{I,1}\right)\overset{\text{def}}{=}-\frac{1}{\tau_{I}}\mu_{I,1}+\frac{N_{E}}{N-1}J_{IE}A_{E}\left(\mu_{E}\right)+\frac{J_{II}}{N-1}A_{I}\left(\mu_{I,0}\right)+I_{I}=0\;. \end{array}\right. \end{array}$$ (12)

**Fig 4:**
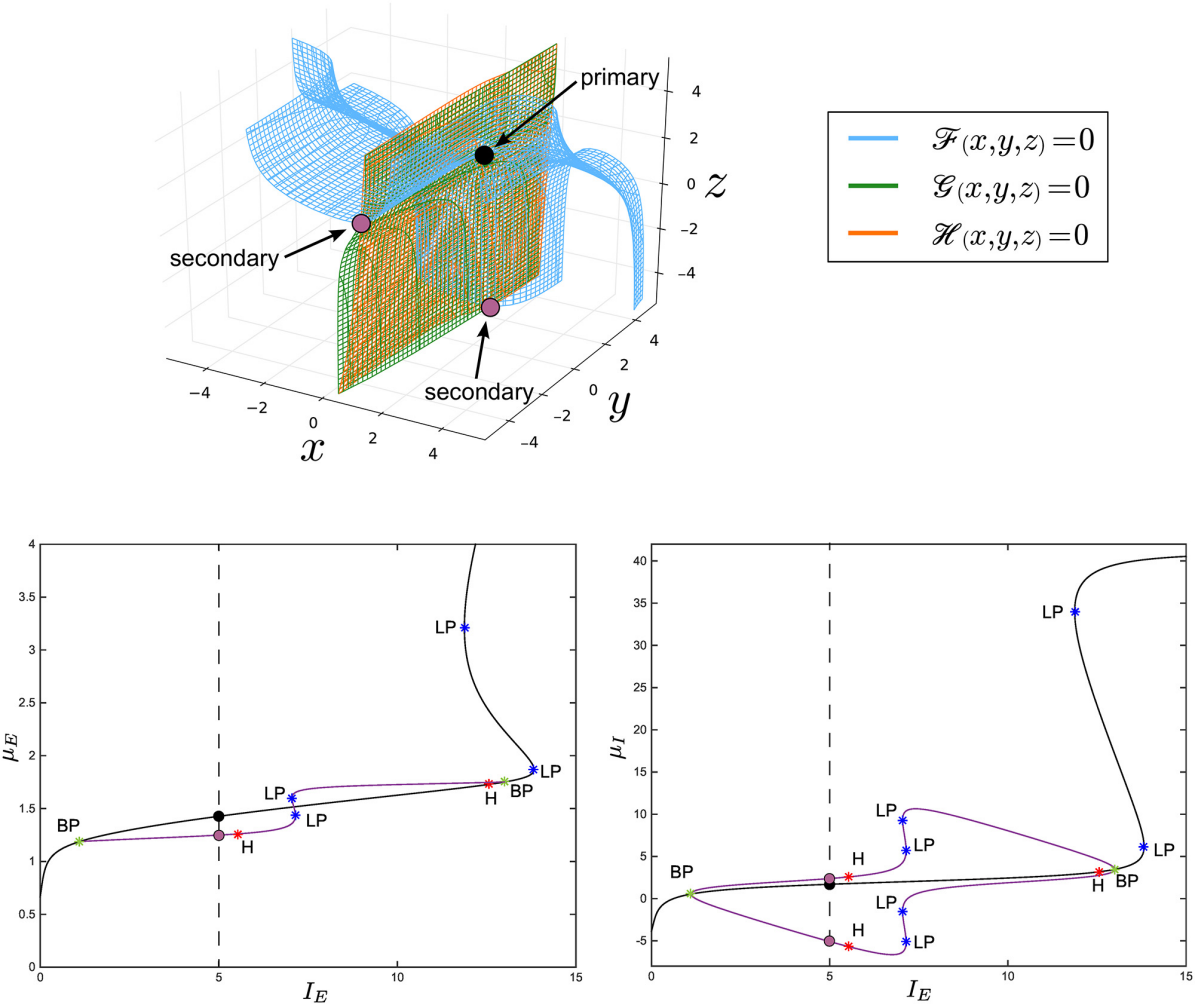
Formation of multiple stationary solutions in the strong-inhibition regime. The top panel shows the nullsurfaces of the network for fixed values of *I*_*E*,*I*_ in the strong-inhibition regime, obtained for *J*_*II*_ = −100, *I*_*E*_ = 5, *I*_*I*_ = −10 and the values of the parameters in Table 1. The black intersection point corresponds to the solution of the system (12) on the primary branch, while the violet dots represent the solutions on the secondary branches. The two figures at the bottom show the solutions *μ*_*E*_ and *μ*_*I*_ (left and right panel, respectively) of the system (12) for the same values of the parameters, but with varying current *I*_*E*_. The black and violet curves represent, respectively, the primary and secondary branches of the network equations. For *I*_*E*_ = 5 the system admits three solutions (*μ*_*E*_,*μ*_*I*_) (see the dots at the intersection with the vertical dashed lines: we have only three solutions because *μ*_*I*_ has three intersection points, two of which, i.e. the violet ones in the right panel, correspond to the same *μ*_*E*_, i.e. the violet dot in the left panel). The (*μ*_*E*_,*μ*_*I*_) coordinates of these solutions correspond to those of the dots in the top panel of the figure. Again, here we do not care about the stability of the solutions, which is shown for the sake of completeness in Fig 9 and in S9 Fig.

The surfaces $F(x,y,z)=0,G(x,y,z)=0,H(x,y,z)=0,\forall \left(x,y,z\right)\in {\mathbb{R}}^{3}$ are a higher-dimensional extension of the nullclines $F(x,y)=0\text{ and }G(x,y)=0\;\forall \left(x,y\right)\in {\mathbb{R}}^{2}$ that we encountered in the weak-inhibition regime. These surfaces have been called *nullsurfaces* in [48]. Fig 4 (top) shows an example obtained for *J*_*II*_ = −100, while the remaining parameters are chosen as in Table 1.

For the sake of clarity, here we treat in detail only the case *N*_*I*_ = 2, and we will discuss briefly some results in the strong-inhibition regime for *N*_*I*_ > 2. For *N*_*I*_ = 2, from Eq (12) the Jacobian matrix on the secondary branches Eq (10) is:

$$\begin{array}{c} J=\left[\begin{array}{ccc} j_{00} & j_{01} & j_{02}\\ j_{10} & j_{11} & j_{12}\\ j_{20} & j_{21} & j_{22} \end{array}\right] \end{array}$$ (13)

where:

$$\begin{array}{l} J_{00}=-\frac{1}{\tau_{E}}{\text{Id}}_{N_{E}}+\frac{J_{EE}}{N-1}A_{E}^{'}\left(\mu_{E}\right)\left(I_{N_{E}}-{\text{Id}}_{N_{E}}\right),\\ J_{01}=\frac{J_{EI}}{N-1}A_{I}^{'}\left(\mu_{I,0}\right)1_{N_{E}},\;J_{02}=\frac{J_{EI}}{N-1}A_{I}^{'}\left(\mu_{I,1}\right)1_{N_{E}},\\ J_{10}=\frac{J_{IE}}{N-1}A_{E}^{'}\left(\mu_{E}\right)1_{N_{E}}^{t},\;J_{11}=\frac{1}{\tau_{I}},\;J_{12}=\frac{J_{II}}{N-1}A_{I}^{'}\left(\mu_{I,1}\right),\\ J_{20}=\frac{J_{IE}}{N-1}A_{E}^{'}\left(\mu_{E}\right)1_{N_{E}}^{t},\;J_{21}=\frac{J_{II}}{N-1}A_{I}^{'}\left(\mu_{I,0}\right),\;J_{22}=\frac{1}{\tau_{I}} \end{array}$$

(here *t* is the transpose operator applied to a matrix), as derived in more detail in S1 Text. Intuitively, on the primary branch the Jacobian matrix was a 2 × 2 block matrix (see Eq (7)), because we had only one excitatory and one inhibitory membrane potential (*μ*_*E*_ and *μ*_*I*_ respectively), while on the secondary branches it is a 3 × 3 block matrix since now the two inhibitory neurons have different potentials (*μ*_*I*,0_ and *μ*_*I*,1_). However, the Jacobian matrix on the new branches can be calculated for a network with an arbitrary number of inhibitory neurons (see S1 Text for more details).

Finally, we observe that for any *N*_*I*_ it is possible to find a relation between any pair (*i*,*j*) of inhibitory membrane potentials in the strong-inhibition regime, which proves very useful when we calculate analytically the local bifurcations of the system on the secondary branches. Indeed, from the second equation of the system (11), we get:

$$\begin{array}{c} -\frac{1}{\tau_{I}}\mu_{I,i}+\frac{J_{II}}{N-1}A_{I}\left(\mu_{I,j}\right)=-\frac{1}{\tau_{I}}\mu_{I,j}+\frac{J_{II}}{N-1}A_{I}\left(\mu_{I,i}\right). \end{array}$$ (14)

After some algebra Eq (14) can be converted into the following fourth-order polynomial equation:

$$\begin{array}{c} \hat{a}\mu_{I,j}^{4}+\hat{b}\mu_{I,j}^{3}+\hat{c}\mu_{I,j}^{2}+\hat{d}\mu_{I,j}+\hat{e}=0 \end{array}$$ (15)

whose coefficients depend on *μ*_*I*,*i*_ as follows:

$$\begin{array}{l} \hat{a}=\frac{\Lambda_{I}^{2}}{4\tau_{I}^{2}}\\ \hat{b}=-\frac{\Lambda_{I}^{2}}{2\tau_{I}}\left(\hat{\phi }+\frac{V_{I}^{T}}{\tau_{I}}\right)\\ \hat{c}=\;\frac{\Lambda_{I}^{2}}{4}\left[{\hat{\phi }}^{2}+{\left(\frac{V_{I}^{T}}{\tau_{I}}\right)}^{2}+\frac{4}{\tau_{I}}V_{I}^{T}\hat{\phi }\right]+\frac{1}{\tau_{I}^{2}}-\hat{\xi }\\ \hat{d}=-\frac{\Lambda_{I}^{2}}{2}\hat{\phi }V_{I}^{T}\left(\frac{V_{I}^{T}}{\tau_{I}}+\hat{\phi }\right)-\frac{2}{\tau_{I}}\hat{\phi }+2\hat{\xi }V_{I}^{T}\\ \hat{e}={\left(\frac{\Lambda_{I}}{2}\hat{\phi }V_{I}^{T}\right)}^{2}+{\hat{\phi }}^{2}-\hat{\xi }{\left(V_{I}^{T}\right)}^{2}\\ \hat{\phi }=\;\frac{1}{\tau_{I}}\mu_{I,i}+\frac{J_{II}}{N-1}A_{I}\left(\mu_{I,i}\right)-\frac{\nu_{I}^{max}J_{II}}{2\left(N-1\right)}\\ \hat{\xi }={\left(\frac{\nu_{I}^{max}\Lambda_{I}J_{II}}{4\left(N-1\right)}\right)}^{2}. \end{array}$$

**Fig 5:**
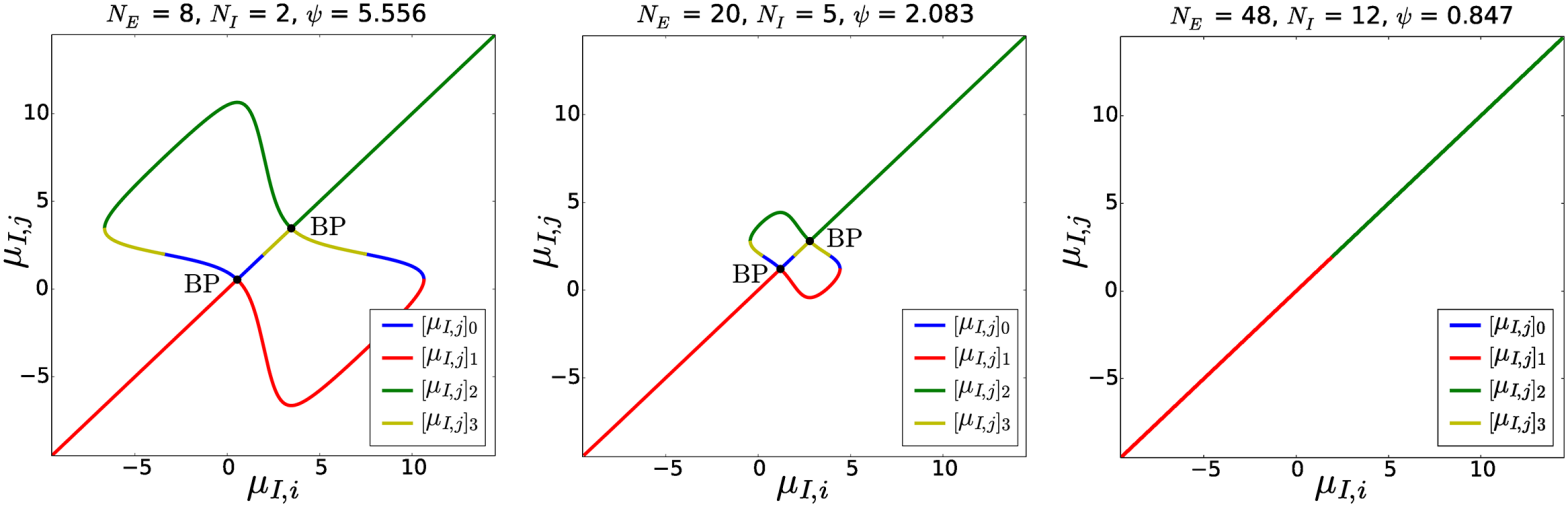
Relation between any pair of inhibitory membrane potentials in the network. This figure is obtained for *J*_*II*_ = −100 by plotting the four solutions [*μ*_*I*,*j*_]_0,1,2,3_ of Eqs (14) + (15) (see S1 Text for their analytical calculation) as a function of *μ*_*I*,*i*_, and proves the formation of three branches of solutions of the stationary membrane potentials at the branching-point bifurcations. For example, in the case *N*_*I*_ = 2 (left panel), the bisector of the first and third quadrants *μ*_*I*,*j*_ = *μ*_*I*,*i*_ represents the primary branch of the network, while the other two solutions that bifurcate from the branching points represent the secondary branches. The coordinates of the branching points are given by Eq (23). For the sake of clarity, we do not show the stability of the solutions, which is examined in the text. Moreover, the figure shows that for increasing *N* these bifurcations disappear, as we discuss in more detail in S1 Text.

Eq (15) can be solved analytically, providing an explicit expression of *μ*_*I*,*j*_ as a function of *μ*_*I*,*i*_, which will be used later to evaluate the local bifurcations in the strong-inhibition regime. In Fig 5 we plot this relation by removing the spurious portions of the solutions of Eq (15) that do not satisfy Eq (14). This figure proves the formation of three branches of solutions of the stationary membrane potentials at the branching-point bifurcations of the network.

### Weak-inhibition regime (*ψ* < 1)

Even though in the article we focus on the case *N*_*E*_ = 8 and *N*_*I*_ = 2, the analytical results derived in this subsection are valid for arbitrary *N*_*E*,*I*_.

As we said, we want to understand how the network’s dynamics changes when we vary the external input currents *I*_*E*,*I*_ and the strength of the synaptic weight *J*_*II*_. In this subsection we show the results that we obtain when we vary these parameters one by one, because this allows us to introduce the concepts of codimension one and codimension two bifurcation diagrams.

**Fig 6:**
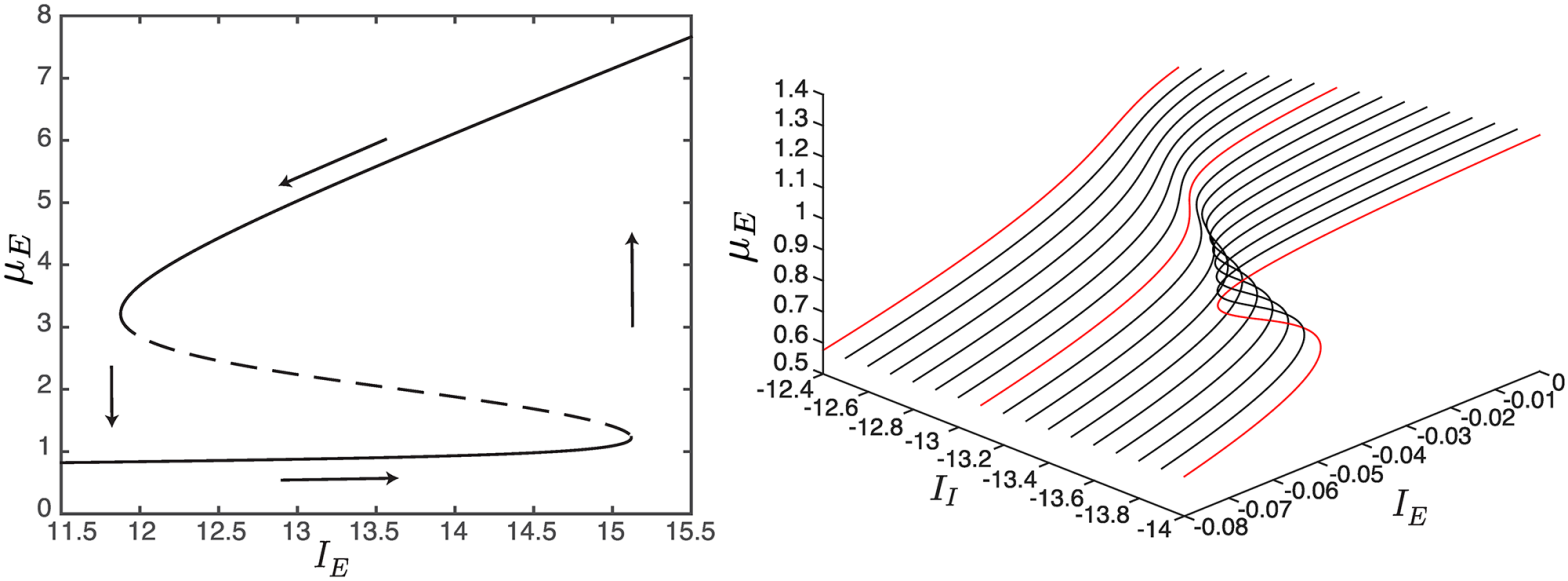
An example of catastrophe manifold. On the left, we show an example of hysteresis displayed by the system. The plain lines describe stable equilibria, while the dashed line the unstable ones. On the right, we show an example of catastrophe manifold. The panel highlights three different behaviors of the network for increasing values of ∣*I*_*I*_∣: leaky integrator, perfect integrator and switch (red curves). In particular, the perfect integrator corresponds to a cusp bifurcation (see Fig 7).

For *N*_*I*_ = 2, we first fix *I*_*I*_ to −10 and the inhibitory strength *J*_*II*_ to −10. As illustrated in Fig 2 (top), Eq (6) admits multiple solutions depending on the specific value chosen for the current *I*_*E*_. Thus, we vary *I*_*E*_ continuously while keeping *I*_*I*_ and *J*_*II*_ fixed. In this way we can plot how the solutions *μ*_*E*,*I*_ of the system (6) change as a function of *I*_*E*_. As we anticipated in the previous subsection, the curve that we obtain (Fig 2 (bottom)) is called the *primary branch* of the network. This figure represents the *codimension one bifurcation diagram* of the network from which we individuate two special points, called *local saddle-node bifurcations* (shortened to LP hereafter). They identify the value of *I*_*E*_ for which the number of solutions of Eq (6) changes (notice the correspondence with the top panels of Fig 2). These are the first examples of (local) bifurcation points that the neural network exhibits, and that lead to the formation of hysteresis (see left panel in Fig 6). Briefly, hysteresis was suggested to describe the reverberation and persistence of neural activity sustained endogenously after an external input is removed, which is thought to be crucial for phenomena such as working memory [62–64]. It is important to observe that even if reverberation requires bistability, the latter can be present without hysteresis, but very often they coexist, as in our model.

**Fig 7:**
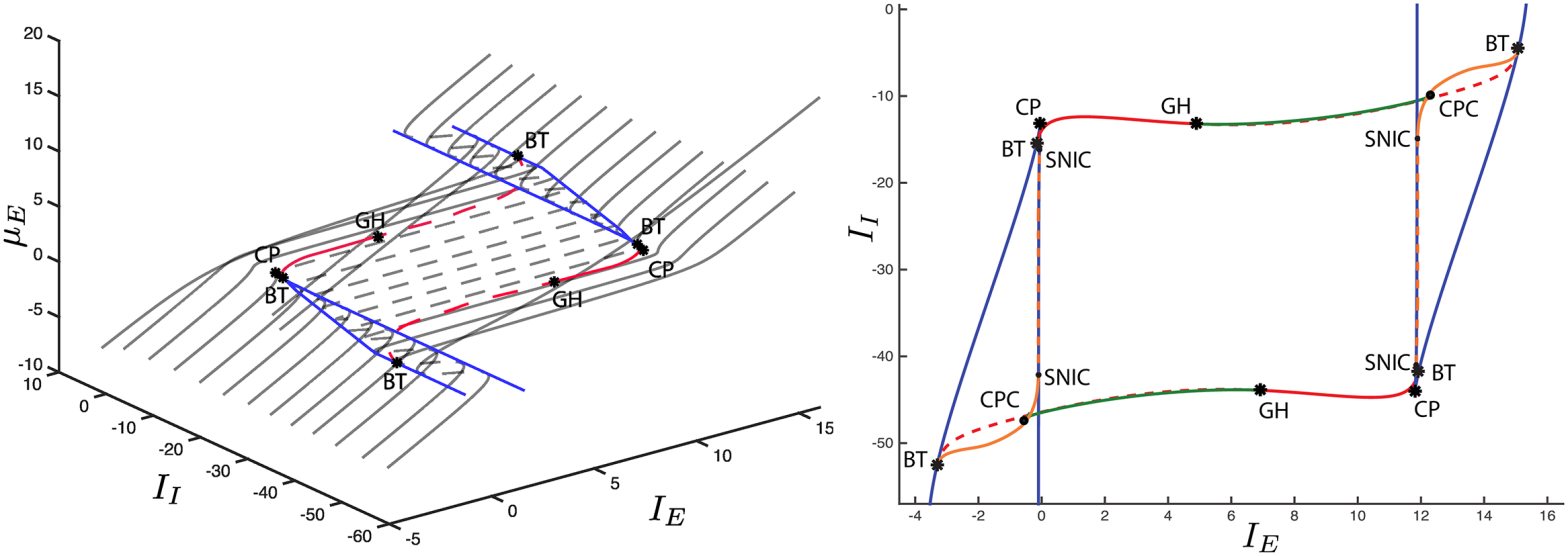
Three- and two-dimensional bifurcation diagrams in the weak-inhibition regime. Left, *μ*_*E*_ is shown on the *z*−axis as function of *I*_*E*_−*I*_*I*_. Here we plot only the local bifurcations (blue: saddle-node curves, red: Andronov-Hopf curves) that bound the plain/dashed regions representing the stable/unstable equilibrium point areas. Right, complete set of codimension two bifurcations. The Andronov-Hopf bifurcation curves (red lines) are divided into supercritical (plain) and subcritical (dashed) portions. The supercritical/subcritical portions are bounded by a generalized Hopf bifurcation, GH, and Bogdanov-Takens bifurcations, BT. The latter are the contact points among saddle-node bifurcation curves (blue lines), Andronov-Hopf bifurcation curves (red lines), and homoclinic bifurcation curves (hyperbolic-saddle/saddle-node homoclinic bifurcations are described by plain/dashed orange curves). SNIC bifurcations identify the contact point between the saddle-node curve and the homoclinic one. From GH originate two limit point of cycles curves (dark green lines) that collapse into the homoclinic curves. Before this, they present a cusp bifurcation, CPC. Each saddle-node curve shows, in addition to BT, a cusp bifurcation, CP.

We then continuously vary both *I*_*E*_ and *I*_*I*_, while keeping *J*_*II*_ fixed, and we plot the solutions of Eq (6) as a function of both the external currents, so that *μ*_*E*,*I*_ = *μ*_*E*,*I*_(*I*_*E*_,*I*_*I*_) now define three dimensional manifolds (see Fig 7 (left) for *μ*_*E*_). On these manifolds, the special points LP depend also on *I*_*I*_, therefore they form a set of points called *saddle-node curves* (see the blue curves in Fig 7, left). For visual convenience, these curves are projected on the *I*_*E*_−*I*_*I*_ plane in Fig 7 (right), defining the so-called *codimension two bifurcation diagram* of the network. This diagram will be the main instrument of the analysis presented hereafter. However, from Fig 7 (left) and the bottom panels of Fig 2, we can see that the saddle-nodes are not the only bifurcations we have in our network. For some values of the pair *I*_*E*_−*I*_*I*_, the system can undergo also a *local Andronov-Hopf bifurcation*, or H for short. These bifurcations are represented by the red curves in Fig 7, and correspond to the emergence of neural oscillations, which are thought to play a key role in many cognitive processes [65].

Hereafter, we list all the bifurcations the system undergoes, dividing them in groups depending on the codimension of the bifurcation, which is defined as the number of parameters (in our case *I*_*E*,*I*_) that must be varied for the bifurcation to occur. Although only few of them are represented in Fig 2 (bottom), the complete set of codimension one bifurcations our system undergoes is:

- local saddle-node bifurcations (LP), for which new equilibria arise, or collide and annihilate each other;
- local Andronov-Hopf bifurcations (H), where stable or unstable self-sustained oscillations, described by limit cycles, arise or disappear;
- global homoclinic bifurcations, where limit cycles vanish in a special equilibrium point (i.e. a neutral saddle, see the next subsection, or an LP bifurcation point), giving rise to an orbit with infinite period;
- global limit point of cycles bifurcations (LPC), at which new limit cycles arise, or collide and annihilate each other.

The codimension one diagrams collecting all these bifurcations are shown in S1 Text. Moreover, on the curves defined by these bifurcations, and that are obtained by varying both *I*_*E*_ and *I*_*I*_, the following codimension two bifurcations appear (see Fig 7, right):

- local cusp bifurcations (CP), on the LP curves;
- local generalized Hopf bifurcations (GH), which divide subcritical H curves from supercritical ones;
- local Bogdanov-Takens bifurcations (BT), which represent the contact point between the LP, H (ending here) and homoclinic curves;
- global cusp bifurcation of cycles (CPC), on the limit point of cycles curves;
- global saddle-node on invariant circle (SNIC), where an LP bifurcation occurs simultaneously with a homoclinic bifurcation.

It is worth remarking that the bifurcation diagram in Fig 7 (right), obtained from the voltage-based model (3) in the weak-inhibition regime, is qualitatively similar to that of the activity-based (mean-field) Wilson-Cowan model (see Fig. 2.12 in [66]). This strong similarity results from the fact that under the hypotheses of stationary inputs, invertible connectivity matrix *J* and homogeneous membrane time constants, the two models are mathematically equivalent (apart from a rescaling by *τ* of the external input). Indeed, it is possible to prove that the membrane potentials *V*_*i*_(*t*) and the neural activities *A*_*i*_(*t*) are formally related by the linear expression $V_{i}(t)=\frac{1}{M_{i}}\sum_{j=0}^{N-1}J_{ij}A_{j}(t)+I_{i}$ [66]. However, in the next subsection we will show that things may change significantly in the strong-inhibition regime if we take into account finite-size effects.

Interestingly, Fig 7 presents two of the so-called *catastrophe manifolds* [67], one of which is shown in the right panel of Fig 6. This figure emphasizes the ability of the model to describe three different behaviors: leaky integrator, perfect integrator and switch. This triad represents the main ingredient for describing a mechanism which was proposed to explain interval timing by neural integration [68, 69]. According to the theory, by changing some parameters of the network, it is possible to modify the shape of the equilibrium curve on the catastrophe manifold generating a ramping activity that can explain Weber’s law of time perception [70]. This phenomenon can easily occur in our model, where the shape of the equilibrium curve can be changed dynamically by varying the input currents.

Now we prove some of our previous results on local bifurcations from the analytical point of view (global bifurcations are often harder to study analytically, therefore they are not considered here). We base our analysis on [5], where the authors developed a technique for deriving analytical expressions of local codimension one bifurcations. Furthermore, for the first time we will extend their method to the analysis of spontaneous symmetry-breaking in systems with arbitrary size and to some local bifurcations with codimension larger than one. We start by observing that, according to bifurcation theory [49, 61], LP is defined by the condition that one real eigenvalue of the Jacobian matrix becomes equal to zero. Therefore, from Eq (8), we conclude that for our network this bifurcation occurs whenever $\lambda_{0}^{R}=0$ or $\lambda_{1}^{R}=0$, because *λ*_*E*_ is always negative, while *λ*_*I*_ is always negative in the weak-inhibition regime. For example, if Y+Z<0, the condition $\lambda_{0}^{R}=0$ is equivalent to X=YZ which, according to Eq (9), provides:

$$\begin{array}{c} A_{I}^{\prime }\left(\mu_{I}\right)=\frac{-\frac{1}{\tau_{E}\tau_{I}}+\frac{1}{\tau_{I}}\frac{N_{E}-1}{N-1}J_{EE}A_{E}^{\prime }\left(v\right)}{-\frac{1}{\tau_{E}}\frac{N_{I}-1}{N-1}J_{II}+\frac{1}{{(N-1)}^{2}}\left[\left(N_{E}-1\right)\left(N_{I}-1\right)J_{EE}J_{II}-N_{E}N_{I}J_{EI}J_{IE}\right]A_{E}^{\prime }\left(v\right)} \end{array}$$ (16)

where we have defined the parameter $v\overset{\text{def}}{=}\mu_{E}$. Now we invert $A_{I}^{\prime }(\mu_{I})$ (more details are provided in S1 Text), obtaining:

$$\begin{array}{c} \mu_{I}\left(v\right)=V_{I}^{T}\pm \frac{2}{\Lambda_{I}}\sqrt{\sqrt[{3}]{{\left(\frac{\nu_{I}^{\text{max}}\Lambda_{I}}{4A_{I}^{\prime }\left(\mu_{I}\right)}\right)}^{2}}}-1 \end{array}$$ (17)

and from Eq (6) we get:

$$\begin{array}{c} \left\{\begin{array}{c} I_{E}\left(v\right)=\frac{1}{\tau_{E}}v-\frac{N_{E}-1}{N-1}J_{EE}A_{E}\left(v\right)-\frac{N_{I}}{N-1}J_{EI}A_{I}\left(\mu_{I}\left(v\right)\right)\\ I_{I}\left(v\right)=\frac{1}{\tau_{I}}\mu_{I}\left(v\right)-\frac{N_{E}}{N-1}J_{IE}A_{E}\left(v\right)-\frac{N_{I}-1}{N-1}J_{II}A_{I}\left(\mu_{I}\left(v\right)\right). \end{array}\right. \end{array}$$ (18)

These are parametric equations in the parameter $v\in (v_{a},v_{b})$, where:

$$\begin{array}{c} v_{b,a}=V_{E}^{T}\pm \frac{2}{\Lambda_{E}}\sqrt{\sqrt[{3}]{{\left(\frac{N_{E}-1}{N-1}J_{EE}\frac{\nu_{E}^{\text{max}}\Lambda_{E}}{4}\tau_{E}\right)}^{2}}}-1 \end{array}$$ (19)

and they define analytically the blue curves in Fig 7 (right) (the same result is obtained if Y+Z>0, in which case the condition $\lambda_{1}^{R}=0$ is equivalent to X=YZ). As we said, this is not sufficient to prove that Eqs (16)–(19) describe LP curves, since we should check also the corresponding non-degeneracy conditions. Nevertheless, we observe a perfect agreement between these analytical curves and those obtained numerically by Cl_MatCont and XPPAUT, therefore for simplicity we do not check the remaining conditions and we leave them to the most technical readers. We adopt the same approach for the remaining bifurcations we are about to describe.

Now we focus on the H bifurcations. According to [49, 61], they appear whenever the network has complex-conjugate purely imaginary eigenvalues. Since *λ*_*E*,*I*_ are always real, this condition can be satisfied only by $\lambda_{0,1}^{R}$, by setting Y+Z=0 and ${\left(Y-Z\right)}^{2}+4X<0$. In particular, from the equation Y+Z=0 we get:

$$\begin{array}{c} A_{I}^{\prime }\left(\mu_{I}\right)=\frac{N-1}{\left(N_{I}-1\right)J_{II}}\left[\frac{1}{\tau_{E}}+\frac{1}{\tau_{I}}-\frac{N_{E}-1}{N-1}J_{EE}A_{E}^{\prime }\left(v\right)\right], \end{array}$$ (20)

where $v\overset{\text{def}}{=}\mu_{E}$ as before. Following the same procedure introduced before for the LP curves, we obtain a set of parametric equations for the pairs *I*_*E*_−*I*_*I*_ that generate the H curves, with parameter $v\in \left[v_{f},v_{d}\right]\cup \left[v_{c},v_{e}\right]$, where:

$$\begin{array}{l} v_{c,d}=\;V_{E}^{T}\pm \frac{2}{\Lambda_{E}}\sqrt{\sqrt[{3}]{{\left(\frac{\nu_{E}^{max}\Lambda_{E}\left(N_{E}-1\right)J_{EE}}{4\left(N-1\right)}\frac{1}{\frac{1}{\tau_{E}}+\frac{1}{\tau_{I}}-\frac{\nu_{I}^{max}\Lambda_{I}\left(N_{I}-1\right)J_{II}}{4\left(N-1\right)}}\right)}^{2}}-1}\\ v_{e,f}=V_{E}^{T}\pm \frac{2}{\Lambda_{E}}\sqrt{\sqrt[{3}]{{\left(\frac{\nu_{E}^{max}\Lambda_{E}}{4z}\right)}^{2}}-1}\\ z=\frac{-b-\sqrt{b^{2}-4ac}}{2a}\\ a={\left(\frac{N_{E}-1}{N-1}J_{EE}\right)}^{2}-\frac{N_{E}N_{I}\left(N_{E}-1\right)}{{\left(N-1\right)}^{2}\left(N_{I}-1\right)}\frac{J_{EE}J_{EI}J_{IE}}{J_{II}}\\ b=-\frac{2}{\tau_{E}}\frac{N_{E}-1}{N-1}J_{EE}+\frac{N_{E}N_{I}}{\left(N-1\right)\left(N_{I}-1\right)}\frac{J_{EI}J_{IE}}{J_{II}}\left(\frac{1}{\tau_{E}}+\frac{1}{\tau_{I}}\right)\\ c=\frac{1}{\tau_{E}^{2}}\;. \end{array}$$ (21)

As described above, BT represents the point where the LP and H curves meet each other, and identifies also the end of the H curve. From the condition $\lambda_{0}^{R}=0$ or $\lambda_{1}^{R}=0$ that defines the LP curves, and the condition $\lambda_{0,1}^{R}=\pm \iota \omega$ (where *ι* represents the imaginary unit) that defines the H curves, we get $\lambda_{0}^{R}=\lambda_{1}^{R}=0$. This is the condition that defines analytically the BT points, or equivalently $v_{\text{BT}}=v_{e,f}$ as given by Eq (21), from which the coordinates of the BT points in the *I*_*E*_−*I*_*I*_ plane can be easily obtained through Eq (6). The remaining local bifurcations (i.e. CP and GH) are analytically intractable.

To conclude this subsection, we describe briefly the effect of the variation of the remaining synaptic weights on the codimension two bifurcation diagram, considering the weights in Table 1 as reference point. Given that their variation does not generate interesting phenomena such as the branching-point bifurcation which is obtained by varying *J*_*II*_, we describe the effects of these parameters succinctly. The reader may verify the following results by the supplemental Python code (see S1 File).

For *J*_*EE*_ ≫ 10, the two LP curves become larger and larger on the *I*_*E*_ axis (i.e. the distance between their vertical asymptotes increases). Moreover, the curves get closer and closer to each other, by shifting on the *I*_*E*_ axis, until they intersect and their oblique parts (i.e. those between the BT points) overlap. If we increase *J*_*EE*_ further, the LP curves split again in two disjoint parts, each one presenting two BT and two CP bifurcations (thus the total number of CP points increases from two to four). Between each pair of BT points (on the same LP curve) there is an H curve. These curves are very close to the corresponding LP curves, and if we increase *J*_*EE*_ further they disappear, together with the BT bifurcations. Thus for very large *J*_*EE*_ we get only two disconnected LP curves, or in other terms, for very strong excitation the oscillatory activity cannot be sustained anymore. Also on the opposite side, namely for weak excitation (i.e. *J*_*EE*_ → 0), the H curves disappear. More precisely, according to Eq (21), these curves vanish whenever the following condition is satisfied:

$$\begin{array}{c} \frac{\nu_{E}^{\text{max}}\Lambda_{E}}{4z}\leq 1\;\text{or}\;b^{2}-4ac\leq 0. \end{array}$$

Moreover, for weak excitation the width on the *I*_*E*_ axis of the LP curves decreases, i.e. the distance between their vertical asymptotes becomes smaller and smaller, until the asymptotes collapse on each other and the LP curves disappear as well. In particular the curves vanish whenever the following condition is satisfied (see Eq (19)):

$$\begin{array}{c} \frac{N_{E}-1}{N-1}J_{EE}\frac{\nu_{E}^{\text{max}}\Lambda_{E}}{4}\tau_{E}\leq 1. \end{array}$$

For ∣*J*_*EI*_∣ ≫ 70, the width of the two LP curves remains almost constant, while the distance between them (and therefore also the length of the H curves) increases continuously. On the other side, for ∣*J*_*EI*_∣ → 0, the two LP curves get closer and closer to each other, until they intersect and then their oblique parts between the BT points overlap. If we decrease ∣*J*_*EI*_∣ further, similarly to the case with large *J*_*EE*_, the two LP curves split in two disjoint parts, each one presenting two BT and two CP bifurcations. For even smaller values of ∣*J*_*EI*_∣, the BT, CP and H bifurcations disappear, while the LP curves disappear for ∣*J*_*EI*_∣ = 0.

For *J*_*IE*_ ≫ 70, the LP curves are stretched vertically and shifted downwards along the *I*_*I*_ axis. Clearly, in the opposite direction (i.e. for *J*_*IE*_ → 0), they are compressed, and while the *I*_*E*_ coordinates of the vertical asymptotes remain almost unchanged with *J*_*IE*_, the two CP points get closer and closer to each other. At the same time, the two H curves tend to overlap. At some value of *J*_*IE*_, the two CP bifurcations touch each other and disappear, thus the two LP curves become tangent. If we further decrease *J*_*IE*_, the two LP curves split again, one over the other, and the BT and H bifurcations disappear.

All the phenomena that we have just described are qualitatively similar for different values of *J*_*II*_, thus they occur also in the strong-inhibition regime for the primary branch.

### Strong-inhibition regime (*ψ* ≥ 1)

**Fig 8:**
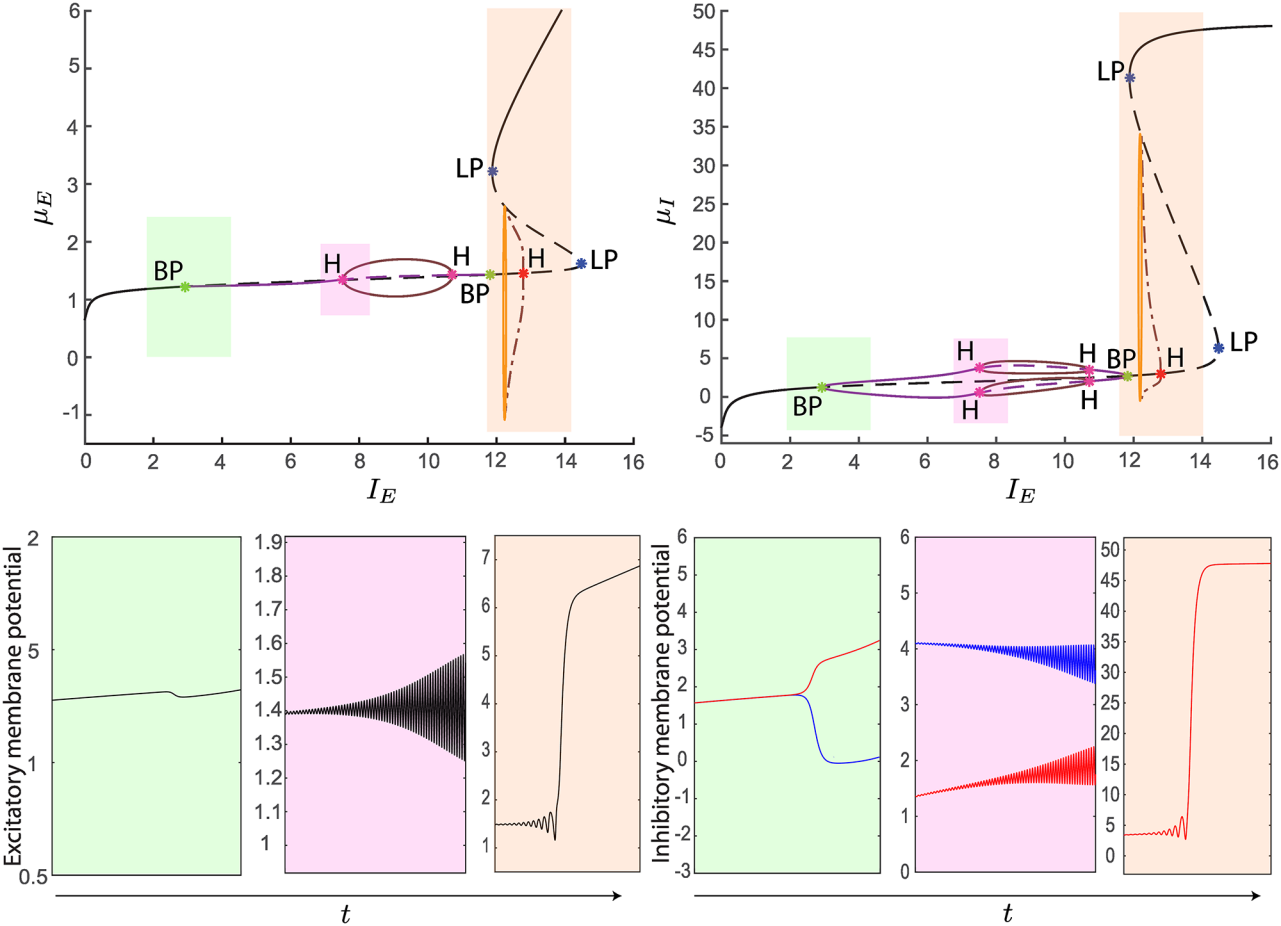
Codimension one bifurcation diagrams for *μ*_*E*_ (left) and *μ*_*I*_ (right) as a function of *I*_*E*_, for *J*_*II*_ = −34 and *I*_*I*_ = −10. In the top panels, the stable/unstable primary equilibrium curve is described by plain/dashed black curves, while the secondary ones are described by plain/dashed violet curves. H bifurcations appear on both the primary (red) and secondary (purple) equilibrium curves, giving rise to unstable and stable limit cycles respectively (the maxima and minima of the oscillations are described by dashed and plain brown curves respectively). In particular, the unstable cycles collapse into a homoclinic bifurcation, described by an orange line. The shaded colored boxes emphasize sampled areas of the codimension one bifurcation diagram, whose corresponding temporal dynamics is shown in the bottom-panels. In particular, the figure shows the correspondence between the BP, H and LP bifurcations and the split of the inhibitory membrane potentials, the emergence of oscillations and the sudden jump of the neural activity, respectively. We induced transitions between these different kinds of dynamics by increasing linearly the external input current *I*_*E*_ while keeping *I*_*I*_ fixed.

**Fig 9:**
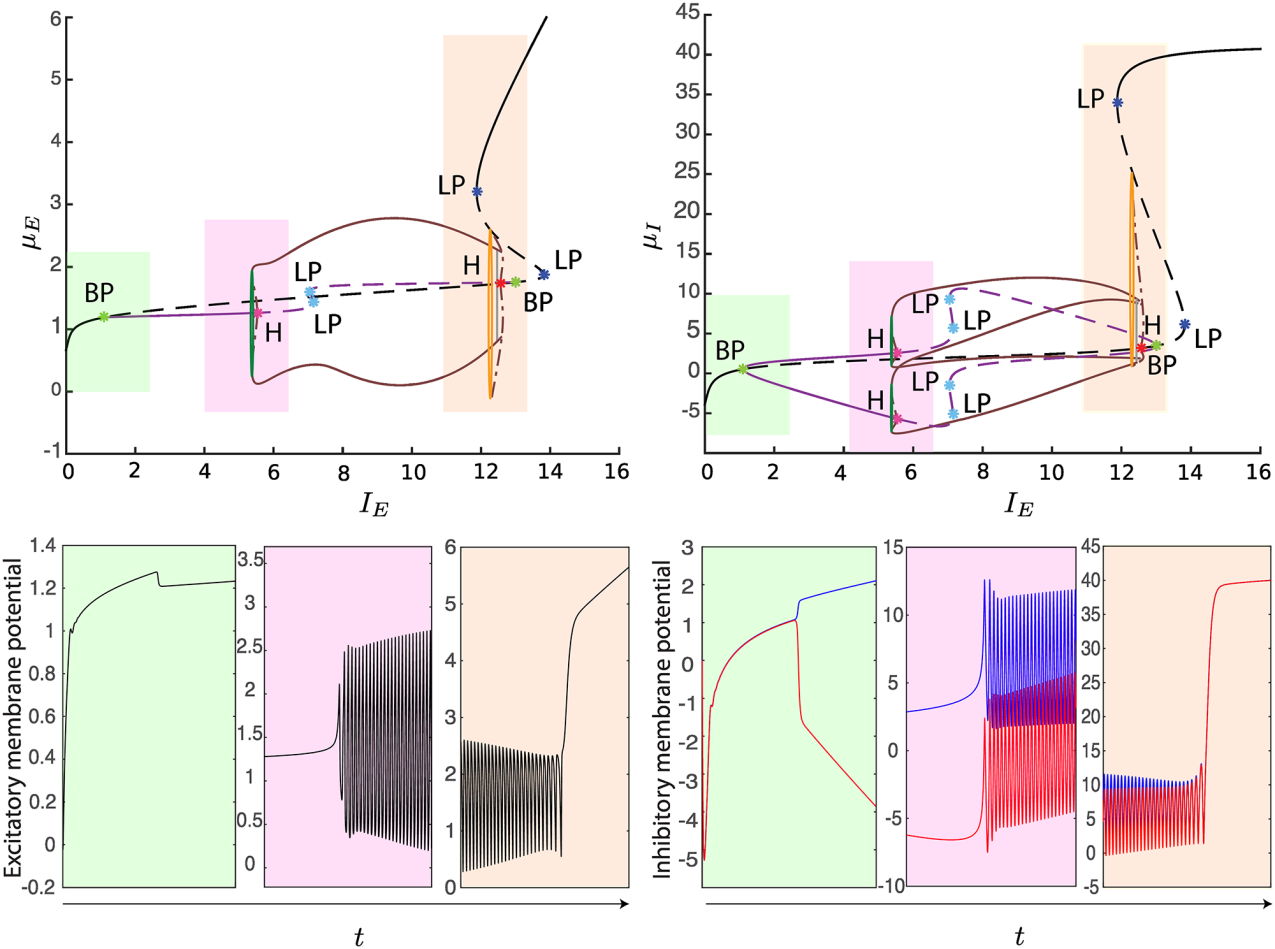
Codimension one bifurcation diagrams for *μ*_*E*_ (left) and *μ*_*I*_ (right) as a function of *I*_*E*_, for *J*_*II*_ = −100 and *I*_*I*_ = −10. The colored curves in the top panels describe the same bifurcations as in Fig 8. Besides, we observe LP bifurcations on the secondary branches (light blue points), an LPC bifurcation (dark green loop), and a TR bifurcation (gray loop). To conclude, similarly to Fig 8, in the bottom panels we plot some examples of dynamics of the membrane potentials for linearly increasing values of the current *I*_*E*_.

**Fig 10:**
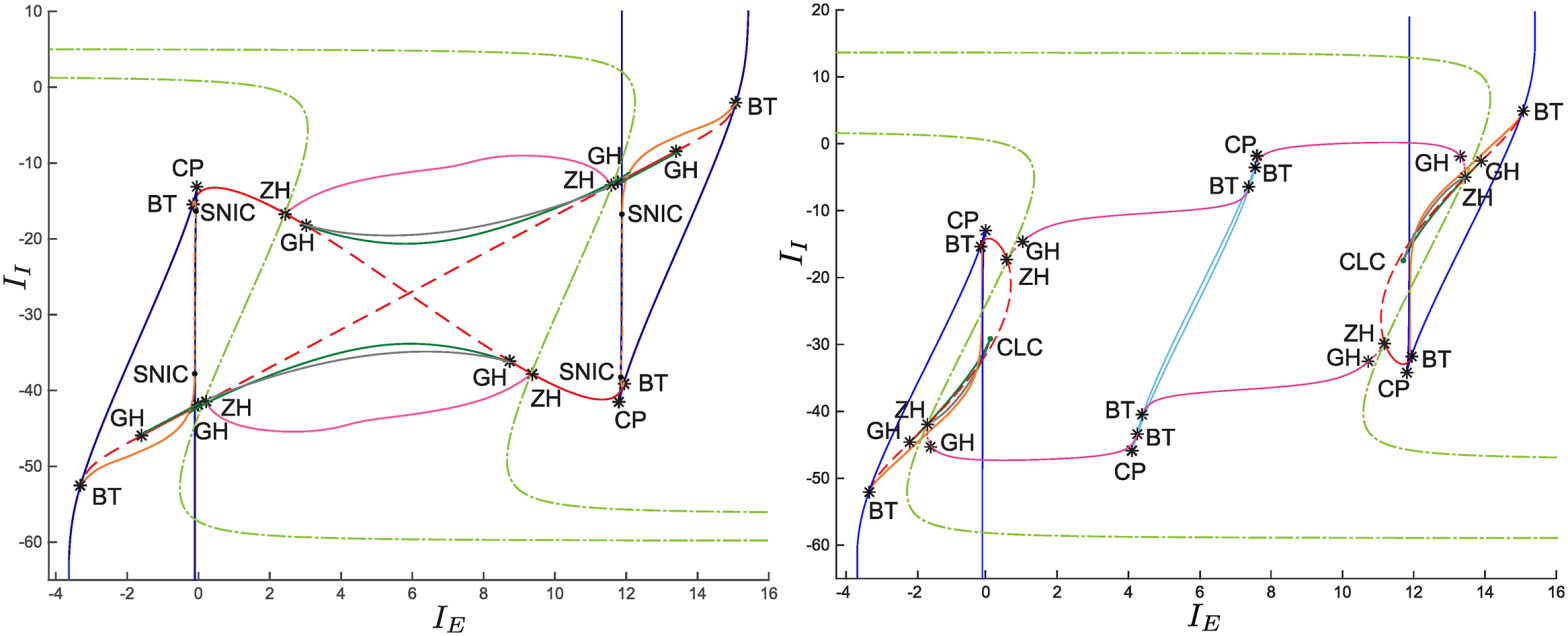
Codimension two bifurcation diagram on the *I*_*E*_−*I*_*I*_ plane for *J*_*II*_ = −34 (left) and *J*_*II*_ = −100 (right). In addition to the bifurcations already displayed in Fig 7 (right) we stress the presence of new ones. The branching points form two curves (light green dot-dashed lines) that define the values of *I*_*E*_−*I*_*I*_ that bound the secondary branches of equilibrium points (see the violet curves in Figs (8) and (9)). The bifurcations originated on the secondary branches are differentiated from those originated on the primary one. Specifically, we show H and LP curves (purple and light blue lines, respectively). In addition, we display the torus bifurcation curves (gray lines).

In the strong-inhibition regime (in particular here we consider the cases *J*_*II*_ = −34 and *J*_*II*_ = −100), most of the features of the weak-inhibition bifurcation diagram are preserved. However, besides the bifurcations explained in the previous subsection, from Figs 8, 9 and 10, we can see that the system undergoes also the following codimension one bifurcations:

- local branching-point bifurcations (BP), at which two or more equilibrium curves emanate or collapse;
- local torus bifurcations (TR), at which the network exhibits quasi-periodic oscillations; this is a local bifurcation of the Poincaré map of a limit cycle of the network [49], whose change of stability determines the formation of solutions of Eq (3) containing two incommensurable frequencies;

and the following codimension two bifurcations:

- local zero-Hopf (neutral saddle) bifurcations (ZH), at which the H curves and the BP curves intersect each other. This point identifies a change in stability of the H bifurcation curve.

In particular, the BP bifurcations lead our model to show multiple branches of stationary solutions for suitable current values. This is a finite-size effect due to the finite number of neurons in each population, which leads to a richer set of eigenvalues than that obtained by using methods based on the reduction of the number of equations, such as the mean-field approximation (see S1 Text for more details). In order to thoroughly investigate the bifurcations the system undergoes in presence of strong inhibition, we start by analyzing the codimension one bifurcation diagram for *J*_*II*_ = −34. In particular, the diagram in Fig 8 is obtained by setting *I*_*I*_ = −10. It turns out that, in addition to the primary equilibrium curve (black line), new branches of stationary states (violet lines) emanate and collapse in two BP bifurcations. These secondary branches hold supercritical H bifurcations that give rise to stable limit cycles. Instead, on the primary branch, we find two LP bifurcations and a subcritical H bifurcation, whose unstable limit cycles vanish in a homoclinic orbit. We also observe that the inhibitory neurons, as well as the excitatory neurons, for *N*_*I*_ = 2 have the same bifurcation diagram (see Fig 8). However, differently from the excitatory case, this does not mean that the inhibitory membrane potentials are homogeneous. Indeed, when an inhibitory neuron is on the upper secondary branch (see the violet curve above the primary equilibrium curve in Fig 8, right), the other one is on the lower secondary branch, thus they are heterogeneous.

For *J*_*II*_ = −100, secondary branches of equilibrium points are still present, see Fig 9. Together with a subcritical H bifurcation, they unveil also LP bifurcations. In particular, the former generates unstable limit cycles that become stable after having crossed the limit point of cycles bifurcation (dark green loop). For increasing values of *I*_*E*_, the stable limit cycles collapse into the unstable limit cycles originated from the subcritical H bifurcation belonging to the primary equilibrium curve. Before collapsing, the stable limit cycles undergo a torus bifurcation (gray loop).

By varying also *I*_*I*_, we obtain the codimension two bifurcation diagrams displayed in Fig 10. It is worth noting that the branching points the system undergoes generate two bifurcation curves (light green dot-dashed lines) that pass through the whole *I*_*E*_−*I*_*I*_ domain. The presence of these curves is the most relevant difference with the weak-inhibition regime and the classic (mean-field) Wilson-Cowan model (compare with Fig. 2.12 in [66]). Furthermore, the LP and H bifurcations that belong to the secondary branches give rise to further bifurcation curves (purple and light blue lines, respectively) in the *I*_*E*_−*I*_*I*_ domain, as shown in Fig 10.

Interestingly, since the BP bifurcation increases the dimension of the network from 2 (for *λ*_*I*_ < 0, see Eq (6)) to 3 (for *λ*_*I*_ > 0, see Eq (12)), the network can exhibit more complex dynamics, such as quasi-periodic motions originated from the torus bifurcations. The biological importance of quasi-periodic oscillations in neural communication was discussed in [71].

Now we want to study the local bifurcations from the analytical point of view. On the primary branch, the LP, H and BT bifurcations have the same expressions obtained in the weak-inhibition regime, therefore they are valid for arbitrary *N*_*E*,*I*_, as well as the formulas that we derive below for the BP and ZH bifurcations. On the contrary, unlike the weak-inhibition regime, the formulas of the LP, H and BT bifurcations on the secondary branches in the strong-inhibition regime are valid only for *N*_*I*_ = 2 (while *N*_*E*_ can still be arbitrary), even though our formalism can be extended to the case *N*_*I*_ > 2.

We start by considering the BP bifurcations, which are defined by the condition *λ*_*I*_ = 0, as we saw before. From Eq (8) this condition implies:

$$\begin{array}{c} A_{I}^{\prime }\left(\mu_{I}\left(\text{BP}\right)\right)=\frac{N-1}{\tau_{I}\left|J_{II}\right|}\;, \end{array}$$ (22)

thus the solutions of this equation are:

$$\begin{array}{c} \mu_{I}\left(\text{BP}\right)=V_{I}^{T}\pm \frac{2}{\Lambda_{I}}\sqrt{\sqrt[{3}]{\psi^{2}}-1}\;. \end{array}$$ (23)

Eq (23) shows that *μ*_*I*_ (BP) is defined only for *ψ* ≥ 1, and therefore that the BP bifurcations occur only in the strong-inhibition regime. Now, from the second equation of the system (6) (we can also use Eq (12), since for *λ*_*I*_ = 0 they are equivalent) we get:

$$\begin{array}{c} \mu_{E}\left(\text{BP}\right)=V_{E}^{T}\pm \frac{2}{\Lambda_{E}}\sqrt{\frac{1}{\frac{1}{{\left\{\frac{2\left(N-1\right)}{\nu_{E}^{\text{max}}N_{E}J_{IE}}\left[\frac{1}{\tau_{I}}\mu_{I}\left(\text{BP}\right)-\frac{N_{I}-1}{N-1}J_{II}A_{I}\left(\mu_{I}\left(\text{BP}\right)\right)-I_{I}\right]-1\right\}}^{2}}-1}} \end{array}$$ (24)

while from the first equation of Eq (6) we get:

$$\begin{array}{c} I_{E}=\frac{1}{\tau_{E}}\mu_{E}\left(\text{BP}\right)-\frac{N_{E}-1}{N-1}J_{EE}A_{E}\left(\mu_{E}\left(\text{BP}\right)\right)-\frac{N_{I}}{N-1}J_{EI}A_{I}\left(\mu_{I}\left(\text{BP}\right)\right) \end{array}$$ (25)

where *μ*_*I*_ (BP) and *μ*_*E*_ (BP) are given by Eqs (23) and (24) respectively. Since *μ*_*E*_ (BP) depends on *I*_*I*_, Eq (25) defines two explicit functions $I_{E}=F_{\pm }(I_{I})$, that provide the curves on which we have a BP bifurcation (see the light green lines in Fig 10 for *J*_*II*_ = −34 and *J*_*II*_ = −100. More details can be found in S1 Text).

It is important to observe that for *N*_*I*_ > 2 the network may undergo special branching points that are not determined by the condition *λ*_*I*_ = 0, rather by the fact that one of the eigenvalues of the reduced Jacobian matrix introduced in S1 Text tends to zero. These branching points can be studied analytically through our approach, but since the complexity of the corresponding eigenvalues strongly depends on *N*_*I*_ and on the degree of heterogeneity of the inhibitory population, we do not analyze them in detail.

The points where the H and BP curves meet each other define the ZH bifurcations. From this definition, we see that they can be calculated analytically from the conditions $\lambda_{0,1}^{R}=\pm \iota \omega$ and *λ*_*I*_ = 0, from which in turn we get:

$$\begin{array}{l} A_{E}^{\prime }\left(\mu_{E}\left(\text{ZH}\right)\right)=\frac{N-1}{\left(N_{E}-1\right)J_{EE}}\left(\frac{1}{\tau_{E}}+\frac{N_{I}}{\tau_{I}}\right)\\ A_{I}^{\prime }\left(\mu_{I}\left(\text{ZH}\right)\right)=\frac{N-1}{\tau_{I}\left|J_{II}\right|} \end{array}$$

and therefore:

$$\begin{array}{l} \mu_{E}^{\pm }\left(\text{ZH}\right)=V_{E}^{T}\pm \frac{2}{\Lambda_{E}}\sqrt{\sqrt[{3}]{{\left(\frac{\nu_{E}^{\text{max}}\Lambda_{E}\left(N_{E}-1\right)J_{EE}}{4\left(N-1\right)\left(\frac{1}{\tau_{E}}+\frac{N_{I}}{\tau_{I}}\right)}\right)}^{2}}-1}\\ \mu_{I}^{\pm }\left(\text{ZH}\right)=V_{I}^{T}\pm \frac{2}{\Lambda_{I}}\sqrt{\sqrt[{3}]{{\left(\frac{\nu_{I}^{\text{max}}\Lambda_{I}\tau_{I}\left|J_{II}\right|}{4\left(N-1\right)}\right)}^{2}}-1}\;. \end{array}$$

As usual, if we substitute these expressions of the membrane potentials into Eqs (6) or (12), we obtain the coordinates of the ZH points in the *I*_*E*_−*I*_*I*_ plane.

On the secondary branches that are generated by the branching points, new bifurcations can occur (in the case *N*_*I*_ = 2, see for example the LP and H bifurcations in Figs (8) and (9), and the corresponding light blue and purple curves in Fig 10), also new branching points (for *N*_*I*_ > 2), from which tertiary branches emerge, and so on. To study them, according to bifurcation theory, we need the Jacobian matrix of the network on the secondary (tertiary, and so on) branches, as we will explain more clearly in the next subsection. Here we focus again on the case *N*_*I*_ = 2, therefore we can determine the local bifurcations on the secondary branches by means of Eq (13).

Now we start with the LP bifurcations. We know that they are defined by the condition that one of the eigenvalues of Eq (13) is equal to zero. From it, as explained in S1 Text, we obtain that:

$$\begin{array}{c} A_{E}^{\prime }\left(\mu_{E}\right)=\frac{\overset{´}{b}}{\overset{´}{a}} \end{array}$$ (26)

where:

$$\begin{array}{l} \begin{array}{l} \overset{´}{a}=\frac{1}{\tau_{I}^{2}}\frac{N_{E}-1}{N-1}J_{EE}+\frac{1}{\tau_{I}}\frac{N_{E}}{{\left(N-1\right)}^{2}}J_{EI}J_{IE}\left[A_{I}^{\prime }\left(\mu_{I,0}\right)+A_{I}^{\prime }\left(\mu_{I,1}\right)\right]\\ \;+\frac{1}{{\left(N-1\right)}^{3}}\left[2N_{E}J_{EI}J_{IE}J_{II}-\left(N_{E}-1\right)J_{EE}J_{II}^{2}\right]A_{I}^{\prime }\left(\mu_{I,0}\right)A_{I}^{\prime }\left(\mu_{I,1}\right) \end{array}\\ \overset{´}{b}=\frac{1}{\tau_{E}}\left[\frac{1}{\tau_{I}^{2}}-{\left(\frac{J_{II}}{N-1}\right)}^{2}A_{I}^{\prime }\left(\mu_{I,0}\right)A_{I}^{\prime }\left(\mu_{I,1}\right)\right]. \end{array}$$

Therefore if we invert Eq (26) and we use the solution of Eq (15), we obtain the expression of *μ*_*E*_ as a function of *μ*_*I*,0_. If we replace the solutions *μ*_*E*_ and *μ*_*I*,1_ in the system (12), we get parametric equations for *I*_*E*,*I*_ as a function of a single parameter, which is now defined as $v\overset{\text{def}}{=}\mu_{I,0}$. These equations are an analytical description of the light blue curves shown in Fig 10 (right) for *J*_*II*_ = −100. Similarly, for the H bifurcations we obtain the following condition:

$$\begin{array}{c} A_{E}^{\prime }\left(\mu_{E}^{\pm }\right)=\frac{-\overset{`}{b}\pm \sqrt{{\overset{`}{b}}^{2}-4\overset{`}{a}\overset{`}{c}}}{2\overset{`}{a}} \end{array}$$ (27)

where:

$$\begin{array}{l} \overset{`}{a}=\frac{N_{E}-1}{N-1}J_{EE}\left[\frac{2}{\tau_{I}}\frac{N_{E}-1}{N-1}J_{EE}+\frac{N_{E}}{{\left(N-1\right)}^{2}}J_{EI}J_{IE}\left(A_{I}^{\prime }\left(\mu_{I,0}\right)+A_{I}^{\prime }\left(\mu_{I,1}\right)\right)\right]\\ \begin{array}{l} \overset{`}{b}=2\frac{N_{E}}{{\left(N-1\right)}^{3}}J_{EI}J_{IE}J_{II}A_{I}^{\prime }\left(\mu_{I,0}\right)A_{I}^{\prime }\left(\mu_{I,1}\right)-\\ \;\left(\frac{1}{\tau_{E}}+\frac{1}{\tau_{I}}\right)\left[\frac{4}{\tau_{I}}\frac{N_{E}-1}{N-1}J_{EE}+\frac{N_{E}}{{\left(N-1\right)}^{2}}J_{EI}J_{IE}\left(A_{I}^{\prime }\left(\mu_{I,0}\right)+A_{I}^{\prime }\left(\mu_{I,1}\right)\right)\right] \end{array}\\ \overset{`}{c}=\frac{2}{\tau_{I}}\left[{\left(\frac{1}{\tau_{E}}+\frac{1}{\tau_{I}}\right)}^{2}-{\left(\frac{J_{II}}{N-1}\right)}^{2}A_{I}^{\prime }\left(\mu_{I,0}\right)A_{I}^{\prime }\left(\mu_{I,1}\right)\right], \end{array}$$

therefore again it is possible to describe these bifurcations analytically, obtaining the same results we got numerically in Fig 10 for *J*_*II*_ = −34 and *J*_*II*_ = −100 (see the purple curves in both the panels). However, unlike the primary branch, our theory does not allow us to calculate the range of the parameter $v=\mu_{I,0}$ on the secondary branches, since the resulting equations that define the range are analytically intractable. In the same way, it is not possible to calculate explicitly the coordinates of the new BT bifurcations, where the LP and H curves that emanate from the secondary branches meet each other. Therefore they can be evaluated only through analytical approximations (which are beyond the purpose of the article), or through a numerical approach (as in S1 File).

For an arbitrary *N*_*I*_, if spontaneous symmetry-breaking has not occurred, and if the initial conditions are homogeneous, then all the neurons in each population are described by the same equation. Therefore the network can be interpreted as a two-dimensional system, and for the Poincaré-Bendixson theorem it cannot show chaotic behavior. Nonetheless, when a symmetry-breaking occurs, the inhibitory neurons split in subgroups, and the network becomes at least three-dimensional depending on the number of inhibitory subpopulations arisen. In this case, it is possible to check numerically the presence of chaos by computing the Lyapunov exponents [62]. In particular, when the symmetry-breaking occurred in the simplest case *N*_*I*_ = 2, we excluded the presence of chaos since the Lyapunov exponents were always negative. However, the emergence of chaos is still possible for *N*_*I*_ > 2, as we will discuss in the next subsection.

We conclude by describing briefly the effect of the variation of the remaining synaptic weights. When we discussed the weak-inhibition regime, we showed that the LP and H curves on the primary branch change in a qualitatively similar way for different values of *J*_*II*_, and the same conclusions apply to the strong-inhibition regime. On the other side, now we want to analyze the behavior of the BP curves and of the bifurcations on the secondary branches. For *J*_*II*_ = −100 and increasing *J*_*EE*_, the most notable phenomenon is the overlap between the oblique parts of the BP and the LP curves of the primary branch. The latter finally collapse on each other and split in two disjoint parts, as in the case *J*_*II*_ = −10 discussed for the weak-inhibition regime, while the bifurcations on the secondary branches do not show any interesting variation. Furthermore, when *J*_*EE*_ → 0, we observe first of all the disappearance of the ZH bifurcations. This occurs because the H curves on both the primary and the secondary branches do not meet the BP curve anymore. If we further decrease *J*_*EE*_, the two CP bifurcations on the LP curve of the secondary branches get closer and closer until they annihilate each other and the curve disappears. This phenomenon implies also the disappearance of the BT bifurcations on the secondary branches. For smaller values of *J*_*EE*_, the H curves on both the primary and secondary branches disappear, and finally also the LP curve on the primary branch (see the weak-inhibition regime) and the BP curve. To conclude, for large ∣*J*_*EI*_∣ or large *J*_*IE*_, the LP curve on the secondary branches disappears again through the annihilation of its CP points (as explained above), while on the other side, when at least one of the two synaptic weights is small, we do not observe any interesting variation of the bifurcations on the secondary branches.

### The study of networks with arbitrary *N*_*I*_

The same analysis can be performed on networks with an arbitrary number *N*_*I*_ of inhibitory neurons. When *λ*_*I*_ in Eq (8) goes to zero, we observed the formation of secondary branches from the primary one. This means that the inhibitory neurons split into two subsets [52, 78], and all the neurons in a given subset behave identically. For this reason we can reinterpret the system as a network with an excitatory population with *N*_*E*_ neurons, and two inhibitory populations. Furthermore, when we change the current *I*_*E*_ (while keeping *λ*_*I*_ > 0) with *I*_*I*_ fixed, at some point one of the eigenvalues of the Jacobian matrix of the system (11) (see S1 Text for their analytical calculation, supposing that the sizes of the clusters are known) may go to zero, generating a new branching point on the secondary branches. In this case we observe the formation of tertiary branches, and so on.

Whenever a group of *K* inhibitory neurons splits into two clusters with sizes *P* and *Q* = *K*−*P* and membrane potentials $\mu_{I}^{\left(P\right)}$ and $\mu_{I}^{\left(Q\right)}$ respectively, the Jacobian matrix of the network has eigenvalues $\lambda_{I}^{\left(P\right)}=-\left[\frac{1}{\tau_{I}}+\frac{J_{II}}{N-1}A_{I}^{\prime }\left(\mu_{I}^{\left(P\right)}\right)\right]$ and $\lambda_{I}^{\left(Q\right)}=-\left[\frac{1}{\tau_{I}}+\frac{J_{II}}{N-1}A_{I}^{\prime }\left(\mu_{I}^{\left(Q\right)}\right)\right]$ with multiplicity *Q*−1 and *P*−1 respectively. Immediately beyond the BP bifurcation that originated the two clusters, one of $\lambda_{I}^{\left(P\right)}$ and $\lambda_{I}^{\left(Q\right)}$ is positive. Thus if *P* and/or *Q* is larger than one, the new branches are unstable near the bifurcation point. These eigenvalues do not affect the stability of the new branches only in the case *P* = *Q* = 1 (having multiplicity equal to zero), which is compatible with the fact that for *N*_*I*_ = 2 the secondary branches are stable near the branching point (see Figs 8 and 9). In the general case *P* = *Q* = 1 the stability of the new branches depends on the eigenvalues of the reduced Jacobian matrix introduced in S1 Text. These eigenvalues are strongly affected by the value of the parameter *N*_*I*_ and by the degree of heterogeneity of the inhibitory population. For this reason it is not easy to draw general conclusions about the stability of the new branches in the case *P* = *Q* = 1.

**Fig 11:**
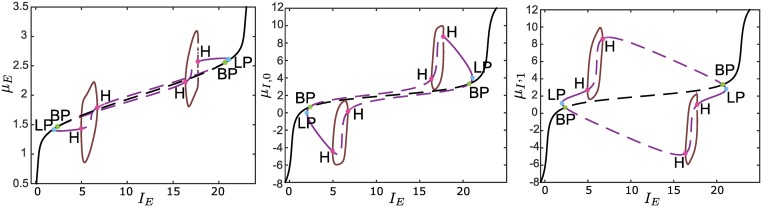
Codimension one bifurcation diagrams for *N*_*I*_ = 4. The left panel shows the diagram of the excitatory neurons, the central panel that of three inhibitory neurons, while the right panel shows the diagram of the remaining neuron in the inhibitory population. The secondary branches are unstable near the BP bifurcations, see text. Compared to the case *N*_*I*_ = 2, now the inhibitory neurons have not only different membrane potentials, but also different codimension one bifurcation diagrams.

In Fig 11 we show an example of formation of secondary branches in the case *N*_*I*_ = 4, obtained as usual for the values of the parameters reported in Table 1. In this case *K* = 4, *P* = 3 and *Q* = 1, therefore the secondary branches are unstable near the branching points.

It is apparent there is an important difference compared to the case *N*_*I*_ = 2. For a network with only two inhibitory neurons, Eq (12) implies that they both have the same codimension one bifurcation diagram (see the right panels of Figs 8 and 9). This is just a special case, because in general, for *N*_*I*_ > 2, the inhibitory neurons have different codimension one bifurcation diagrams. Although Fig 11 is useful to get an idea of how a bifurcation diagram for *N*_*I*_ = 4 would look like, it is important to underline that this diagram could not be entirely thorough. Indeed, since in this case *λ*_*I*_ in Eq (8) has multiplicity larger than one, the Cl_MatCont toolbox may fail in providing us the complete picture [78]. Nonetheless, in principle it is possible to check the completeness of the diagram since, for an arbitrary *N*_*I*_, local bifurcations can still be calculated analytically through our approach. In fact, from Eqs (14) + (15), it is possible to express *N*_*I*_−1 inhibitory membrane potentials as functions of the remaining one, which can be used as a parameter for the parametric equations in the codimension two bifurcation diagram.

To conclude, we observe that for *N*_*I*_ > 2 new kinds of bifurcations may appear, which do not occur in the case *N*_*I*_ = 2. For example, for *N*_*I*_ = 3, if the network has three different inhibitory potentials, the characteristic equation of the Jacobian matrix has the form $p(\lambda )={\left(\lambda -\lambda_{E}\right)}^{N_{E}-1}p_{R}(\lambda )$, where $p_{R}(\lambda )$ (the characteristic polynomial of the reduced Jacobian matrix introduced in S1 Text) is a fourth order polynomial. This means that in principle, for some values of the parameters, the network may have two pairs of purely imaginary eigenvalues. This condition corresponds to the formation of a *double-Hopf bifurcation*, which in turn may imply a local birth of *chaos* (see for example [72, 73]).

## Discussion

We proved the emergence of complex dynamics in small neural circuits, characterized by strong finite-size effects, which cannot be accounted for by the mean-field approximation and that are much less likely to occur in large fully-connected neural systems. We showed, through a detailed numerical and analytical study of the bifurcations, that small homogeneous neural networks undergo spontaneous symmetry-breaking through branching-point bifurcations, which leads to the formation of strongly heterogeneous activity in the inhibitory population. This phenomenon occurs when we increase the strength of the synaptic weights in the inhibitory population, and shows the dangers of applying mean-field hypotheses to small populations of homogeneous neurons. A reason of interest of this finding is that it shows that it is possible to obtain a heterogeneous distribution of activity across neurons, as observed in real networks, even starting from the simple assumption of neurons with homogeneous characteristics. Heterogeneity of neural responses is thought to be an important feature of neural population codes, as it helps increasing the diversity of response profiles of different neurons [14, 15] and to reduce the detrimental effect of noise correlations on information processing [16]. Both such effects help reducing the redundancy of the information carried by different neurons. It is natural to speculate that avoiding massive redundancy is particularly important for small neural systems, as they must code as much information as possible with a small number of units. Thus, the features of neural dynamics specific to small neural systems that we reported here may be helpful for their function.

The analysis we performed can be used to understand not only the dynamics of circuits at the mesoscopic scale in mammals, but also the behavior of tiny brains such as those of rotifers and nematodes [19]. However, it is important to observe that the finite-size phenomena that we described here may also in principle arise in networks of large sizes, for example when ∣*J*_*II*_∣ is at least of the order of $\frac{N}{\tau_{I}}$ in fully-connected networks, or when the connectivity is sufficiently sparse (see S1 Text). It is possible to extend our reasoning to argue that also in such cases the formation of multiple branching-point bifurcations and a stronger heterogeneity of the neural activity may arise from populations of neurons with homogeneous characteristics. This leaves the possibility open that such effects may happen also in circuits of not-so-small size.

The model we introduced is largely analytically tractable, therefore it allowed us to derive exact formulas of the local bifurcation points. We implemented these formulas in a standalone Python script, which performs the analysis of local bifurcations in a few seconds without the need of complex tools for the study of bifurcations, such as Cl_MatCont and XPPAUT, which make use of the continuation of equilibria.

To conclude, we discuss the differences and advances that our model presents with respect to previous work on neural networks, and we discuss how it can be significantly extended and generalized through the introduction of new features that improve the biological plausibility of the model, without losing the possibility to investigate analytically its dynamics.

### Comparison with previous work

#### Bifurcations in networks of arbitrary size

In his pioneer work [48], Beer provided a fairly complete description of bifurcations for 1- and 2-neuron circuits, and added a few results for networks of larger size that are valid under very specific, and not always biologically plausible, assumptions about synaptic connectivity. Our work builds on this knowledge and extends it. Specifically, we performed a detailed analytical and numerical analysis of the possible dynamical behavior of a network composed of two neural populations. Due to the high dynamical complexity of the network, we studied extensively the case of an excitatory population composed of an arbitrary number of neurons *N*_*E*_ and an inhibitor populations with *N*_*I*_ = 2, and then we described how to extend our results to the case *N*_*I*_ > 2.

The neurons in each population were identical, and this made the equations of our model symmetric under permutations of the neural indexes within a population. More precisely, our equations are invariant under the action of the group *S*_*N*_*E*__ × *S*_*N*_*I*__, where *S*_*N*_*α*__ is the permutation group on *N*_*α*_ items (also known as *symmetric group*). To the best of our knowledge, previous studies of homogeneous neural systems with symmetry have been restricted to networks of neural oscillators composed of one population with symmetry group *S*_*N*_ [78]. Networks composed of one excitatory and one inhibitory population have been considered (e.g. [13, 66]), but only for large populations or in the thermodynamic limit *N* → ∞. In our work we performed a detailed bifurcation analysis for a two-populations network in the more complex case of populations with arbitrary finite size. The presence in our finite-size model of a second population (and the corresponding *S*_*N*_*E*__ × *S*_*N*_*I*__ symmetry) increased the complexity of the model with respect to these previous studies, and was a key factor in creating a surprisingly rich network dynamics even under the hypothesis of homogeneous neurons in each population.

As in [4], we based our analytical study of bifurcations on the method introduced in [5] for local codimension one bifurcations. Moreover, for the first time we extended this approach to the analysis of spontaneous symmetry-breaking and to some codimension two bifurcations. Our approach can also be easily applied to local bifurcations with codimension larger than two in neural networks composed of several populations (see S1 Text). This may become useful to study more complex and biologically plausible networks, especially when the complexity of the numerical simulations becomes prohibitive. In general we may consider a network with P populations, and whose equations are invariant under the action of the group $S_{N_{0}}\times S_{N_{1}}\cdots \times S_{N_{P-1}}$. In this network, a codimension C bifurcation is described by a (P-C)-dimensional manifold, whose parametric equations contain P-C independent parameters. The only exception, as in the case with two populations considered in this article, is the BP manifold, whose equation can be written by expressing any current as an explicit function of all the others (see Eqs (23) + (24) + (25)). For example, in a network with three populations *A*, *B*, *C*, in the codimension three diagram spanned by the currents *I*_*A*_, *I*_*B*_, *I*_*C*_ we get that:

- the bifurcations LP, H etc. are described by surfaces with two parameters;
- BT, CP etc. by lines with one parameter;
- codimension three bifurcations by points;
- the BP bifurcations are described by surfaces with explicit formulas $I_{A}=F(I_{B},I_{C})$.

However, a complete classification of bifurcations with C≥3 is still missing in the literature. Nevertheless, the method introduced in this article can be used in principle to extend the approach introduced in [5] to local bifurcations with large codimension.

#### Spontaneous symmetry-breaking in neural fields

Since we consider identical neurons within each population, our model can be cast in the context of the bifurcation theory of dynamical systems with symmetry, which is known as *equivariant bifurcation theory* [50]. Symmetry-breaking and branching-point bifurcations have already been studied in neuroscience through equivariant bifurcation theory, but in a conceptually different way. In [51], Ermentrout and Cowan used symmetry-breaking, as well as arguments developed in the context of reaction-diffusion equations, to evaluate hallucinations in primary visual cortex under the effect of drugs. They idealized the cortex as a plane and described the local activity of a population of neurons by means of neural-field equations, so that each population was univocally identified by its position in the continuous space of the plane. Their theory exploits the symmetry the neural-field equations inherit from the geometrically regular structure of the anatomical connections in primary visual cortex. In equivariant bifurcation theory, this symmetry is described by the invariance of the equations under the action of the Euclidean group **E**(2), which is the group of rigid motions in the plane, generated by translations, rotations, and reflections. However, the network analyzed in our work is described by equations that are invariant under the action of the group *S*_*N*_*E*__ × *S*_*N*_*I*__. This is a completely different kind of symmetry compared to that of the Euclidean group **E**(2), and it allows us to study in an analytically simple way networks made of a finite number of neurons. Indeed, this is conceptually different from the theory of Ermentrout and Cowan, which relies on infinite-dimensional neural-field equations. Thus, their theory does not describe finite-size effects, and does not use the underlying symmetry for this purpose.

From a biological point of view, the strong inhibition that leads to spontaneous symmetry-breaking may loosely correspond to effect of anesthesia, as some kinds of anesthetics, such as propofol, thiopental and isoflurane, act on *γ*-Aminobutyric acid (GABA), the primary inhibitory neurotransmitter [74]. Our results prove that the dynamics repertoire of small neural populations can be completely different depending on the level of inhibition. In particular, in our model symmetry is broken by an increase of the inhibitory synaptic weights. This differs from the theory of hallucinations of Ermentrout and Cowan, in which spontaneous symmetry-breaking requires enhanced excitatory modulation as well as a decreased inhibition to occur [51].

### Future directions

#### Comparison with spiking models

Rate models of the type used in our study have been shown to generate dynamics similar to that of the firing rate in spiking neuron networks under a wide variety of conditions. These conditions include slow dynamics [80], slow synapses [81], and asynchronous states [82]. An important topic for future research is to understand when the dynamics of the rate model we used is close to that of spiking neuron models and when it is not. While addressing this issue goes far beyond the remit of the present study, it is useful to speculate on the conditions in which we expect our rate model to correspond more closely to spiking neuron models. This correspondence may depend on the details of the spiking neuron model chosen for comparison [83] as well as on the specific set of considered model parameters and the dynamics it generates [84]. In particular, it is likely that the correspondence between our model and a spiking neuron model holds better when the network is in an asynchronous state [82]. Networks with all-to-all coupling, like ours, are relatively easy to synchronize [85] since they have the shortest path length possible (*L* = 1). However, asynchrony is not incompatible with an all-to-all connectivity assumption. Asynchronous states may be generated in several ways, e.g. with both inhibitory and excitatory neurons [86] or with purely excitatory networks [83]. Because of this, it is tempting to speculate that the rate equations we used describe reasonably well the firing rate of an underlying spiking model in a wide range of bifurcation parameters *I*_*E*_, *I*_*I*_. At the light of the results of [83, 86] strong synchronization is expected to arise mainly with strongly hyperpolarizing currents *I*_*E*_, since these strong currents deactivate the excitatory population thereby effectively making the network purely inhibitory and thus more likely to strongly synchronize.

#### Possible extensions of the model

In this article, for simplicity we decided to lay down the foundations for the mathematical study of small neural systems by making a number of simplifying assumptions in the model. However, it is important to note that many of these assumptions can be relaxed in future work, while still keeping the model analytically tractable.

For example, it is possible to introduce some heterogeneity in the network. A first element of heterogeneity that we discussed in Results was the heterogeneity generated in the inhibitory population by spontaneous symmetry-breaking. However, another, and perhaps more trivial, way to introduce heterogeneity in both the neural populations is through an *explicit* symmetry-breaking, namely the breaking of the permutation symmetry by parameters in the neural Eq (3) that do not respect the symmetry. Clearly, in order to break the permutation symmetry explicitly, these parameters must introduce differences between the neurons in a given population, so that they are not identical anymore under exchange of the neural indexes. Any parameter of the network can serve to this purpose, but since in this article we focused mainly on the importance of the synaptic weights, here we suppose that the symmetry is explicitly broken by the terms *J*_*αβ*_. For this reason, we can suppose for example that the weights between two populations are independent and identically distributed as $J_{\alpha \beta }∼N({\bar{J}}_{\alpha \beta },\sigma_{J_{\alpha \beta }}^{2})$, where ${\bar{J}}_{\alpha \beta }$ and *σ*_*J*_*αβ*__ are respectively the mean and the standard deviation of the normal distribution. *σ*_*J*_*αβ*__ measures the degree of heterogeneity of the network, since for large *σ*_*J*_*αβ*__ the *N*_*α*_ × *N*_*β*_ weights from population *β* to population *α* are more likely to be different. The main effect of this heterogeneity is to remove the degeneracy of the eigenvalues *λ*_*E*,*I*_, see S11 Fig.

The spectrum of matrices with distributed weights is studied in the field of *random matrix theory* [75]. However, random asymmetric block-matrices have been considered only recently [76]. Usually these matrices are supposed to have zero-mean random entries, also on the main diagonal, while the entries of our Jacobian matrix have non-zero mean and are deterministic on the main diagonal. For this reason some heuristics must be introduced to adapt the results of random matrix theory to our neural network. For example, it is possible to prove that in the weak-inhibition regime, given sufficiently small standard deviations *σ*_*J*_*αβ*__, the eigenvalues *λ*_*E*_ and *λ*_*I*_ respectively split into *N*_*E*_−1 and *N*_*I*_−1 eigenvalues, which are uniformly distributed in the complex plane within the circles of radius $\frac{\sqrt{N_{E}}}{N}\sigma_{J_{EE}}A_{E}^{\prime }({\bar{\mu }}_{E})$ and $\frac{\sqrt{N_{I}}}{N}\sigma_{J_{II}}A_{I}^{\prime }({\bar{\mu }}_{I})$, and centered in the points with coordinates $\left(-[\frac{1}{\tau_{E}}+\frac{{\bar{J}}_{EE}}{N-1}A_{E}^{\prime }({\bar{\mu }}_{E})],0\right)$ and $\left(-[\frac{1}{\tau_{I}}+\frac{{\bar{J}}_{II}}{N-1}A_{I}^{\prime }({\bar{\mu }}_{I})],0\right)$ (see S11 Fig). Here ${\bar{\mu }}_{E,I}$ are the solutions obtained from Eq (6) after replacing *J*_*αβ*_ with ${\bar{J}}_{\alpha \beta }$. This distribution is a generalization of *Girko’s circular law* [77], and it clearly shows that heterogeneity in the neural equations removes the eigenvalues degeneracy increasing the complexity of the bifurcation structure.

It is interesting to observe that, similarly to the heterogeneity caused by spontaneous symmetry-breaking, also the one determined by explicit symmetry-breaking can be regulated dynamically through the stimuli *I*_*E*,*I*_. Indeed, the degree of heterogeneity caused by the random weights in the population *α* is $\frac{\sqrt{N_{\alpha }}}{N}\sigma_{J_{\alpha \alpha }}A_{\alpha }^{\prime }({\bar{\mu }}_{\alpha })$. Therefore, it depends on the input currents through the activation function. This suggests that in real networks the effective degree of heterogeneity is determined by a superposition of both kinds of symmetry-breaking.

Our work can also be extended to spatially organized interacting populations of neurons. As it is often the case in numerical simulations of neural-field dynamics, the populations may be arranged on a two-dimensional regular lattice with periodic boundary conditions. In topological terms, the two-dimensional lattice can be thought of as being mapped onto a torus. The torus does not have boundaries and the neurons on it would be still identical since they would all have the same number of connections to other neurons. Therefore we expect that spontaneous symmetry-breaking would play an important role also in this kind of networks.

To conclude, it is possible to study how the bifurcations affect the behavior of the network under periodic inputs, as in [57], and the relationship between bifurcations and random noise. For example, it is well-known that saddle-node bifurcations in stochastic systems lead to *critical slowing down* [12], while random fluctuations may perturb a stable equilibrium state close to an Andronov-Hopf bifurcation, generating sustained oscillations known as *quasi-cycles* [13]. Recent studies of neural models of the whole cortex at the edge of criticality have proposed stimulus- and noise-driven bifurcations as the neurophysiologic mechanism underlying the rich dynamical behavior of the brain [7, 8]. In principle our formalism may be applied to perform a systematic analysis of bifurcations in these models.

## Supporting Information

S1 Text Supplementary calculations and numerical simulations. This text contains additional information about the analytical derivation of the equilibrium points and the codimension two bifurcation diagram. By means of numerical simulations we also provide a complete picture of the codimension one bifurcation diagrams for weak and strong inhibition. Moreover, we show how the network size and sparse connections influence the branching-point bifurcations of the system.
(PDF)

S1 FigEquilibrium points obtained from the first-order perturbative expansion on the primary branch.The panels on the top represent the solutions *μ*_*E*,*I*_ for *I*_*I*_ = −10, while those at the bottom are the solutions for *I*_*I*_ = −30. The figure shows a good agreement with the numerical solutions provided by Cl_MatCont on all the portions of the primary branch with the exception of most of the cyan colored curve, and also the green and yellow ones close to point *A*, where the first-order perturbative approximation does not work anymore due to the divergence of $\mu_{E}^{\left(1\right)}$. For *I*_*I*_ = −10 the curves *μ*_*E*,*I*_ are made of 5 portions (green, yellow, blue, red, cyan), while for *I*_*I*_ = −30 the blue and red portions disappear, see text.(TIF)

S2 Fig LP curve obtained from the first-order perturbative expansion on the primary branch. The zoom in the right-hand side of the figure shows that the approximation does not describe the cusp bifurcation (CP for short), even if the overall LP curve corresponds qualitatively to that shown in Fig 7 in the main text.
(TIF)

S3 Fig Analytical bifurcation diagram obtained from the eigenvalues on the primary branch. The zoom in the right-hand side of the figure shows the CP bifurcation, which is not predicted by the first-order perturbative expansion (compare with S2 Fig).
(TIF)

S4 Fig Areas with qualitatively similar dynamics in the weak-inhibition regime. Due to the high symmetry of the codimension two bifurcation diagram shown in Fig 7 in the main text, we focus here on its upper-half part. In addition to the codimension two bifurcations presented in Fig 7, we consider three additional points p1, p2, p3 that allow us to divide the diagram horizontally in nine areas, identified by the letters A-I. The codimension one bifurcation diagram of each slice is shown in S5 Fig. For the meaning of the line colors see the main text.
(TIF)

S5 Fig Representative samples of the codimension one bifurcation diagram in the weak-inhibition regime. Each panel of this figure describes *μ*_*E*_ on the vertical axis as a function of *I*_*E*_ on the horizontal axis (the panels of *μ*_*I*_ have been omitted, see text), for different values of *I*_*I*_ in areas A-I. The stable/unstable equilibrium curves are described by plain/dashed black lines. Saddle-node (LP), as well as Andronov-Hopf (H) bifurcations, lie on the equilibrium curve. Supercritical/subcritical H bifurcations give rise to stable/unstable limit cycles, whose maxima and minima are described by plain/dashed brown curves. Homoclines, which are characterized by large amplitude limit cycles with infinite period, are described by orange loops. The dark green loops identify the values of the current at which the limit cycles cross the limit point of cycles bifurcations. Here, limit cycles change stability.
(TIF)

S6 Fig Examples of neural dynamics. This figure shows the time evolution of the excitatory membrane potentials *V*_*i*_(*t*) (for any *i* = 0,…, *N*_*E*_−1), obtained from Eq (3) of the main text for different values of the external currents. Left, we fix *I*_*I*_ = −4 (area A in S5 Fig). For *I*_*E*_ = 2 and *I*_*E*_ = 7 the solutions converge to stable foci, giving rise to damped oscillations (red and blue curve, respectively). For *I*_*E*_ = 13 and *I*_*E*_ = 15 the solutions converge to stable nodes (black and purple curve, respectively). Right, we fix *I*_*I*_ = −13.3 (area G in S5 Fig). For *I*_*E*_ = 0.216 we find low-amplitude oscillations of about 35 Hz (red curve). For *I*_*E*_ = 5.564 the amplitude of the oscillations is larger than the previous case, and the frequency increases up to 160 Hz (blue curve). High-amplitude oscillations occur for *I*_*E*_ = 11.85; since this current is close to the homoclinic bifurcation, the frequency decays to 19 Hz (black curve). Finally, for *I*_*E*_ = 12.5 the system reaches a stable node (green curve).
(TIF)

S7 Fig Areas with qualitatively similar dynamics in the strong-inhibition regime. As in S4 Fig, here we focus on the upper-half part of the codimension two bifurcation diagrams obtained for *J*_*II*_ = −34 (left), and *J*_*II*_ = −100 (right). Specifically, we identify three regions in both of them, represented by gray backgrounds, whose corresponding codimension one diagrams are shown in S8 and S9 Figs.
(TIF)

S8 Fig Representative samples of the codimension one bifurcation diagram for *J*_*II*_ = −34. Each row of this figure describes *μ*_*E*_ and *μ*_*I*_ for *J*_*II*_ = −34 as a function of *I*_*E*_, for three different values of *I*_*I*_.
(TIF)

S9 Fig Representative samples of the codimension one bifurcation diagram for *J*_*II*_ = −100. As in S8 Fig, but for stronger inhibition.
(TIF)

S10 Fig Neural dynamics at the torus bifurcation. This figure shows the time evolution of the excitatory membrane potentials at the torus bifurcation (left) and the corresponding trajectory in the phase space (right). Both the panels have been obtained for *J*_*II*_ = −100, *I*_*I*_ = −16 (area C in S7 Fig) and *I*_*E*_ ≈ 11.804. From the left panel it is easy to see that the time evolution on the torus is characterized by two (incommensurable) frequencies.
(TIF)

S11 FigRemoval of the degeneracy of the eigenvalues caused by explicit symmetry-breaking.The panels on the left column show the whole set of eigenvalues (blue dots) of the Jacobian matrix for increasing network’s size (*N* = 10, 100 and 1,000), while the panels on the right column represent a zoom of the eigenvalues close to the *x*-axis in the complex plane. The eigenvalues have been calculated numerically for ${\bar{J}}_{EE}=10$, ${\bar{J}}_{EI}=-70$, ${\bar{J}}_{IE}=70$, ${\bar{J}}_{II}=-10$, *σ*_*J*_*EE*__ = *σ*_*J*_*IE*__ = 1, *σ*_*J*_*EI*__ = *σ*_*J*_*II*__ = 0.1, and *I*_*E*_ = *I*_*I*_ = 0, while the remaining parameters have been chosen according to Table 1 in the main text. The panels on the right show that the eigenvalues *λ*_*E*_ and *λ*_*I*_ split into *N*_*E*_−1 and *N*_*I*_−1 eigenvalues respectively, which are distributed according to a circular law. The red circles represent the theoretical prediction of the support of the distribution according to random matrix theory, see text.(TIF)

S1 TableDomain of the LP parametric curve on the secondary branch.This table reports the range of the parameter $v\overset{\text{def}}{=}\mu_{I,0}$ obtained numerically from the system of inequalities (S57) in S1 Text, for different values of *J*_*II*_. We observe that for large ∣*J*_*II*_∣ the parameter $v_{\text{min}}$ reaches a constant value, while $v_{\text{max}}$ increases linearly. This result suggests that simple asymptotic expressions of $v_{\text{min}}$ and $v_{\text{max}}$ can be derived analytically. Nevertheless this calculation is beyond the purpose of the article, and is left to the interested reader.(TIF)

S1 File Python script. This code generates the codimension two bifurcation diagram of the network by using the analytical formulas derived in the article.
(PY) Click here for additional data file.
