# Supplementary material for: The Complexity of Dynamics in Small Neural Circuits
Source: PLoS Comput Biol. 2016 Aug 5;12(8):e1004992. doi: 10.1371/journal.pcbi.1004992 (PMC4975407; doi:10.1371/journal.pcbi.1004992)
Supplement: S1 Text — This text contains additional information about the analytical derivation of the equilibrium points and the codimension two bifurcation diagram. By means of numerical simulations we also provide a complete picture of the codimension one bifurcation diagrams for weak and strong inhibition. Moreover, we show how the network size and sparse connections influence the branching-point bifurcations of the system. (PDF) [file pcbi.1004992.s001.pdf]

Supporting Information

Overview of this document

The Supporting Information is organized as follows. First, in Sec. S1 we partially calculate the codimension one bifurcation diagram of the network by evaluating the equilibrium points on the primary branch. This calculation is based on an asymptotic perturbative expansion of the neural equations, therefore it provides a good description of the membrane potentials in the stationary regime only for sufficiently large values of I_E, where the branching-point bifurcations do not occur. Then, we divide the second part of the Supporting Information in two main sections, devoted to the study of weak (Sec. S2) and strong inhibition (Sec. S3) respectively. In both cases we present detailed analytical calculations of the eigenvalues of the Jacobian matrix and of the codimension two bifurcation diagram for most of the local bifurcations of the network. Since the perturbative expansion introduced in Sec. S1 cannot be extended to derive the secondary branches of the equilibrium curve, a complete picture of the codimension one bifurcation diagram cannot be derived analytically. For this reason in Sec. S4 we study the codimension one bifurcation diagrams with numerical tools. As in the main text, all the results in the Supporting Information are obtained using the parameter values shown in Table 1. To conclude, in Sec. S5 we study how important parameters of the network such as its size N and the density of the synaptic connections affect the branching-point bifurcations.

S1 Equilibrium points (codimension one bifurcation diagram)

In this section we study analytically the equilibrium points of the neural network. By inspecting the top-right panel of Fig. 2 in the main text, it is apparent that some of the solutions of the system (6) (or, equivalently, of the system (11) with $\mu_{I,0} = \ldots \mu_{I,N_I-1} \stackrel{\text{def}}{=} \mu_I$, namely on the primary branch) occur when at least one of the two nullclines is approximately constant in the phase space. For example, if we call $\mu_E = f(\mu_I)$ the explicit function obtained by solving the equation $\mathscr{F}(\mu_E, \mu_I) = 0$, two stationary solutions are obtained when $f(\cdot)$ is approximately constant in μ_I (see the vertical portions of the violet curves in the top-right panel of Fig. 2 in the main text). In turn, this means that $\mathscr{A}_I(\mu_I)$ is approximately constant in μ_I, namely the sigmoid function has saturated to 0 or $\nu_I^{\max}$. Now we consider the case of saturation to $\nu_I^{\max}$ (the case of saturation to zero will be considered briefly later in this section). In order to find the solutions of the systems (6) or (11) on the vertical portion of $f(\cdot)$, we need an asymptotic expansion of $\mathscr{A}_I(\mu_I)$ for $\mu_I \to +\infty$. If we use the algebraic activation function:

$$\mathscr{A}_\alpha(V) = \frac{\nu_\alpha^{\max}}{2}\left[1 + \frac{\frac{\Lambda_\alpha}{2}\left(V - V_\alpha^T\right)}{\sqrt{1 + \frac{\Lambda_\alpha^2}{4}\left(V - V_\alpha^T\right)^2}}\right], \tag{S1}$$

then we need an asymptotic expansion of:

$$\frac{x}{\sqrt{1+x^2}} = \frac{1}{\sqrt{1+y^2}}$$

(with $x = \frac{\Lambda_I}{2}\left(\mu_I - V_I^T\right)$ and $y = \frac{1}{x}$), about the point $y = 0^+$ (i.e. $x = +\infty$). Since:

$$\frac{1}{\sqrt{1+y^2}} = \sum_{n=0}^{\infty} (-1)^n \, \frac{(2n-1)!!}{(2n)!!} y^{2n} = 1 - \frac{1}{2}y^2 + \frac{1\cdot 3}{2\cdot 4}y^4 - \frac{1\cdot 3\cdot 5}{2\cdot 4\cdot 6}y^6 + \dots,$$

then, if we consider the expansion up to the first order, we can approximate $\mathscr{A}_I(\mu_I)$ as follows:

$$\mathscr{A}_I(\mu_I) \approx \frac{\nu_I^{\max}}{2}\left(1 + 1 - \frac{1}{2x^2}\right) = \nu_I^{\max}\left(1 - \frac{1}{4x^2}\right).$$

In a similar way, we suppose that also μ_E could be expanded in an asymptotic series, namely:

$$\mu_E = \sum_{n=0}^{\infty} \frac{\mu_E^{(n)}}{x^{2n}}.$$

In particular, here we consider only the first two terms of this expansion, since they are sufficient to describe locally the equilibrium points of the primary branch with a good approximation. Therefore $\mathscr{A}_E(\mu_E)$ can be written as follows:

$$\mathscr{A}_E(\mu_E) \approx \mathscr{A}_E\left(\mu_E^{(0)} + \frac{\mu_E^{(1)}}{x^2}\right) \approx \mathscr{A}_E\left(\mu_E^{(0)}\right) + \mathscr{A}_E'\left(\mu_E^{(0)}\right)\frac{\mu_E^{(1)}}{x^2},$$

where at the second step we have used a Taylor expansion. With these assumptions, the system (6) (or equivalently (11) on the primary branch) becomes:

$$\begin{cases} -\frac{1}{\tau_E}\left(\mu_E^{(0)} + \frac{\mu_E^{(1)}}{x^2}\right) + \frac{N_E-1}{N-1}J_{EE}\left[\mathscr{A}_E\left(\mu_E^{(0)}\right) + \mathscr{A}_E'\left(\mu_E^{(0)}\right)\frac{\mu_E^{(1)}}{x^2}\right] + \frac{N_I}{N-1}J_{EI}\nu_I^{\max}\left(1 - \frac{1}{4x^2}\right) + I_E = 0, \\ -\frac{1}{\tau_I}\left(\frac{2x}{\Lambda_I} + V_I^T\right) + \frac{N_I-1}{N-1}J_{II}\nu_I^{\max}\left(1 - \frac{1}{4x^2}\right) + \frac{N_E}{N-1}J_{IE}\left[\mathscr{A}_E\left(\mu_E^{(0)}\right) + \mathscr{A}_E'\left(\mu_E^{(0)}\right)\frac{\mu_E^{(1)}}{x^2}\right] + I_I = 0. \end{cases} \tag{S2}$$

Now in the first equation of (S2), we compare all the coefficients with the same perturbative order $\frac{1}{x^{2n}}$, obtaining:

$$-\frac{1}{\tau_E}\mu_E^{(0)} + \frac{N_E-1}{N-1}J_{EE}\mathscr{A}_E\left(\mu_E^{(0)}\right) + \frac{N_I}{N-1}J_{EI}\nu_I^{\max} + I_E = 0 \tag{S3}$$

for $n = 0$, and:

$$-\frac{1}{\tau_E}\mu_E^{(1)}+\frac{N_E-1}{N-1}J_{EE}\mathscr{A}_E'\left(\mu_E^{(0)}\right)\mu_E^{(1)}-\frac{N_I}{N-1}J_{EI}\frac{\nu_I^{\max}}{4}=0 \tag{S4}$$

for $n=1$. Remembering that $\mathscr{A}_E\left(\mu_E\right)$ is given by (S1), Eq. (S3) can be transformed into a fourth-order polynomial equation:

$$\overline{a}\left(\mu_E^{(0)}\right)^4+\overline{b}\left(\mu_E^{(0)}\right)^3+\overline{c}\left(\mu_E^{(0)}\right)^2+\overline{d}\mu_E^{(0)}+\overline{e}=0, \tag{S5}$$

where:

$$\begin{aligned}
\overline{a}=&\frac{\Lambda_E^2}{4\tau_E^2},\\
\overline{b}=&-\frac{\Lambda_E^2}{2\tau_E}\left(\overline{\phi}+\frac{V_E^T}{\tau_E}\right),\\
\overline{c}=&\frac{\Lambda_E^2}{4}\left[\overline{\phi}^2+\left(\frac{V_E^T}{\tau_E}\right)^2+\frac{4}{\tau_E}V_E^T\overline{\phi}\right]+\frac{1}{\tau_E^2}-\overline{\xi},\\
\overline{d}=&-\frac{\Lambda_E^2}{2}\overline{\phi}V_E^T\left(\frac{V_E^T}{\tau_E}+\overline{\phi}\right)-\frac{2}{\tau_E}\overline{\phi}+2\overline{\xi}V_E^T,\\
\overline{e}=&\left(\frac{\Lambda_E}{2}\overline{\phi}V_E^T\right)^2+\overline{\phi}^2-\overline{\xi}\left(V_E^T\right)^2,\\
\overline{\phi}=&\frac{N_E-1}{N-1}J_{EE}\frac{\nu_E^{\max}}{2}+\frac{N_I}{N-1}J_{EI}\nu_I^{\max}+I_E,\\
\overline{\xi}=&\left(\frac{N_E-1}{N-1}J_{EE}\frac{\nu_E^{\max}\Lambda_E}{4}\right)^2.
\end{aligned} \tag{S6}$$

The solutions of Eq. (S5) are:

$$\begin{aligned}
\left[\mu_E^{(0)}\right]_{0,1}=&-\frac{\overline{b}}{4\overline{a}}-\overline{Z}\pm\frac{1}{2}\sqrt{-4\overline{Z}^2-2\overline{p}+\frac{\overline{q}}{\overline{Z}}},\\
\left[\mu_E^{(0)}\right]_{2,3}=&-\frac{\overline{b}}{4\overline{a}}+\overline{Z}\pm\frac{1}{2}\sqrt{-4\overline{Z}^2-2\overline{p}-\frac{\overline{q}}{\overline{Z}}},
\end{aligned} \tag{S7}$$

where:

$$
\begin{aligned}
\overline{p} =& \frac{8\overline{ac} - 3\overline{b}^2}{8\overline{a}^2}, \\
\overline{q} =& \frac{\overline{b}^3 - 4\overline{abc} + 8\overline{a}^2\overline{d}}{8\overline{a}^3}, \\
\overline{Z} =& \frac{1}{2}\sqrt{-\frac{2}{3}\overline{p} + \frac{1}{3\overline{a}}\left(\overline{Q} + \frac{\overline{\Delta}_0}{\overline{Q}}\right)}, \\
\overline{Q} =& \sqrt[3]{\frac{\overline{\Delta}_1 + \sqrt{\overline{\Delta}_1^2 - 4\overline{\Delta}_0^3}}{2}}, \\
\overline{\Delta}_0 =& \overline{c}^2 - 3\overline{bd} + 12\overline{ae}, \\
\overline{\Delta}_1 =& 2\overline{c}^3 - 9\overline{bcd} + 27\overline{b}^2\overline{e} + 27\overline{ad}^2 - 72\overline{ace}.
\end{aligned}
\tag{S8}
$$

Some solutions $\mu_E^{(0)}$ may be complex and must be discarded, since we want to describe stationary membrane potentials which are real quantities. From Eq. (S4) we obtain:

$$
\mu_E^{(1)} = \frac{\frac{N_I}{N-1} J_{EI} \frac{\nu_I^{\max}}{4}}{-\frac{1}{\tau_E} + \frac{N_E - 1}{N-1} J_{EE} \mathscr{A}_E' \left(\mu_E^{(0)}\right)}. \tag{S9}
$$

The second equation of (S2) contains a term proportional to x. For this reason it cannot be solved as the first equation of (S2) (namely by comparing all the terms with the same order $\frac{1}{x^{2n}}$), therefore we need to solve it directly without further simplifications. We observe that it can be transformed into the following third-order polynomial equation:

$$
\widetilde{a}x^3 + \widetilde{b}x^2 + \widetilde{c}x + \widetilde{d} = 0,
$$

where:

$$
\begin{aligned}
\widetilde{a} =& \frac{2}{\Lambda_I \tau_I}, \\
\widetilde{b} =& -\left(\frac{N_I-1}{N-1} J_{II} \nu_I^{\max} + \frac{N_E}{N-1} J_{IE} \mathscr{A}_E\left(\mu_E^{(0)}\right) + I_I - \frac{V_I^T}{\tau_I}\right), \\
\widetilde{c} =& 0, \\
\widetilde{d} =& \frac{N_I-1}{N-1} J_{II} \frac{\nu_I^{\max}}{4} - \frac{N_E}{N-1} J_{IE} \mathscr{A}_E'\left(\mu_E^{(0)}\right) \mu_E^{(1)}.
\end{aligned} \tag{S10}
$$

Its solutions are:

$$
x_k = -\frac{1}{3\widetilde{a}}\left(\widetilde{b} + u_k \widetilde{Q} + \frac{\widetilde{\Delta}_0}{u_k \widetilde{Q}}\right) \tag{S11}
$$

for $k = 0, 1, 2$, where:

$$
u_0 = 1, \qquad u_1 = \tfrac{-1+\iota\sqrt{3}}{2}, \qquad u_2 = \tfrac{-1-\iota\sqrt{3}}{2},
$$

$$
\begin{aligned}
\widetilde{Q} =& \sqrt[3]{\frac{\widetilde{\Delta}_1 + \sqrt{\widetilde{\Delta}_1^2 - 4\widetilde{\Delta}_0^3}}{2}}, \\
\widetilde{\Delta}_0 =& \widetilde{b}^2, \\
\widetilde{\Delta}_1 =& 2\widetilde{b}^3 + 27\widetilde{a}^2\widetilde{d},
\end{aligned}
$$

and $\iota = \sqrt{-1}$. According to the De Moivre's formula, for every u_k there are different solutions of the square and cube roots that define $\widetilde{Q}$. Different combinations of $\mu_E^{(0)}$, u_k and De Moivre's solutions provide equivalent results for x. Therefore after removing all the redundant and complex solutions, we obtain only 3 or 5 possible formulas for $\widetilde{Q}$, depending on the strength of I_I.

We start by analyzing the case with weak inhibitory current, e.g. $I_I = -10$. If we choose for example u_2, we get 5 solutions:

$$
\widetilde{Q}_{\text{green}} = \tfrac{1}{2}\sqrt[3]{\tfrac{\left|\widetilde{\Delta}_1 + \sqrt{\widetilde{\Delta}_1^2 - 4\widetilde{\Delta}_0^3}\right|}{2}}\left(1 - \iota\sqrt{3}\right), \qquad \mu_E^{(0)} = \left[\mu_E^{(0)}\right]_2, \qquad \widetilde{\Delta}_1^2 - 4\widetilde{\Delta}_0^3 \geq 0,
$$

$$
\left.
\begin{aligned}
\widetilde{Q}_{\text{yellow, blue}} =& \sqrt{\widetilde{\Delta}_0}\left(\cos\vartheta - \iota\sin\vartheta\right), \qquad \mu_E^{(0)} = \left[\mu_E^{(0)}\right]_{3,0} \\
\widetilde{Q}_{\text{red, cyan}} =& \sqrt{\widetilde{\Delta}_0}\left(\cos\vartheta + \iota\sin\vartheta\right), \qquad \mu_E^{(0)} = \left[\mu_E^{(0)}\right]_{0,3}
\end{aligned}
\right\} \widetilde{\Delta}_0 \geq 0,\ \vartheta = \frac{1}{3}\text{atan2}\left(\sqrt{4\widetilde{\Delta}_0^3 - \widetilde{\Delta}_1^2}, \widetilde{\Delta}_1\right),\ 4\widetilde{\Delta}_0^3 - \widetilde{\Delta}_1^2 \geq 0,
$$

where the colors refer to those of the top panels in S1 Fig. Thus from Eq. (S11) we obtain:

$$\begin{aligned}
x_{\text{green}} &= -\tfrac{1}{3\widetilde{a}}\left(\widetilde{b}-\sqrt[3]{\tfrac{\left|\widetilde{\Delta}_1+\sqrt{\widetilde{\Delta}_1^2-4\widetilde{\Delta}_0^3}\right|}{2}}-\frac{\widetilde{b}^2}{\sqrt[3]{\tfrac{\left|\widetilde{\Delta}_1+\sqrt{\widetilde{\Delta}_1^2-4\widetilde{\Delta}_0^3}\right|}{2}}}\right), \qquad \mu_E^{(0)}=\left[\mu_E^{(0)}\right]_2 \,, \\
x_{\text{yellow, blue}} &= -\tfrac{\widetilde{b}}{3\widetilde{a}}\left(1+\sqrt{3}\sin\vartheta+\cos\vartheta\right), \qquad \mu_E^{(0)}=\left[\mu_E^{(0)}\right]_{3,0} \,, \qquad \text{(S12)} \\
x_{\text{red, cyan}} &= -\tfrac{\widetilde{b}}{3\widetilde{a}}\left(1-\sqrt{3}\sin\vartheta+\cos\vartheta\right), \qquad \mu_E^{(0)}=\left[\mu_E^{(0)}\right]_{0,3} \,.
\end{aligned}$$

Finally, by replacing Eqs. (S7) + (S9) + (S12) into $\mu_E = \mu_E^{(0)} + \frac{\mu_E^{(1)}}{x^2}$ and $\mu_I = \frac{2x}{\Lambda_I} + V_I^T$, we obtain the stationary solutions of the membrane potentials in the two populations, as shown in the top panels of S1 Fig. In this figure we observe a good agreement between the analytical formulas and the numerical solutions provided by Cl_MatCont, on all the portions of the curve, with the exception of most of the cyan colored one, and also the green and yellow curves close to point A.

Now we compute the coordinates of the points A, B, C, D that define the ranges of the 5 colored portions of the primary branch. The most interesting ones are the points A, C since there the system undergoes a saddle-node bifurcation (LP for short). At point A, the zeroth-order approximation is very precise (i.e. $\mu_E(A) \approx \mu_E^{(0)}(A)$) since $\mu_I(A) \gg 1$. Thus we can use this approximation to find the coordinates of this bifurcation point. If we derive Eq. (S3) with respect to $\mu_E^{(0)}$, we obtain:

$$\frac{dI_E}{d\mu_E^{(0)}} = \frac{1}{\tau_E} - \frac{N_E-1}{N-1} J_{EE} \mathscr{A}_E'\left(\mu_E^{(0)}\right), \qquad \text{(S13)}$$

where, according to Eq. (S1):

$$\mathscr{A}_\alpha'(V) = \frac{\nu_\alpha^{\max}\Lambda_\alpha}{4} \frac{1}{\sqrt{\left[1+\frac{\Lambda_\alpha^2}{4}\left(V-V_\alpha^T\right)^2\right]^3}}. \qquad \text{(S14)}$$

Since $\frac{dI_E}{d\mu_E^{(0)}} = 0$ at point A, we obtain:

$$\mu_E^{(0)}(A) = V_E^T + \frac{2}{\Lambda_E}\sqrt{\sqrt[3]{\left(\frac{N_E-1}{N-1} J_{EE} \frac{\nu_E^{\max}\Lambda_E}{4}\tau_E\right)^2} - 1}. \qquad \text{(S15)}$$

We discard the solution with the $-\sqrt{\ }$, because it is quantitatively different from the numerical value of $\mu_E(A)$ provided by Cl_MatCont (its meaning will be clarified later in this section). Now, if we replace Eq. (S15) into Eq. (S3), we get:

$$I_E\left(A\right)=\frac{1}{\tau_E}\mu_E^{(0)}\left(A\right)-\frac{N_E-1}{N-1}J_{EE}\mathscr{A}_E\left(\mu_E^{(0)}\left(A\right)\right)-\frac{N_I}{N-1}J_{EI}\nu_I^{\max}. \tag{S16}$$

From Eq. (S13) we see that the first-order correction does not work around A, because the right-hand side of this formula is equal to zero in A. This term is also the denominator of $\mu_E^{(1)}$ (see Eq. (S9)), which explains why in A the first-order correction cannot be applied. Nevertheless, here the zeroth-order approximation (S15) + (S16) is in very good agreement with the numerical calculation of the equilibrium point (the reason will be clarified in SubSec. S2.2.1).

To conclude, from the second equation of (6) or (11), we get:

$$-\frac{1}{\tau_I}\mu_I\left(A\right)+\frac{N_I-1}{N-1}J_{II}\mathscr{A}_I\left(\mu_I\left(A\right)\right)+\frac{N_E}{N-1}J_{IE}\mathscr{A}_E\left(\mu_E^{(0)}\left(A\right)\right)+I_I=0. \tag{S17}$$

Since $\mu_I\left(A\right)\gg 1$ and therefore $\mathscr{A}_I\left(\mu_I\left(A\right)\right)\approx\nu_I^{\max}$, we obtain:

$$\mu_I\left(A\right)\approx\tau_I\left(\frac{N_I-1}{N-1}J_{II}\nu_I^{\max}+\frac{N_E}{N-1}J_{IE}\mathscr{A}_E\left(\mu_E^{(0)}\left(A\right)\right)+I_I\right).$$

A more precise way to calculate $\mu_I\left(A\right)$ is to solve directly Eq. (S17), which can be transformed into the following fourth-order polynomial equation:

$$\breve{a}\mu_I^4\left(A\right)+\breve{b}\mu_I^3\left(A\right)+\breve{c}\mu_I^2\left(A\right)+\breve{d}\mu_I\left(A\right)+\breve{e}=0, \tag{S18}$$

where:

$$
\begin{aligned}
\breve{a} =& \frac{\Lambda_I^2}{4\tau_I^2}, \\
\breve{b} =& -\frac{\Lambda_I^2}{2\tau_I}\left(\breve{\phi}+\frac{V_I^T}{\tau_I}\right), \\
\breve{c} =& \frac{\Lambda_I^2}{4}\left[\breve{\phi}^2+\left(\frac{V_I^T}{\tau_I}\right)^2+\frac{4}{\tau_I}V_I^T\breve{\phi}\right]+\frac{1}{\tau_I^2}-\breve{\xi}, \\
\breve{d} =& -\frac{\Lambda_I^2}{2}\breve{\phi}V_I^T\left(\frac{V_I^T}{\tau_I}+\breve{\phi}\right)-\frac{2}{\tau_I}\breve{\phi}+2\breve{\xi}V_I^T, \\
\breve{e} =& \left(\frac{\Lambda_I}{2}\breve{\phi}V_I^T\right)^2+\breve{\phi}^2-\breve{\xi}\left(V_I^T\right)^2, \\
\breve{\phi} =& \frac{N_I-1}{N-1}J_{II}\frac{\nu_I^{\max}}{2}+\frac{N_E}{N-1}J_{IE}\mathscr{A}_E\left(\mu_E^{(0)}(A)\right)+I_I, \\
\breve{\xi} =& \left(\frac{N_I-1}{N-1}J_{II}\frac{\nu_I^{\max}\Lambda_I}{4}\right)^2.
\end{aligned}
$$

The solutions of Eq. (S18) are:

$$
\begin{aligned}
\left[\mu_I(A)\right]_{0,1} &= -\frac{\breve{b}}{4\breve{a}}-\breve{Z}\pm\frac{1}{2}\sqrt{-4\breve{Z}^2-2\breve{p}+\frac{\breve{q}}{\breve{Z}}}, \\
\left[\mu_I(A)\right]_{2,3} &= -\frac{\breve{b}}{4\breve{a}}+\breve{Z}\pm\frac{1}{2}\sqrt{-4\breve{Z}^2-2\breve{p}-\frac{\breve{q}}{\breve{Z}}},
\end{aligned}
$$

where $\breve{Z}$, $\breve{p}$, $\breve{q}$ are defined similarly to Eq. (S8). It is easy to check that the solution that represents the membrane potential in A is $\left[\mu_I(A)\right]_3 = -\frac{\breve{b}}{4\breve{a}}+\breve{Z}-\frac{1}{2}\sqrt{-4\breve{Z}^2-2\breve{p}-\frac{\breve{q}}{\breve{Z}}}$, because $\left[\mu_I(A)\right]_{0,1,2}$ are quantitatively different from the numerical value of $\mu_I(A)$ provided by Cl_MatCont.

Things are more complicated for the point C. In this case we need to use the whole theory with the first-order correction, because the zeroth-order approximation is not sufficient to describe this bifurcation point. Since C is the connection point between the blue and red portions of the primary branch (see S1 Fig, top), it can be defined through the relation $x_{\mathrm{red}} = x_{\mathrm{blue}}$. Thus, from Eq. (S12) we see that this relation is equivalent to $\vartheta = 0$, namely $4\widetilde{\Delta}_0^3 - \widetilde{\Delta}_1^2 = 0$. From the expressions of $\widetilde{\Delta}_0$ and $\widetilde{\Delta}_1$ we obtain that this equation can be rewritten as $27\widetilde{a}^2\widetilde{d}\left(4\widetilde{b}^3+27\widetilde{a}^2\widetilde{d}\right)=0$. It is possible to prove that $\widetilde{d}\neq 0$ at point C, therefore the final equation that describes this bifurcation point is:

$$
4\widetilde{b}^3+27\widetilde{a}^2\widetilde{d}=0. \tag{S19}
$$

However, Eq. (S19) cannot be solved exactly, therefore we use it (together with Eqs. (S9) + (S10)) to calculate numerically $\mu_E^{(0)}(C)$, which in turn allows us to get $\mu_E^{(1)}(C)$ from Eq. (S9). Moreover, from the condition $\vartheta = 0$ we know that $x = -\frac{2\widetilde{b}}{3\widetilde{a}}$ (see Eq. (S12)), and finally we calculate $\mu_E(C)$ through $\mu_E = \mu_E^{(0)} + \frac{\mu_E^{(1)}}{x^2}$, $\mu_I(C)$ from $\mu_I = \frac{2x}{\Lambda_I} + V_I^T$, and $I_E(C)$ by means of Eq. (S3), similarly to point A (see Eq. (S16)).

Now we consider the point B. Since this is the connection point between the yellow and blue portions of the primary branch, it has to satisfy the condition $x_{\text{yellow}} = x_{\text{blue}}$, thus from Eq. (S12) we get that this is equivalent to $\left[\mu_E^{(0)}\right]_3 = \left[\mu_E^{(0)}\right]_0$. The last condition is satisfied at the inflection point of the curve $\mu_E^{(0)} = \mu_E^{(0)}(I_E)$, therefore the point B can be calculated from the equation $\frac{d^2 I_E}{d\left[\mu_E^{(0)}\right]^2} = 0$. Now, from Eq. (S13) we get:

$$\frac{d^2 I_E}{d\left[\mu_E^{(0)}\right]^2} = -\frac{N_E - 1}{N - 1} J_{EE} \mathscr{A}_E''\left(\mu_E^{(0)}\right). \tag{S20}$$

Since:

$$\mathscr{A}_\alpha''(V) = -\frac{3\nu_\alpha^{\max}\Lambda_\alpha^3}{16} \frac{V - V_\alpha^T}{\sqrt{\left[1 + \frac{\Lambda_\alpha^2}{4}\left(V - V_\alpha^T\right)^2\right]^5}}, \tag{S21}$$

we conclude that $\mu_E^{(0)}(B) = V_E^T$, or in other terms $\left[\mu_E^{(0)}\right]_3 = \left[\mu_E^{(0)}\right]_0 = V_E^T$ in B. Similarly to point C, we can use $\mu_E^{(0)}(B)$ to calculate $\mu_E^{(1)}(B)$ and x (we can use x_{yellow} or equivalently x_{blue}), from which we get $\mu_E(B)$, $\mu_I(B)$ and $I_E(B)$. The same idea can be applied to calculate the coordinates of the point D, since again $x_{\text{red}} = x_{\text{cyan}}$ when $\left[\mu_E^{(0)}\right]_0 = \left[\mu_E^{(0)}\right]_3$. The only difference with point B is that now the variable x must be calculated through the formula of x_{red} or that of x_{cyan}. We also get $I_E(D) = I_E(B) = \frac{1}{\tau_E}V_E^T - \frac{N_E - 1}{N-1}J_{EE}\frac{\nu_E^{\max}}{2} - \frac{N_I}{N-1}J_{EI}\nu_I^{\max}$, which is in agreement with S1 Fig.

On the other side, when I_I is sufficiently large and negative, e.g. $I_I = -30$, the term $\widetilde{\Delta}_1^2 - 4\widetilde{\Delta}_0^3$ can be zero on the yellow and cyan portions for some $I_E < I_E(B)$. At this point $\vartheta = 0$, therefore according to Eq. (S12), we get $x_{\text{yellow}} = x_{\text{cyan}}$, or in other terms the two portions meet each other. In this way the point C is formed by the yellow and cyan portions, while the blue and red ones disappear from the primary branch (see S1 Fig, bottom).

In general we observe that the divergence of the perturbative expansion on the cyan portion of the primary branch around point A prevents the use of our approximation for studying the secondary branches that emanate from the branching points. This is due to the fact that these branches generally extend to $I_E < I_E(A)$ (see Figs. 8 and 9 in the main text), where the perturbative approximation cannot be used anymore, because the membrane potential μ_I is not large enough to saturate the activation function. We also observe that, according to the numerical analysis performed in the main text, for $I_E \ll I_E(A)$ we can have other LP bifurcations. As we said at the beginning of this section, the term $\mathscr{A}_I(\mu_I)$ in Eqs. (6) or (11) saturates to 0 or $\nu_I^{\max}$. Up to now we have considered the case $\mathscr{A}_I(\mu_I) \approx \nu_I^{\max}$, while the remaining LP bifurcations are obtained when the activation function saturates to zero. In this case we need to use the following asymptotic expansion for $\mu_I \to -\infty$:

$$\mathscr{A}_I(\mu_I) \approx \frac{\nu_I^{\max}}{4x^2}. \tag{S22}$$

We do not show the explicit calculation of the equilibrium points, which is left to the interested readers.

Our approximation can be further improved if we consider higher-order corrections in the perturbative expansion. At the second order we get $\mu_E = \mu_E^{(0)} + \frac{\mu_E^{(1)}}{x^2} + \frac{\mu_E^{(2)}}{x^4}$, which means that when $\mathscr{A}_I(\mu_I)$ saturates for example to $\nu_I^{\max}$, we need to use the asymptotic expansion $\mathscr{A}(\mu_I) \approx \nu_I^{\max}\left(1 - \frac{1}{4x^2} + \frac{3}{16x^4}\right)$ for $\mu_I \to +\infty$. In this case, the variable x satisfies a fifth-order polynomial equation, which can be solved analytically in terms of complicated Jacobi theta functions [1]. However also in this case the formulas of $\mu_{E,I}$ diverge for $I_E \to I_E(A)$, due to explosion of both the terms $\mu_E^{(1)}$ and $\mu_E^{(2)}$. This justifies the use of the first-order approximation developed in this section, by virtue of its lower complexity.

S2 Weak-inhibition regime

In this section we perform an analytical study of the bifurcations in the weak-inhibition regime. In more detail, in SubSec. S2.1 we find the analytical formulas of the eigenvalues of the neural network, which will be used to derive its analytical codimension two bifurcation diagram, as shown in SubSec. S2.2.

S2.1 Eigenvalues

As we reported in the main text, for weak inhibition (i.e. $\psi < 1$) the membrane potentials are always homogeneous in each population, therefore it is easy to verify that in this case the Jacobian matrix $\mathcal{J}$ of the network at the equilibrium points is:

$$\mathcal{J} = \begin{bmatrix} \mathscr{J}_{EE} & \mathscr{J}_{EI} \\ \mathscr{J}_{IE} & \mathscr{J}_{II} \end{bmatrix}, \qquad \mathscr{J}_{\alpha\beta} = \begin{cases} -\frac{1}{\tau_\alpha}\mathrm{Id}_{N_\alpha} + \frac{J_{\alpha\alpha}}{N-1}\mathscr{A}'_\alpha(\mu_\alpha)\left(\mathbb{I}_{N_\alpha} - \mathrm{Id}_{N_\alpha}\right), & \text{for } \alpha = \beta, \\ \frac{J_{\alpha\beta}}{N-1}\mathscr{A}'_\beta(\mu_\beta)\,\mathbb{I}_{N_\alpha,N_\beta}, & \text{for } \alpha \neq \beta, \end{cases} \tag{S23}$$

where μ_α are the solutions of Eq. (6) in the main text, $\mathbb{I}_{N_\alpha,N_\beta}$ is the $N_\alpha \times N_\beta$ all-ones matrix (with $\mathbb{I}_{N_\alpha} \overset{\text{def}}{=} \mathbb{I}_{N_\alpha,N_\alpha}$), and Id_{N_α} is the $N_\alpha \times N_\alpha$ identity matrix. The characteristic equation of the network is $\det(\mathcal{J} - \lambda\mathrm{Id}_N) = 0$, where λ are the eigenvalues of the Jacobian matrix. In other terms, the equation that we need to solve to calculate the eigenvalues is:

$$\det\left(\begin{bmatrix} \mathscr{J}_{EE} - \lambda\mathrm{Id}_{N_E} & \mathscr{J}_{EI} \\ \mathscr{J}_{IE} & \mathscr{J}_{II} - \lambda\mathrm{Id}_{N_I} \end{bmatrix}\right) = 0. \tag{S24}$$

We can evaluate this determinant by means of the following formulas for block matrices:

$$\det\left(\begin{bmatrix} \mathfrak{A} & \mathfrak{B} \\ \mathfrak{C} & \mathfrak{D} \end{bmatrix}\right) = \begin{cases} \det(\mathfrak{A})\det\left(\mathfrak{D} - \mathfrak{C}\mathfrak{A}^{-1}\mathfrak{B}\right), \text{ if } \det(\mathfrak{A}) \neq 0, \\ \det(\mathfrak{D})\det\left(\mathfrak{A} - \mathfrak{B}\mathfrak{D}^{-1}\mathfrak{C}\right), \text{ if } \det(\mathfrak{D}) \neq 0, \end{cases} \tag{S25}$$

where in our case $\mathfrak{A} = \mathscr{J}_{EE} - \lambda \mathrm{Id}_{N_E}$, $\mathfrak{B} = \mathscr{J}_{EI}$, $\mathfrak{C} = \mathscr{J}_{IE}$, $\mathfrak{D} = \mathscr{J}_{II} - \lambda \mathrm{Id}_{N_I}$.

We start by calculating the eigenvalues λ_i of $\mathcal{J}$ such that $\det\left(\mathscr{J}_{II} - \lambda_i \mathrm{Id}_{N_I}\right) \neq 0$ for some values of the index i. In other words, we find the eigenvalues of $\mathcal{J}$ which are not also eigenvalues of $\mathscr{J}_{II}$, if they exist. Therefore we have to assume that $\lambda_i \neq \lambda_j^{II}$ for $j = 0, \ldots, N_I - 1$, where λ_j^{II} are the eigenvalues of the circulant matrix $\mathscr{J}_{II}$:

$$\lambda_0^{II} = -\frac{1}{\tau_I} + \frac{N_I - 1}{N - 1} J_{II} \mathscr{A}_I'(\mu_I),$$

$$\lambda_j^{II} = -\left[\tfrac{1}{\tau_I} + \tfrac{J_{II}}{N-1} \mathscr{A}_I'(\mu_I)\right], \qquad j = 1, \ldots, N_I - 1.$$

By using the second formula in Eq. (S25), we get that Eq. (S24) implies:

$$\det\left(\mathfrak{A} - \mathfrak{B}\mathfrak{D}^{-1}\mathfrak{C}\right) = 0. \tag{S26}$$

Now, since $\mathfrak{D}$ is a circulant matrix:

$$\mathfrak{D} = \mathfrak{d}_0 \mathrm{Id}_{N_I} + \mathfrak{d}_1 \left(\mathbb{I}_{N_I} - \mathrm{Id}_{N_I}\right), \quad \mathfrak{d}_0 = -\frac{1}{\tau_I} - \lambda_i, \quad \mathfrak{d}_1 = \frac{J_{II}}{N-1} \mathscr{A}_I'(\mu_I),$$

then $\mathfrak{D}^{-1}$ is also circulant:

$$\mathfrak{D}^{-1} = \mathfrak{m}_0 \mathrm{Id}_{N_I} + \mathfrak{m}_1 \left(\mathbb{I}_{N_I} - \mathrm{Id}_{N_I}\right), \quad \mathfrak{m}_0 = \frac{1 - \frac{\mathfrak{d}_1 (N_I - 1)}{\mathfrak{d}_1 - \mathfrak{d}_0}}{\mathfrak{d}_0 + \mathfrak{d}_1 (N_I - 1)}, \quad \mathfrak{m}_1 = \mathfrak{m}_0 + \frac{1}{\mathfrak{d}_1 - \mathfrak{d}_0},$$

where the denominators in $\mathfrak{m}_{0,1}$ cannot be equal to zero due to the hypothesis $\lambda_i \neq \lambda_j^{II}$. Now, due to the properties of the circulant matrices, $\mathfrak{A} - \mathfrak{B}\mathfrak{D}^{-1}\mathfrak{C}$ is circulant as well:

$$\mathfrak{A} - \mathfrak{B}\mathfrak{D}^{-1}\mathfrak{C} = \mathfrak{n}_0 \mathrm{Id}_{N_E} + \mathfrak{n}_1 \left(\mathbb{I}_{N_E} - \mathrm{Id}_{N_E}\right),$$

$$\mathfrak{n}_0 = \mathfrak{a}_0 - \mathfrak{r}, \quad \mathfrak{n}_1 = \mathfrak{a}_1 - \mathfrak{r}, \quad \mathfrak{r} = N_I \mathfrak{b}_0 \mathfrak{c}_0 \left[\mathfrak{m}_0 + (N_I - 1)\, \mathfrak{m}_1\right],$$

$$\mathfrak{a}_0 = -\frac{1}{\tau_E} - \lambda_i, \quad \mathfrak{a}_1 = \frac{J_{EE}}{N-1} \mathscr{A}_E'(\mu_E), \quad \mathfrak{b}_0 = \frac{J_{EI}}{N-1} \mathscr{A}_I'(\mu_I), \quad \mathfrak{c}_0 = \frac{J_{IE}}{N-1} \mathscr{A}_E'(\mu_E).$$

Its determinant is:

$$\det\left(\mathfrak{A}-\mathfrak{B}\mathfrak{D}^{-1}\mathfrak{C}\right)=\prod_{j=0}^{N_E-1}\left(\mathfrak{n}_0+\mathfrak{n}_1\sum_{k=1}^{N_E-1}e^{\frac{2\pi jk}{N_E}\iota}\right)=\prod_{j=0}^{N_E-1}\left[\mathfrak{n}_0+\mathfrak{n}_1\left(\frac{1-e^{2\pi j\iota}}{1-e^{\frac{2\pi j}{N_E}\iota}}-1\right)\right]$$

$$=\left[\mathfrak{n}_0+\mathfrak{n}_1\left(N_E-1\right)\right]\left(\mathfrak{n}_0-\mathfrak{n}_1\right)^{N_E-1}.$$

Therefore the characteristic equation (S26) gives:

$$\mathfrak{n}_0+\mathfrak{n}_1\left(N_E-1\right)=0\qquad\text{and/or}\qquad\mathfrak{n}_0-\mathfrak{n}_1=0.\tag{S27}$$

Now we start to analyze the first equation of (S27). From it we obtain:

$$\mathfrak{a}_0-\mathfrak{r}+\left(\mathfrak{a}_1-\mathfrak{r}\right)\left(N_E-1\right)=0,$$

and therefore, by substituting the expressions of $\mathfrak{a}_{0.1}$ and $\mathfrak{r}$:

$$-\lambda_i-\mathscr{X}\left(N_I\frac{1-\frac{\mathscr{U}}{\lambda_i+\mathscr{V}}}{-\lambda_i+\mathscr{Z}}+\frac{N_I-1}{\lambda_i+\mathscr{V}}\right)+\mathscr{Y}=0,\tag{S28}$$

where:

$$\begin{aligned}&\mathscr{X}=\frac{N_EN_I}{\left(N-1\right)^2}J_{EI}J_{IE}\mathscr{A}_E'\left(\mu_E\right)\mathscr{A}_I'\left(\mu_I\right),\quad\mathscr{Y}=-\frac{1}{\tau_E}+\frac{N_E-1}{N-1}J_{EE}\mathscr{A}_E'\left(\mu_E\right),\\&\mathscr{Z}=-\frac{1}{\tau_I}+\frac{N_I-1}{N-1}J_{II}\mathscr{A}_I'\left(\mu_I\right),\quad\mathscr{U}=\frac{N_I-1}{N-1}J_{II}\mathscr{A}_I'\left(\mu_I\right),\quad\mathscr{V}=\frac{1}{\tau_I}+\frac{J_{II}}{N-1}\mathscr{A}_I'\left(\mu_I\right).\end{aligned}\tag{S29}$$

Now, since:

$$N_I\frac{1-\frac{\mathscr{U}}{\lambda_i+\mathscr{V}}}{-\lambda_i+\mathscr{Z}}+\frac{N_I-1}{\lambda_i+\mathscr{V}}=\frac{1}{-\lambda_i+\mathscr{Z}},$$

Eq. (S28) can be rewritten as the following polynomial equation:

$$\lambda_i^2-\left(\mathscr{Y}+\mathscr{Z}\right)\lambda_i+\left(\mathscr{Y}\mathscr{Z}-\mathscr{X}\right)=0,\tag{S30}$$

whose solutions are:

$$\lambda_{0,1}^{\mathcal{R}}=\frac{\mathcal{Y}+\mathcal{Z}\pm\sqrt{\left(\mathcal{Y}+\mathcal{Z}\right)^{2}-4\left(\mathcal{Y}\mathcal{Z}-\mathcal{X}\right)}}{2}.$$

According to the sign of the discriminant $\Delta=\left(\mathcal{Y}+\mathcal{Z}\right)^{2}-4\left(\mathcal{Y}\mathcal{Z}-\mathcal{X}\right)=\left(\mathcal{Y}-\mathcal{Z}\right)^{2}+4\mathcal{X}$, the eigenvalues $\lambda_{0,1}^{\mathcal{R}}$ can be real and distinct ($\Delta>0$), real and identical ($\Delta=0$), or complex-conjugate ($\Delta<0$).

Now we consider the second equation of (S27). From it we obtain $\mathfrak{a}_0=\mathfrak{a}_1$ and therefore:

$$\lambda_{i}=-\left[\tfrac{1}{\tau_{E}}+\tfrac{J_{EE}}{N-1}\mathscr{A}_{E}^{\prime}\left(\mu_{E}\right)\right],\qquad i=2,\ldots,N_{E}\;\;.$$

Therefore we have obtained N_E-1 identical and real eigenvalues, that for simplicity we call λ_E. However we observe that up to now we have obtained only N_E+1 eigenvalues (considering also $\lambda_{0,1}^{\mathcal{R}}$), thus some are missing.

To get the remaining eigenvalues, we can repeat the procedure above, but this time we need to calculate the eigenvalues λ_i of $\mathcal{J}$ such that $\det\left(\mathscr{J}_{EE}-\lambda_i\mathrm{Id}_{N_E}\right)\neq 0$. In this case, by using the first formula in Eq. (S25), we obtain again Eq. (S30), and also the following set of real eigenvalues:

$$\lambda_{i}=-\left[\tfrac{1}{\tau_{I}}+\tfrac{J_{II}}{N-1}\mathscr{A}_{I}^{\prime}\left(\mu_{I}\right)\right],\qquad i=N_{E}+1,\ldots,N-1\;\;,$$

that we call simply λ_I.

To sum up, we have obtained that the Jacobian matrix $\mathcal{J}$ at the equilibrium points has the following set of eigenvalues:

$$\lambda_{0,1}^{\mathcal{R}}=\frac{\mathcal{Y}+\mathcal{Z}\pm\sqrt{\left(\mathcal{Y}-\mathcal{Z}\right)^{2}+4\mathcal{X}}}{2},\quad\lambda_{E}=-\left[\frac{1}{\tau_{E}}+\frac{J_{EE}}{N-1}\mathscr{A}_{E}^{\prime}\left(\mu_{E}\right)\right],\quad\lambda_{I}=-\left[\frac{1}{\tau_{I}}+\frac{J_{II}}{N-1}\mathscr{A}_{I}^{\prime}\left(\mu_{I}\right)\right],\qquad\text{(S31)}$$

where $\lambda_{0,1}^{\mathcal{R}}$ have algebraic multiplicity 1 for $\Delta\neq 0$ or 2 for $\Delta=0$, while $\lambda_{E,I}$ have multiplicity $N_{E,I}-1$. The most fundamental quantity of our theory is the eigenvalue λ_I, since it determines the formation of the branching-point bifurcations discussed in the main text.

As we said in the main text, the eigenvalue λ_I is always negative in the weak-inhibition regime. On the other side, in the strong-inhibition regime λ_I can change sign, therefore the neural equations exhibit both homogeneous and heterogeneous solutions. This will be discussed later in Sec. S3.

S2.2 Codimension two bifurcation diagram

In this subsection we derive analytically most of the local bifurcations shown in the weak-inhibition codimension two bifurcation diagram of Fig. 7 in the main text. We cover only local bifurcations, since the global ones are analytically intractable. In particular, we find a mathematical description of the saddle-node and Andronov-Hopf curves (SubSecs. S2.2.1 and S2.2.2 respectively), as well as the coordinates of the Bogdanov-Takens points (SubSec. S2.2.3), while the cusp and generalized Hopf points are analytically intractable and therefore have to be studied by means of numerical methods. Even though in the article we focus on the case $N_I=2$, we observe that the formulas derived in this subsection are valid for arbitrary N_I.

S2.2.1 Saddle-node bifurcations on the primary branch

Here we show two different ways to calculate analytically the LP curves of the codimension two bifurcation diagram in Fig. 7 occurring on the primary branch. The first method is based on the results obtained in Sec. S1 and provides an explicit (i.e. non-parametric) but approximate formula of the LP curves. The idea is to take Eqs. (S16) and (S19), that describe the coordinates of the LP points A and C for a fixed current I_I, and to use them to obtain the corresponding curves $I_I = \mathfrak{f}(I_E)$. More explicitly, we can rewrite Eq. (S19) as $\widetilde{b} = -\sqrt[3]{\frac{27}{4}\widetilde{a}^2\widetilde{d}}$, and from the expression of $\widetilde{b}$ reported in Eq. (S10) we can isolate I_I to get:

$$I_I = \frac{V_I^T}{\tau_I} - \frac{N_I - 1}{N-1} J_{II} \nu_I^{\max} - \frac{N_E}{N-1} J_{IE} \mathscr{A}_E \left(\mu_E^{(0)}\right) + \sqrt[3]{\frac{27}{4}\widetilde{a}^2\widetilde{d}}. \tag{S32}$$

Now we observe that $\widetilde{a}$ does not depend on the external currents, while $\widetilde{d}$ depends only on I_E through $\mu_E^{(0)}$ (and therefore through the term $\overline{\phi}$, see Eqs. (S6) + (S7) + (S10)). In this way we obtain I_I as a function of I_E. According to the results shown in Sec. S1, when I_I is large enough (i.e. approximately $I_I > -20$), the point C is generated by the blue and red portions of $\mu_{E,I}$ (see S1 Fig, top), therefore in this case we need to use the expression of $\left[\mu_E^{(0)}\right]_0$ in Eq. (S32). On the contrary, for a strongly negative current (i.e. $I_I < -20$), the point C is generated by the yellow and cyan portions of the primary branch (see S1 Fig, bottom), therefore in this case we need to use $\left[\mu_E^{(0)}\right]_3$. Through this distinction we get the LP curve shown in S2 Fig, where the orange and violet portions are obtained in the two regimes of I_I that we have just described. The point F that separates the two portions occurs when $\left[\mu_E^{(0)}\right]_0 = \left[\mu_E^{(0)}\right]_3$ and therefore at $I_E = I_E(B)$ (therefore the corresponding I_I can be obtained from Eq. (S32)).

From Eq. (S7) we also observe that the orange portion of the LP curve is not defined for $-4\overline{Z}^2 - 2\overline{p} - \frac{\overline{q}}{\overline{Z}} < 0$, therefore its asymptote ($[I_E^{\mathrm{as}}]_0$, see S2 Fig) is obtained when $-4\overline{Z}^2 - 2\overline{p} - \frac{\overline{q}}{\overline{Z}} = 0$, and thus when $\left[\mu_E^{(0)}\right]_2 = \left[\mu_E^{(0)}\right]_3 = -\frac{\widetilde{b}}{4\widetilde{a}} + \overline{Z}$, according to Eq. (S7). This last condition is satisfied when the green and yellow portions of the primary branch meet each other, and therefore when $I_E = I_E(A)$. Therefore the portion of the LP curve generated by the point C converges to the portion generated by the point A, without ever touching it. In other terms:

$$[I_E^{\mathrm{as}}]_0 = I_E(A). \tag{S33}$$

This is due to the fact that Eqs. (S16) + (S32) are only an approximation of the real LP curve, since they have been derived from the perturbative expansion of the equilibrium points developed in Sec. S1. Intuitively, the divergence of I_I in the $I_E - I_I$ plane that occurs when the orange portion approaches $[I_E^{\mathrm{as}}]_0$ is due to the divergence of $\mu_E^{(1)}$, contained in $\widetilde{d}$. Since $\mu_E^{(1)}$ is the only term that may explode in Eq. (S32), we conclude that it diverges also when the curve approaches the second asymptote (i.e. $[I_E^{\mathrm{as}}]_1$, see S2 Fig). Thus from Eq. (S9) we get that also $[I_E^{\mathrm{as}}]_1$ is defined by the equation $-\frac{1}{\tau_E} + \frac{N_E-1}{N-1} J_{EE} \mathscr{A}_E' \left(\mu_E^{(0)}\right) = 0$. As we know, one solution of this equation is (S15), which defines the point A, as it must be. The second solution is that with the $-\sqrt{\ }$ that we rejected before, namely:

$$\overline{\mu}_E^{(0)} = V_E^T - \frac{2}{\Lambda_E}\sqrt{\sqrt[3]{\left(\frac{N_E - 1}{N-1} J_{EE} \frac{\nu_E^{\max}\Lambda_E}{4}\tau_E\right)^2} - 1}.$$

This formula, once substituted into Eq. (S3), provides the current of the second asymptote:

$$\left[I_E^{\mathrm{as}}\right]_1 = \frac{1}{\tau_E}\overline{\mu}_E^{(0)} - \frac{N_E-1}{N-1}J_{EE}\mathscr{A}_E\left(\overline{\mu}_E^{(0)}\right) - \frac{N_I}{N-1}J_{EI}\nu_I^{\max}. \tag{S34}$$

The second LP curve of the whole codimension two bifurcation diagram (see Fig. 7 in the main text) is obtained in the same way through the approximation (S22). Therefore we have shown that the asymptotic expansion of the equilibrium points developed in Sec. S1 is also able to describe qualitatively the LP curve in the codimension two bifurcation diagram, but for a quantitative characterization of its behavior we need another approach.

For this reason now we introduce a second method for calculating analytically the LP curve. By definition, one of the eigenvalues of the Jacobian matrix is zero at the LP points. λ_E is always negative, as well as λ_I since we are in a weak-inhibition regime. Therefore we can only have $\lambda_0^{\mathcal{R}} = 0$ or $\lambda_1^{\mathcal{R}} = 0$. If $\mathcal{Y} + \mathcal{Z} < 0$, according to Eq. (S31) the condition $\lambda_0^{\mathcal{R}} = 0$ is equivalent to $\mathcal{X} = \mathcal{Y}\mathcal{Z}$. This, according to Eq. (S29), provides:

$$\mathscr{A}_I'(\mu_I) = \frac{-\frac{1}{\tau_E\tau_I} + \frac{1}{\tau_I}\frac{N_E-1}{N-1}J_{EE}\mathscr{A}_E'(\mathfrak{v})}{-\frac{1}{\tau_E}\frac{N_I-1}{N-1}J_{II} + \frac{1}{(N-1)^2}\left[(N_E-1)(N_I-1)J_{EE}J_{II} - N_E N_I J_{EI}J_{IE}\right]\mathscr{A}_E'(\mathfrak{v})}, \tag{S35}$$

where we have defined the parameter $\mathfrak{v} \stackrel{\mathrm{def}}{=} \mu_E$. Moreover, we can invert Eq. (S35) by means of Eq. (S14), obtaining:

$$\mu_I(\mathfrak{v}) = V_I^T \pm \frac{2}{\Lambda_I}\sqrt{\sqrt[3]{\left(\frac{\nu_I^{\max}\Lambda_I}{4\mathscr{A}_I'(\mu_I)}\right)^2} - 1}, \tag{S36}$$

and from Eq. (6) of the main text we get:

$$\begin{cases} I_E(\mathfrak{v}) = \frac{1}{\tau_E}\mathfrak{v} - \frac{N_E-1}{N-1}J_{EE}\mathscr{A}_E(\mathfrak{v}) - \frac{N_I}{N-1}J_{EI}\mathscr{A}_I(\mu_I(\mathfrak{v})), \\ I_I(\mathfrak{v}) = \frac{1}{\tau_I}\mu_I(\mathfrak{v}) - \frac{N_E}{N-1}J_{IE}\mathscr{A}_E(\mathfrak{v}) - \frac{N_I-1}{N-1}J_{II}\mathscr{A}_I(\mu_I(\mathfrak{v})). \end{cases} \tag{S37}$$

Therefore Eqs. (S35) + (S36) + (S37) define a set of parametric equations with parameter $\mathfrak{v}$ for the input currents $I_{E,I}$ that generate the LP bifurcation curve. Unlike Eq. (S32), these formulas are exact, but do not provide the explicit relation between I_E and I_I.

In the same way, if $\mathcal{Y} + \mathcal{Z} > 0$, the condition $\lambda_1^{\mathcal{R}} = 0$ is equivalent again to $\mathcal{X} = \mathcal{Y}\mathcal{Z}$, therefore we re-obtain the same formulas found above. These curves have been plotted in blue in S3 Fig (the reader may easily verify the agreement with the numerical results shown in Fig. 7 in the main text), where the portion of the curve between the points BT_0 and BT_1, and that between the points BT_2 and BT_3, correspond to the condition $\lambda_1^{\mathcal{R}} = 0$, while all the other portions are generated by the condition $\lambda_0^{\mathcal{R}} = 0$. At the points $\mathrm{BT}_{0,1,2,3}$, that represent the Bogdanov-Takens bifurcations, both the eigenvalues $\lambda_{0,1}^{\mathcal{R}}$ are equal to zero, as we will see in SubSec. S2.2.3.

Every point on the LP curve has also to satisfy the following system of inequalities:

$$\begin{cases} 0 < \mathscr{A}_I'(\mu_I) \leq \frac{\nu_I^{\max}\Lambda_I}{4}, \\ (\mathcal{Y} - \mathcal{Z})^2 + 4\mathcal{X} \geq 0, \end{cases}$$

whose solution is $\mathfrak{v} \in (\mathfrak{v}_a, \mathfrak{v}_b)$, where:

$$\mathfrak{v}_{b,a} = V_E^T \pm \frac{2}{\Lambda_E}\sqrt{\sqrt[3]{\left(\frac{N_E - 1}{N - 1} J_{EE} \frac{\nu_E^{\max}\Lambda_E}{4} \tau_E\right)^2} - 1}. \tag{S38}$$

This leads to get four asymptotes $[I_E^{\mathrm{as}}]_{0,1,2,3}$ (see S3 Fig) for $\mathfrak{v} \to \mathfrak{v}_a^+$ and $\mathfrak{v} \to \mathfrak{v}_b^-$. In both the limiting cases we obtain $\mathscr{A}_I'(\mu_I) \to 0$ and therefore either $\mathscr{A}_I(\mu_I) \to 0$ or $\mathscr{A}_I(\mu_I) \to \nu_I^{\max}$. Thus the expressions of the asymptotes are:

$$\begin{aligned} [I_E^{\mathrm{as}}]_0 =& \frac{1}{\tau_E}\mathfrak{v}_b - \frac{N_E - 1}{N - 1} J_{EE}\mathscr{A}_E(\mathfrak{v}_b) - \frac{N_I}{N - 1} J_{EI}\nu_I^{\max}, \\ [I_E^{\mathrm{as}}]_1 =& \frac{1}{\tau_E}\mathfrak{v}_a - \frac{N_E - 1}{N - 1} J_{EE}\mathscr{A}_E(\mathfrak{v}_a) - \frac{N_I}{N - 1} J_{EI}\nu_I^{\max}, \\ [I_E^{\mathrm{as}}]_2 =& \frac{1}{\tau_E}\mathfrak{v}_b - \frac{N_E - 1}{N - 1} J_{EE}\mathscr{A}_E(\mathfrak{v}_b), \\ [I_E^{\mathrm{as}}]_3 =& \frac{1}{\tau_E}\mathfrak{v}_a - \frac{N_E - 1}{N - 1} J_{EE}\mathscr{A}_E(\mathfrak{v}_a). \end{aligned} \tag{S39}$$

We observe that $[I_E^{\mathrm{as}}]_{0,1}$ given by Eq. (S39) correspond to those provided by Eqs. (S33) and (S34).

To conclude, we underline that even if the cusp bifurcations are evident from S3 Fig, we cannot calculate analytically their coordinates in the codimension two bifurcation diagram. Indeed, it is possible to prove that these coordinates are given by the solutions of a high-order polynomial equation, which can be calculated only numerically.

S2.2.2 Andronov-Hopf bifurcations on the primary branch

Andronov-Hopf bifurcations (H for short) are defined by the existence of a simple pair of complex-conjugate purely imaginary eigenvalues. Since $\lambda_{E,I}$ are always real, this condition can be satisfied only by $\lambda_{0,1}^{\mathcal{R}}$, by setting $\mathcal{Y} + \mathcal{Z} = 0$ and $(\mathcal{Y} - \mathcal{Z})^2 + 4\mathcal{X} < 0$. In particular, from the equation $\mathcal{Y} + \mathcal{Z} = 0$ we get:

$$\mathscr{A}_I'(\mu_I) = \frac{N - 1}{(N_I - 1) J_{II}} \left[\frac{1}{\tau_E} + \frac{1}{\tau_I} - \frac{N_E - 1}{N - 1} J_{EE}\mathscr{A}_E'(\mathfrak{v})\right], \tag{S40}$$

where $\mathfrak{v} \stackrel{\mathrm{def}}{=} \mu_E$ as before. Following the same procedure introduced in SubSec. S2.2.1, from Eq. (S40) we obtain a set of parametric equations with parameter $\mathfrak{v}$ for the input currents $I_{E,I}$ that define the H bifurcation curve.

Every point on the curve has also to satisfy the following system of inequalities:

$$\begin{cases} 0 < \mathscr{A}_I'(\mu_I) \leq \frac{\nu_I^{\max}\Lambda_I}{4}, \\ (\mathcal{Y} - \mathcal{Z})^2 + 4\mathcal{X} < 0, \end{cases}$$

whose solution is $\mathfrak{v} \in [\mathfrak{v}_f, \mathfrak{v}_d] \cup [\mathfrak{v}_c, \mathfrak{v}_e]$, where:

$$\mathfrak{v}_{c,d} = V_E^T \pm \frac{2}{\Lambda_E}\sqrt{\sqrt[3]{\left(\frac{\nu_E^{\max}\Lambda_E (N_E - 1) J_{EE}}{4(N-1)} \frac{1}{\frac{1}{\tau_E} + \frac{1}{\tau_I} - \frac{\nu_I^{\max}\Lambda_I (N_I-1) J_{II}}{4(N-1)}}\right)^2} - 1},$$

$$\mathfrak{v}_{e,f} = V_E^T \pm \frac{2}{\Lambda_E}\sqrt{\sqrt[3]{\left(\frac{\nu_E^{\max}\Lambda_E}{4\mathfrak{z}}\right)^2} - 1},$$

$$\mathfrak{z} = \frac{-\mathfrak{b} - \sqrt{\mathfrak{b}^2 - 4\mathfrak{a}\mathfrak{c}}}{2\mathfrak{a}},$$

$$\mathfrak{a} = \left(\frac{N_E - 1}{N - 1} J_{EE}\right)^2 - \frac{N_E N_I (N_E - 1)}{(N-1)^2 (N_I - 1)} \frac{J_{EE} J_{EI} J_{IE}}{J_{II}},$$

$$\mathfrak{b} = -\frac{2}{\tau_E}\frac{N_E - 1}{N - 1} J_{EE} + \frac{N_E N_I}{(N-1)(N_I-1)} \frac{J_{EI} J_{IE}}{J_{II}} \left(\frac{1}{\tau_E} + \frac{1}{\tau_I}\right),$$

$$\mathfrak{c} = \frac{1}{\tau_E^2}.$$

These curves are represented in red in S3 Fig, and again the reader may verify the agreement with the numerical results shown in Fig. 7 in the main text. It is important to observe that in S3 Fig we did not distinguish between subcritical and supercritical H bifurcations, because the coordinates of the generalized Hopf point GH (which, by definition, divides the two kinds of bifurcations, see the main text) cannot be calculated analytically.

S2.2.3 Bogdanov-Takens bifurcations on the primary branch

Considering the results obtained in SubSec. S2.2.2, the BT bifurcations are given by $\mathfrak{v} = \mathfrak{v}_{e,f}$ (equivalently, they can be obtained from the conditions $\lambda_0^{\mathcal{R}} = \lambda_1^{\mathcal{R}} = 0$, since by definition the BTs are the contact points between the LP and H curves). Therefore we get:

$$\begin{cases} I_E\left(\mathrm{BT}_0\right)=\frac{1}{\tau_E}\mathfrak{v}_e-\frac{N_E-1}{N-1}J_{EE}\mathscr{A}_E\left(\mathfrak{v}_e\right)-\frac{N_I}{N-1}J_{EI}\mathscr{A}_I\left(\mu_I^+\right), \\ I_I\left(\mathrm{BT}_0\right)=\frac{1}{\tau_I}\mu_I^+-\frac{N_E}{N-1}J_{IE}\mathscr{A}_E\left(\mathfrak{v}_e\right)-\frac{N_I-1}{N-1}J_{II}\mathscr{A}_I\left(\mu_I^+\right), \end{cases}$$

$$\begin{cases} I_E\left(\mathrm{BT}_1\right)=\frac{1}{\tau_E}\mathfrak{v}_f-\frac{N_E-1}{N-1}J_{EE}\mathscr{A}_E\left(\mathfrak{v}_f\right)-\frac{N_I}{N-1}J_{EI}\mathscr{A}_I\left(\mu_I^+\right), \\ I_I\left(\mathrm{BT}_1\right)=\frac{1}{\tau_I}\mu_I^+-\frac{N_E}{N-1}J_{IE}\mathscr{A}_E\left(\mathfrak{v}_f\right)-\frac{N_I-1}{N-1}J_{II}\mathscr{A}_I\left(\mu_I^+\right), \end{cases}$$

$$\begin{cases} I_E\left(\mathrm{BT}_2\right)=\frac{1}{\tau_E}\mathfrak{v}_e-\frac{N_E-1}{N-1}J_{EE}\mathscr{A}_E\left(\mathfrak{v}_e\right)-\frac{N_I}{N-1}J_{EI}\mathscr{A}_I\left(\mu_I^-\right), \\ I_I\left(\mathrm{BT}_2\right)=\frac{1}{\tau_I}\mu_I^--\frac{N_E}{N-1}J_{IE}\mathscr{A}_E\left(\mathfrak{v}_e\right)-\frac{N_I-1}{N-1}J_{II}\mathscr{A}_I\left(\mu_I^-\right), \end{cases}$$

$$\begin{cases} I_E\left(\mathrm{BT}_3\right)=\frac{1}{\tau_E}\mathfrak{v}_f-\frac{N_E-1}{N-1}J_{EE}\mathscr{A}_E\left(\mathfrak{v}_f\right)-\frac{N_I}{N-1}J_{EI}\mathscr{A}_I\left(\mu_I^-\right), \\ I_I\left(\mathrm{BT}_3\right)=\frac{1}{\tau_I}\mu_I^--\frac{N_E}{N-1}J_{IE}\mathscr{A}_E\left(\mathfrak{v}_f\right)-\frac{N_I-1}{N-1}J_{II}\mathscr{A}_I\left(\mu_I^-\right), \end{cases}$$

where:

$$\mu_I^\pm=V_I^T\pm\frac{2}{\Lambda_I}\sqrt{\sqrt[3]{\left(\frac{\nu_I^{\max}\Lambda_I\left(N_I-1\right)J_{II}}{4\left(N-1\right)\left(\frac{1}{\tau_E}+\frac{1}{\tau_I}-\frac{N_E-1}{N-1}J_{EE}\mathfrak{z}\right)}\right)^2}-1}.$$

This concludes our analytical study of the local bifurcations in the weak-inhibition regime, therefore now we are ready to start the analysis of the strong-inhibition regime.

S3 Strong-inhibition regime

Here we study the case of strong inhibition, i.e. $\psi \geq 1$. This section has a similar structure to Sec. S2, therefore we start by calculating analytically the eigenvalues of the neural network (SubSec. S3.1), and then we will use them to derive analytically the codimension two bifurcation diagram (SubSec. S3.2).

S3.1 Eigenvalues

In the strong-inhibition regime the inhibitory membrane potentials can be either homogeneous or heterogeneous (see main text). In the homogeneous case, the mathematical formalism is the same of SubSec. S2.1. In the heterogeneous case, we can reinterpret the inhibitory population as a collection of smaller inhibitory subpopulations. For this reason, in order to perform our bifurcation analysis, we need to extend the results of SubSec. S2.1 to the case of a multi-population network. Therefore if we consider a network with a generic number of populations $\mathfrak{P}$ and we suppose that N_α is the size of the population α (so that $\sum_{\alpha=0}^{\mathfrak{P}-1} N_\alpha = N$), the whole connectivity matrix of the system can be written as follows:

$$J=\begin{bmatrix}\mathfrak{J}_{00} & \mathfrak{J}_{01} & \cdots & \mathfrak{J}_{0,\mathfrak{P}-1}\\ \mathfrak{J}_{10} & \mathfrak{J}_{11} & \cdots & \mathfrak{J}_{1,\mathfrak{P}-1}\\ \vdots & \vdots & \ddots & \vdots\\ \mathfrak{J}_{\mathfrak{P}-1,0} & \mathfrak{J}_{\mathfrak{P}-1,1} & \cdots & \mathfrak{J}_{\mathfrak{P}-1,\mathfrak{P}-1}\end{bmatrix}, \qquad \mathfrak{J}_{\alpha\beta}=\begin{cases}J_{\alpha\alpha}\left(\mathbb{I}_{N_\alpha}-\mathrm{Id}_{N_\alpha}\right), & \text{for } \alpha=\beta,\\ J_{\alpha\beta}\mathbb{I}_{N_\alpha,N_\beta}, & \text{for } \alpha\neq\beta,\end{cases}$$

for $\alpha, \beta = 0, \ldots, \mathfrak{P}-1$. The matrix $\mathfrak{J}_{\alpha\beta}$, which contains the connections from the population β to the population α, is $N_\alpha \times N_\beta$. Thus the Jacobian matrix of the system is:

$$\mathcal{J}=\begin{bmatrix}\mathscr{J}_{00} & \mathscr{J}_{01} & \cdots & \mathscr{J}_{0,\mathfrak{P}-1}\\ \mathscr{J}_{10} & \mathscr{J}_{11} & \cdots & \mathscr{J}_{1,\mathfrak{P}-1}\\ \vdots & \vdots & \ddots & \vdots\\ \mathscr{J}_{\mathfrak{P}-1,0} & \mathscr{J}_{\mathfrak{P}-1,1} & \cdots & \mathscr{J}_{\mathfrak{P}-1,\mathfrak{P}-1}\end{bmatrix}, \qquad \mathscr{J}_{\alpha\beta}=\begin{cases}-\frac{1}{\tau_\alpha}\mathrm{Id}_{N_\alpha}+\frac{J_{\alpha\alpha}}{M_\alpha}\mathscr{A}_\alpha'\left(\mu_\alpha\right)\left(\mathbb{I}_{N_\alpha}-\mathrm{Id}_{N_\alpha}\right), & \text{for } \alpha=\beta,\\ \frac{J_{\alpha\beta}}{M_\alpha}\mathscr{A}_\beta'\left(\mu_\beta\right)\mathbb{I}_{N_\alpha,N_\beta}, & \text{for } \alpha\neq\beta,\end{cases} \tag{S41}$$

where μ_α are the solutions of the following system of equations:

$$-\frac{1}{\tau_\alpha}\mu_\alpha+\frac{N_\alpha-1}{M_\alpha}J_{\alpha\alpha}\mathscr{A}_\alpha\left(\mu_\alpha\right)+\sum_{\substack{\beta=0\\ \beta\neq\alpha}}^{\mathfrak{P}-1}\frac{N_\beta}{M_\alpha}J_{\alpha\beta}\mathscr{A}_\beta\left(\mu_\beta\right)+I_\alpha=0, \quad \alpha=0,\ldots,\mathfrak{P}-1. \tag{S42}$$

Now we introduce the following theorem (being part of a larger work on multi-population networks, we leave it without proof, which will appear in another article).

Theorem S1 (Eigenvalues of a multi-population network) *The Jacobian matrix* (S41) *has eigenvalues:*

$$\lambda_\gamma^{\mathcal{P}}=-\left[\frac{1}{\tau_\gamma}+\frac{J_{\gamma\gamma}}{M_\gamma}\mathscr{A}_\gamma'\left(\mu_\gamma\right)\right], \tag{S43}$$

with algebraic multiplicity $N_\gamma - 1$ for $\gamma = 0, \ldots, \mathfrak{P}-1$ (in the case $\mathfrak{P} = 2$ studied in this article, these are the eigenvalues that for simplicity we called $\lambda_{E,I}$). The remaining $\mathfrak{P}$ eigenvalues are those of the following "reduced" $\mathfrak{P} \times \mathfrak{P}$ Jacobian matrix:

$$\mathcal{J}^{\mathcal{R}}=\begin{bmatrix}\mathscr{J}_{00}^{\mathcal{R}} & \mathscr{J}_{01}^{\mathcal{R}} & \cdots & \mathscr{J}_{0,\mathfrak{P}-1}^{\mathcal{R}}\\ \mathscr{J}_{10}^{\mathcal{R}} & \mathscr{J}_{11}^{\mathcal{R}} & \cdots & \mathscr{J}_{1,\mathfrak{P}-1}^{\mathcal{R}}\\ \vdots & \vdots & \ddots & \vdots\\ \mathscr{J}_{\mathfrak{P}-1,0}^{\mathcal{R}} & \mathscr{J}_{\mathfrak{P}-1,1}^{\mathcal{R}} & \cdots & \mathscr{J}_{\mathfrak{P}-1,\mathfrak{P}-1}^{\mathcal{R}}\end{bmatrix}, \qquad \mathscr{J}_{\alpha\beta}^{\mathcal{R}}=\begin{cases}-\frac{1}{\tau_\alpha}+\frac{N_\alpha-1}{M_\alpha}J_{\alpha\alpha}\mathscr{A}_\alpha'\left(\mu_\alpha\right), & \text{for } \alpha=\beta,\\ \frac{N_\beta}{M_\alpha}J_{\alpha\beta}\mathscr{A}_\beta'\left(\mu_\beta\right), & \text{for } \alpha\neq\beta.\end{cases} \tag{S44}$$

Example. The following Jacobian matrix:

$$\mathcal{J} = \begin{bmatrix} -1 & 2 & 2 & 2 & 0 & 0 & -4 & -4 & -4 \\ 2 & -1 & 2 & 2 & 0 & 0 & -4 & -4 & -4 \\ 2 & 2 & -1 & 2 & 0 & 0 & -4 & -4 & -4 \\ 2 & 2 & 2 & -1 & 0 & 0 & -4 & -4 & -4 \\ 3 & 3 & 3 & 3 & -2 & 4 & -6 & -6 & -6 \\ 3 & 3 & 3 & 3 & 4 & -2 & -6 & -6 & -6 \\ 0 & 0 & 0 & 0 & 1 & 1 & -5 & -9 & -9 \\ 0 & 0 & 0 & 0 & 1 & 1 & -9 & -5 & -9 \\ 0 & 0 & 0 & 0 & 1 & 1 & -9 & -9 & -5 \end{bmatrix}$$

represents a network with $N = 9$, $\mathfrak{P} = 3$, $N_0 = 4$, $N_1 = 2$, $N_2 = 3$, where the populations $0, 1$ are excitatory while the population 2 is inhibitory. The eigenvalues of $\mathcal{J}$ are $\lambda^{\mathcal{P}} = -3, -6, 4$, with algebraic multiplicities $3, 1, 2$ respectively. The remaining 3 eigenvalues are those of the reduced Jacobian matrix:

$$\mathcal{J}^{\mathcal{R}} = \begin{bmatrix} 5 & 0 & -16 \\ 6 & 2 & -12 \\ 0 & 3 & -23 \end{bmatrix},$$

namely:

$$\lambda_0^{\mathcal{R}} \approx -21.943, \qquad \lambda_{1,2}^{\mathcal{R}} \approx 2.971 \pm 2.564\iota.$$

As we said in the main text, for the sake of clarity in this work we study the case $N_I = 2$ (for $N_I > 2$ the analysis is still feasible, but more complicated, thus it is left to the interested reader). For $\psi \geq 1$ (and more precisely whenever $\lambda_I \geq 0$) the stationary solutions of the network are given by Eq. (12) of the main text, which can be interpreted as a special case of Eq. (S42) in the case of three populations. Therefore now the Jacobian matrix of the network is:

$$\mathcal{J} = \begin{bmatrix} \mathscr{J}_{00} & \mathscr{J}_{01} & \mathscr{J}_{02} \\ \mathscr{J}_{10} & \mathscr{J}_{11} & \mathscr{J}_{12} \\ \mathscr{J}_{20} & \mathscr{J}_{21} & \mathscr{J}_{22} \end{bmatrix}, \tag{S45}$$

where:

$$\mathscr{J}_{00} = -\frac{1}{\tau_E}\mathrm{Id}_{N_E} + \frac{J_{EE}}{N-1}\mathscr{A}_E'(\mu_E)(\mathbb{I}_{N_E} - \mathrm{Id}_{N_E}), \quad \mathscr{J}_{01} = \frac{J_{EI}}{N-1}\mathscr{A}_I'(\mu_{I,0})\mathbf{1}_{N_E}, \quad \mathscr{J}_{02} = \frac{J_{EI}}{N-1}\mathscr{A}_I'(\mu_{I,1})\mathbf{1}_{N_E},$$

$$\mathscr{J}_{10} = \frac{J_{IE}}{N-1}\mathscr{A}_E'(\mu_E)\mathbf{1}_{N_E}^t, \quad \mathscr{J}_{11} = \frac{1}{\tau_I}, \quad \mathscr{J}_{12} = \frac{J_{II}}{N-1}\mathscr{A}_I'(\mu_{I,1}),$$

$$\mathscr{J}_{20} = \frac{J_{IE}}{N-1}\mathscr{A}_E'(\mu_E)\mathbf{1}_{N_E}^t, \quad \mathscr{J}_{21} = \frac{J_{II}}{N-1}\mathscr{A}_I'(\mu_{I,0}), \quad \mathscr{J}_{22} = \frac{1}{\tau_I}.$$

Here $\mathbf{1}_{N_E}$ is the $N_E \times 1$ all-ones vector and $\mathbf{1}_{N_E}^t$ is its transpose. As we said, this allows us to reinterpret the network as if it were made of the usual excitatory population with N_E neurons, and two inhibitory populations with one neuron each. According to Thm. S1, we are in the case with $N_0 = N_E$ and $N_1 =$

$N_2 = 1$, therefore the network has the eigenvalue $\lambda_0^{\mathcal{P}} = \lambda_E = -\left[\frac{1}{\tau_E} + \frac{J_{EE}}{N-1}\mathscr{A}_E'(\mu_E)\right]$ with multiplicity $N_E - 1$ (see Eq. (S43)), while the other eigenvalues are those of the following reduced Jacobian matrix (see Eq. (S44)):

$$\mathcal{J}^{\mathcal{R}} = \begin{bmatrix} -\frac{1}{\tau_E} + \frac{N_E-1}{N-1}J_{EE}\mathscr{A}_E'(\mu_E) & \frac{J_{EI}}{N-1}\mathscr{A}_I'(\mu_{I,0}) & \frac{J_{EI}}{N-1}\mathscr{A}_I'(\mu_{I,1}) \\ \frac{N_E}{N-1}J_{IE}\mathscr{A}_E'(\mu_E) & -\frac{1}{\tau_I} & \frac{J_{II}}{N-1}\mathscr{A}_I'(\mu_{I,1}) \\ \frac{N_E}{N-1}J_{IE}\mathscr{A}_E'(\mu_E) & \frac{J_{II}}{N-1}\mathscr{A}_I'(\mu_{I,0}) & -\frac{1}{\tau_I} \end{bmatrix}. \tag{S46}$$

In other terms, the remaining eigenvalues are the solutions of the following third-order characteristic polynomial:

$$\mathring{a}\left(\lambda^{\mathcal{R}}\right)^3 + \mathring{b}\left(\lambda^{\mathcal{R}}\right)^2 + \mathring{c}\lambda^{\mathcal{R}} + \mathring{d} = 0, \tag{S47}$$

where:

$$\begin{aligned} \mathring{a} &= 1, \\ \mathring{b} &= -\operatorname{tr}\left(\mathcal{J}^{\mathcal{R}}\right), \\ \mathring{c} &= \frac{1}{2}\left\{\left[\operatorname{tr}\left(\mathcal{J}^{\mathcal{R}}\right)\right]^2 - \operatorname{tr}\left(\left(\mathcal{J}^{\mathcal{R}}\right)^2\right)\right\}, \\ \mathring{d} &= -\det\left(\mathcal{J}^{\mathcal{R}}\right). \end{aligned} \tag{S48}$$

Now, if we define:

$$\begin{aligned} \mathring{Q} &= \sqrt[3]{\frac{\mathring{\Delta}_1 + \sqrt{\mathring{\Delta}_1^2 - 4\mathring{\Delta}_0^3}}{2}}, \\ \mathring{\Delta}_0 &= \mathring{b}^2 - 3\mathring{a}\mathring{c}, \\ \mathring{\Delta}_1 &= 2\mathring{b}^3 - 9\mathring{a}\mathring{b}\mathring{c} + 27\mathring{a}^2\mathring{d}, \end{aligned}$$

we get that the eigenvalues of $\mathcal{J}^{\mathcal{R}}$ are:

$$\lambda_k^{\mathcal{R}} = -\frac{1}{3\mathring{a}}\left(\mathring{b} + u_k\mathring{Q} + \frac{\mathring{\Delta}_0}{u_k\mathring{Q}}\right),$$

for $k=0,1,2$, where as usual $u_0=1$, $u_1=\frac{-1+\iota\sqrt{3}}{2}$, $u_2=\frac{-1-\iota\sqrt{3}}{2}$.

To conclude, we observe that for arbitrary N_I there is an explicit relation between any pair of inhibitory membrane potentials $\mu_{I,i}$ and $\mu_{I,j}$, that will prove very useful in determining the parametric equations of the bifurcations. Thus from the second and third equation of the system (11) in the main text we get:

$$-\frac{1}{\tau_I}\mu_{I,i}+\frac{J_{II}}{N-1}\mathscr{A}_I\left(\mu_{I,j}\right)=-\frac{1}{\tau_I}\mu_{I,j}+\frac{J_{II}}{N-1}\mathscr{A}_I\left(\mu_{I,i}\right).$$

This equation can be rewritten for example as follows:

$$\widehat{a}\mu_{I,j}^4+\widehat{b}\mu_{I,j}^3+\widehat{c}\mu_{I,j}^2+\widehat{d}\mu_{I,j}+\widehat{e}=0,$$

where:

$$\widehat{a}=\frac{\Lambda_I^2}{4\tau_I^2},$$

$$\widehat{b}=-\frac{\Lambda_I^2}{2\tau_I}\left(\widehat{\phi}+\frac{V_I^T}{\tau_I}\right),$$

$$\widehat{c}=\frac{\Lambda_I^2}{4}\left[\widehat{\phi}^2+\left(\frac{V_I^T}{\tau_I}\right)^2+\frac{4}{\tau_I}V_I^T\widehat{\phi}\right]+\frac{1}{\tau_I^2}-\widehat{\xi},$$

$$\widehat{d}=-\frac{\Lambda_I^2}{2}\widehat{\phi}V_I^T\left(\frac{V_I^T}{\tau_I}+\widehat{\phi}\right)-\frac{2}{\tau_I}\widehat{\phi}+2\widehat{\xi}V_I^T,$$

$$\widehat{e}=\left(\frac{\Lambda_I}{2}\widehat{\phi}V_I^T\right)^2+\widehat{\phi}^2-\widehat{\xi}\left(V_I^T\right)^2,$$

$$\widehat{\phi}=\frac{1}{\tau_I}\mu_{I,i}+\frac{J_{II}}{N-1}\mathscr{A}_I\left(\mu_{I,i}\right)-\frac{\nu_I^{\max}J_{II}}{2\left(N-1\right)},$$

$$\widehat{\xi}=\left(\frac{\nu_I^{\max}\Lambda_I J_{II}}{4\left(N-1\right)}\right)^2,$$

therefore we can express $\mu_{I,j}$ in terms of $\mu_{I,i}$ by means of the formula of the solutions of a fourth-order polynomial equation:

$$\begin{aligned}\left[\mu_{I,j}\right]_{0,1}&=-\frac{\widehat{b}}{4\widehat{a}}-\widehat{Z}\pm\frac{1}{2}\sqrt{-4\widehat{Z}^2-2\widehat{p}+\frac{\widehat{q}}{\widehat{Z}}},\\ \left[\mu_{I,j}\right]_{2,3}&=-\frac{\widehat{b}}{4\widehat{a}}+\widehat{Z}\pm\frac{1}{2}\sqrt{-4\widehat{Z}^2-2\widehat{p}-\frac{\widehat{q}}{\widehat{Z}}},\end{aligned}\qquad\text{(S49)}$$

where the quantities $\widehat{Z}$, $\widehat{p}$, $\widehat{q}$ are defined similarly to Eq. (S8). The relation between $\mu_{I,i}$ and $\mu_{I,j}$ is shown in Fig. (5) of the main text. In particular, in SubSecs. (S3.2.3) and (S3.2.4) we will use Eq. (S49) to calculate analytically the LP and H bifurcations on the secondary branches in the case $N_I = 2$.

S3.2 Codimension two bifurcation diagram

Now we are ready to use all the results of SubSec. S3.1 for the evaluation of the codimension two bifurcation diagram in the strong-inhibition regime. The case $\lambda_I < 0$ is similar to SubSec. S2.2, therefore here we consider only the bifurcations that occur for $\lambda_I \geq 0$, namely under spontaneous symmetry-breaking. As usual, here we cover only local bifurcations. Of special interest is the formation of the branching points, which are discussed in SubSec. S3.2.1, since they represent the central topic of this article. Then in SubSec. S3.2.2 we calculate the zero-Hopf (neutral saddle) bifurcations, and finally we derive analytically also the saddle-node, Andronov-Hopf and Bogdanov-Takens bifurcations on the secondary branches, in SubSecs. S3.2.3, S3.2.4 and S3.2.5 respectively. The formulas that we derive for the branching-point and zero-Hopf bifurcations are valid for arbitrary N_I. However, unlike the weak-inhibition case, the formulas of the saddle-node, Andronov-Hopf and Bogdanov-Takens bifurcations are valid only for $N_I = 2$, even though our formalism can be easily extended to the case $N_I > 2$ (results not shown).

S3.2.1 Branching-point bifurcations

In this subsection we study the condition $\lambda_I = 0$ that gives rise to the branching-point bifurcations (BP for short), namely:

$$\frac{1}{\tau_I} - \frac{|J_{II}|}{N-1}\mathscr{A}_I'\left(\mu_I\left(\mathrm{BP}\right)\right) = 0. \tag{S50}$$

The solutions of this equation are:

$$\mu_I\left(\mathrm{BP}\right) = V_I^T \pm \frac{2}{\Lambda_I}\sqrt{\sqrt[3]{\psi^2} - 1}. \tag{S51}$$

Being a limiting case, the condition $\lambda_I = 0$ can be studied either from Eq. (6) or equivalently from Eq. (11) (see the main text) after setting $\mu_{I,0} = \ldots = \mu_{I,N_I-1}$. Thus for example, if we choose to use Eq. (6), from its second equation we get:

$$\mu_E\left(\mathrm{BP}\right) = V_E^T \pm \frac{2}{\Lambda_E}\sqrt{\frac{1}{\frac{1}{\left\{\frac{2(N-1)}{\nu_E^{\max} N_E J_{IE}}\left[\frac{1}{\tau_I}\mu_I(\mathrm{BP}) - \frac{N_I-1}{N-1}J_{II}\mathscr{A}_I(\mu_I(\mathrm{BP})) - I_I\right] - 1\right\}^2} - 1}} \tag{S52}$$

while from its first equation we obtain:

$$I_E = \frac{1}{\tau_E}\mu_E\left(\mathrm{BP}\right) - \frac{N_E - 1}{N-1}J_{EE}\mathscr{A}_E\left(\mu_E\left(\mathrm{BP}\right)\right) - \frac{N_I}{N-1}J_{EI}\mathscr{A}_I\left(\mu_I\left(\mathrm{BP}\right)\right), \tag{S53}$$

where $\mu_E\,(\mathrm{BP})$ and $\mu_I\,(\mathrm{BP})$ are given by Eqs. (S51) and (S52) respectively. Since $\mu_E\,(\mathrm{BP})$ depends on I_I, Eq. (S53) defines explicit functions $I_E = \mathcal{F}_\pm\,(I_I)$ that represent the currents $I_{E,I}$ at which a BP bifurcation occurs. From Eq. (S51) we observe that $\mu_I\,(\mathrm{BP})$ has two different possible values depending on the sign in front of the square root, while from Eq. (S52) we see that $\mu_E\,(\mathrm{BP})$ has four possible solutions, due to the sign of the square root and to the value of $\mu_I\,(\mathrm{BP})$. The pairs $\left(\mu_E\,(\mathrm{BP}) = V_E^T + \ldots, \mu_I^+\,(\mathrm{BP}) \overset{\mathrm{def}}{=} V_I^T + \ldots\right)$ and $\left(\mu_E\,(\mathrm{BP}) = V_E^T - \ldots, \mu_I^+\,(\mathrm{BP}) = V_I^T + \ldots\right)$ define the two portions of the curve that we call $\mathcal{F}_+$ (we observe that $\mu_E\,(\mathrm{BP})$ is calculated from the $\mu_I\,(\mathrm{BP})$ of the corresponding pair through Eq. (S52)), while the pairs $\left(\mu_E\,(\mathrm{BP}) = V_E^T + \ldots, \mu_I^-\,(\mathrm{BP}) \overset{\mathrm{def}}{=} V_I^T - \ldots\right)$ and $\left(\mu_E\,(\mathrm{BP}) = V_E^T - \ldots, \mu_I^-\,(\mathrm{BP}) = V_I^T - \ldots\right)$ define $\mathcal{F}_-$. Moreover, from Eqs. (S51) and (S52) we see that the functions $\mathcal{F}_\pm$ exist if and only if:

$$\begin{cases} \frac{\tau_I |J_{II}| \nu_I^{\max} \Lambda_I}{4(N-1)} \geq 1, \\ \frac{1}{\frac{1}{\left\{\frac{2(N-1)}{\nu_E^{\max} N_E J_{IE}}\left[\frac{1}{\tau_I}\mu_I\,(\mathrm{BP}) - \frac{N_I-1}{N-1} J_{II}\mathscr{A}_I\,(\mu_I\,(\mathrm{BP})) - I_I\right] - 1\right\}^2} - 1} \geq 0, \\ \left\{\frac{2(N-1)}{\nu_E^{\max} N_E J_{IE}}\left[\frac{1}{\tau_I}\mu_I\,(\mathrm{BP}) - \frac{N_I-1}{N-1} J_{II}\mathscr{A}_I\,(\mu_I\,(\mathrm{BP})) - I_I\right] - 1\right\}^2 \neq 1, \end{cases}$$

from which we obtain that $\mathcal{F}_+$ is defined for $[I_I^{\mathrm{as}}]_0 < I_I < [I_I^{\mathrm{as}}]_1$, while $\mathcal{F}_-$ for $[I_I^{\mathrm{as}}]_2 < I_I < [I_I^{\mathrm{as}}]_3$, where:

$$\begin{aligned} [I_I^{\mathrm{as}}]_0 &= \frac{1}{\tau_I}\mu_I^+\,(\mathrm{BP}) - \frac{N_I-1}{N-1} J_{II}\mathscr{A}_I\left(\mu_I^+\,(\mathrm{BP})\right) - \frac{\nu_E^{\max} N_E J_{IE}}{N-1}, \\ [I_I^{\mathrm{as}}]_1 &= \frac{1}{\tau_I}\mu_I^+\,(\mathrm{BP}) - \frac{N_I-1}{N-1} J_{II}\mathscr{A}_I\left(\mu_I^+\,(\mathrm{BP})\right), \\ [I_I^{\mathrm{as}}]_2 &= \frac{1}{\tau_I}\mu_I^-\,(\mathrm{BP}) - \frac{N_I-1}{N-1} J_{II}\mathscr{A}_I\left(\mu_I^-\,(\mathrm{BP})\right) - \frac{\nu_E^{\max} N_E J_{IE}}{N-1}, \\ [I_I^{\mathrm{as}}]_3 &= \frac{1}{\tau_I}\mu_I^-\,(\mathrm{BP}) - \frac{N_I-1}{N-1} J_{II}\mathscr{A}_I\left(\mu_I^-\,(\mathrm{BP})\right). \end{aligned}$$

Therefore the horizontal asymptotes $[I_I^{\mathrm{as}}]_{0,1,2,3}$ in the $I_E - I_I$ plane define the domain of the two functions $\mathcal{F}_\pm$ and describe the behavior of the BP curves for large values of $|I_E|$ (compare with Fig. 10 in the main text).

To conclude, we observe that for $N_I > 2$, we may observe the formation of special branching points that are not determined by the condition $\lambda_I = 0$, rather by the fact that one of the eigenvalues of the reduced Jacobian matrix (S44) tends to zero. These branching points can be studied analytically through our approach, but since the complexity of the corresponding eigenvalues strongly depends on N_I and on the degree of heterogeneity of the inhibitory population, we do not analyze them in detail.

S3.2.2 Zero-Hopf (neutral saddle) bifurcations

By definition, the zero-Hopf bifurcations (ZH for short) are detected when the network has a zero eigenvalue and a pair of complex-conjugate purely imaginary eigenvalues. In our case, this occurs at the intersection of the BP and H curves, therefore the ZH bifurcations satisfy the following conditions (see Eqs. (S40) and (S50)):

$$\begin{cases} \mathscr{A}_I'\left(\mu_I\left(\mathrm{ZH}\right)\right) = \frac{N-1}{\left(N_I-1\right)J_{II}}\left[\frac{1}{\tau_E}+\frac{1}{\tau_I}-\frac{N_E-1}{N-1}J_{EE}\mathscr{A}_E'\left(\mu_E\left(\mathrm{ZH}\right)\right)\right], \\ \frac{1}{\tau_I}-\frac{\left|J_{II}\right|}{N-1}\mathscr{A}_I'\left(\mu_I\left(\mathrm{ZH}\right)\right)=0. \end{cases}$$

From this system of equations we get:

$$\mathscr{A}_E'\left(\mu_E\left(\mathrm{ZH}\right)\right) = \frac{N-1}{\left(N_E-1\right)J_{EE}}\left(\frac{1}{\tau_E}+\frac{N_I}{\tau_I}\right),$$

$$\mathscr{A}_I'\left(\mu_I\left(\mathrm{ZH}\right)\right) = \frac{N-1}{\tau_I\left|J_{II}\right|},$$

and therefore:

$$\begin{aligned} \mu_E^{\pm}\left(\mathrm{ZH}\right) =& V_E^T \pm \frac{2}{\Lambda_E}\sqrt{\sqrt[3]{\left(\frac{\nu_E^{\max}\Lambda_E\left(N_E-1\right)J_{EE}}{4\left(N-1\right)\left(\frac{1}{\tau_E}+\frac{N_I}{\tau_I}\right)}\right)^2}-1}, \\ \mu_I^{\pm}\left(\mathrm{ZH}\right) =& V_I^T \pm \frac{2}{\Lambda_I}\sqrt{\sqrt[3]{\psi^2}-1}. \end{aligned} \tag{S54}$$

Therefore finally, from Eq. (6) or (11) in the main text, we see that the coordinates of the ZH bifurcations are:

$$\begin{cases} I_E\left(\mathrm{ZH}_0\right) = \frac{1}{\tau_E}\mu_E^+\left(\mathrm{ZH}\right) - \frac{N_E-1}{N-1}J_{EE}\mathscr{A}_E\left(\mu_E^+\left(\mathrm{ZH}\right)\right) - \frac{N_I}{N-1}J_{EI}\mathscr{A}_I\left(\mu_I^+\left(\mathrm{ZH}\right)\right), \\ I_I\left(\mathrm{ZH}_0\right) = \frac{1}{\tau_I}\mu_I^+\left(\mathrm{ZH}\right) - \frac{N_E}{N-1}J_{IE}\mathscr{A}_E\left(\mu_E^+\left(\mathrm{ZH}\right)\right) - \frac{N_I-1}{N-1}J_{II}\mathscr{A}_I\left(\mu_I^+\left(\mathrm{ZH}\right)\right), \end{cases}$$

$$\begin{cases} I_E\left(\mathrm{ZH}_1\right) = \frac{1}{\tau_E}\mu_E^-\left(\mathrm{ZH}\right) - \frac{N_E-1}{N-1}J_{EE}\mathscr{A}_E\left(\mu_E^-\left(\mathrm{ZH}\right)\right) - \frac{N_I}{N-1}J_{EI}\mathscr{A}_I\left(\mu_I^+\left(\mathrm{ZH}\right)\right), \\ I_I\left(\mathrm{ZH}_1\right) = \frac{1}{\tau_I}\mu_I^+\left(\mathrm{ZH}\right) - \frac{N_E}{N-1}J_{IE}\mathscr{A}_E\left(\mu_E^-\left(\mathrm{ZH}\right)\right) - \frac{N_I-1}{N-1}J_{II}\mathscr{A}_I\left(\mu_I^+\left(\mathrm{ZH}\right)\right), \end{cases}$$

$$\begin{cases} I_E\left(\mathrm{ZH}_2\right) = \frac{1}{\tau_E}\mu_E^+\left(\mathrm{ZH}\right) - \frac{N_E-1}{N-1}J_{EE}\mathscr{A}_E\left(\mu_E^+\left(\mathrm{ZH}\right)\right) - \frac{N_I}{N-1}J_{EI}\mathscr{A}_I\left(\mu_I^-\left(\mathrm{ZH}\right)\right), \\ I_I\left(\mathrm{ZH}_2\right) = \frac{1}{\tau_I}\mu_I^-\left(\mathrm{ZH}\right) - \frac{N_E}{N-1}J_{IE}\mathscr{A}_E\left(\mu_E^+\left(\mathrm{ZH}\right)\right) - \frac{N_I-1}{N-1}J_{II}\mathscr{A}_I\left(\mu_I^-\left(\mathrm{ZH}\right)\right), \end{cases}$$

$$\begin{cases} I_E\left(\mathrm{ZH}_3\right) = \frac{1}{\tau_E}\mu_E^-\left(\mathrm{ZH}\right) - \frac{N_E-1}{N-1}J_{EE}\mathscr{A}_E\left(\mu_E^-\left(\mathrm{ZH}\right)\right) - \frac{N_I}{N-1}J_{EI}\mathscr{A}_I\left(\mu_I^-\left(\mathrm{ZH}\right)\right), \\ I_I\left(\mathrm{ZH}_3\right) = \frac{1}{\tau_I}\mu_I^-\left(\mathrm{ZH}\right) - \frac{N_E}{N-1}J_{IE}\mathscr{A}_E\left(\mu_E^-\left(\mathrm{ZH}\right)\right) - \frac{N_I-1}{N-1}J_{II}\mathscr{A}_I\left(\mu_I^-\left(\mathrm{ZH}\right)\right). \end{cases}$$

In the current and the previous subsection we have studied the case $\lambda_I = 0$. From now on we will restrict ourselves to the case $\lambda_I > 0$.

S3.2.3 Saddle-node bifurcations on the secondary branches

The results of this subsection are valid only for $N_I = 2$. The LP curve that occurs on the secondary branches is given by the condition that one eigenvalue of the Jacobian matrix (S45) is equal to zero. Since the determinant of a matrix is equal to the product of its eigenvalues, and moreover the eigenvalues λ_E are never equal to zero, then the LP curve is described by the equation $\det\left(\mathcal{J}^{\mathcal{R}}\right) = 0$. After some algebra, this condition can be rewritten as $\acute{a}\mathscr{A}_E'\left(\mu_E\right) - \acute{b} = 0$, where:

$$\begin{aligned}\acute{a} =& \frac{1}{\tau_I^2}\frac{N_E-1}{N-1}J_{EE} + \frac{1}{\tau_I}\frac{N_E}{\left(N-1\right)^2}J_{EI}J_{IE}\left[\mathscr{A}_I'\left(\mu_{I,0}\right)+\mathscr{A}_I'\left(\mu_{I,1}\right)\right]\\ &+\frac{1}{\left(N-1\right)^3}\left[2N_EJ_{EI}J_{IE}J_{II}-\left(N_E-1\right)J_{EE}J_{II}^2\right]\mathscr{A}_I'\left(\mu_{I,0}\right)\mathscr{A}_I'\left(\mu_{I,1}\right),\\ \acute{b} =& \frac{1}{\tau_E}\left[\frac{1}{\tau_I^2}-\left(\frac{J_{II}}{N-1}\right)^2\mathscr{A}_I'\left(\mu_{I,0}\right)\mathscr{A}_I'\left(\mu_{I,1}\right)\right],\end{aligned} \tag{S55}$$

therefore we get:

$$\mathscr{A}_E'\left(\mu_E\right) = \frac{\acute{b}}{\acute{a}}. \tag{S56}$$

Now, by inverting Eq. (S56), we obtain μ_E as a function of $\acute{a}$, $\acute{b}$, that in turn depend on $\mu_{I,0}$ and $\mu_{I,1}$. Combining this result with Eq. (S49) (for $i = 0$ and $j = 1$), we get μ_E as a function of $\mu_{I,0}$ only, which can be used to get the parametric equations of the LP curve. Indeed, if we define $\mathfrak{v} \stackrel{\text{def}}{=} \mu_{I,0}$, now we have formulas $\mu_E^{\pm} = f_{\pm}\left(\mathfrak{v}\right)$ (depending on the sign in the expression $\mu_E^{\pm} = V_E^T \pm \cdots$) and $\mu_{I,1} = g\left(\mathfrak{v}\right)$, which replaced into Eq. (12) of the main text provide the parametric equations of the currents $I_{E,I}$ on the LP curve, as a function of the parameter $\mathfrak{v}$.

In particular, it turns out that the two portions of the LP curve are generated by $\left[\mu_{I,1}\right]_1$ (see Eq. (S49)), therefore the points on the curve have to satisfy the following system of inequalities:

$$\begin{cases}0 < \mathscr{A}_E'\left(\mu_E\right) \leq \frac{\nu_E^{\max}\Lambda_E}{4},\\ 0 < \mathscr{A}_I'\left(\mu_I\right) \leq \frac{\nu_I^{\max}\Lambda_I}{4},\\ -4\widehat{\mathcal{Z}}^2 - 2\widehat{p} + \frac{\widehat{q}}{\widehat{\mathcal{Z}}} \geq 0.\end{cases} \tag{S57}$$

The system (S57) determines the range of $\mathfrak{v}$ where the LP curve is defined, but unfortunately it cannot be solved explicitly. However, in this case the extremes of the range of $\mathfrak{v}$ do not define any important codimension two bifurcation point or asymptote, differently to what occurs for the LP curve on the primary branch. For this reason their exact evaluation is not fundamental. Nevertheless, for the sake of completeness some numerical examples are shown in S1 Table for different values of J_{II}.

From the formula of $\acute{b}$ it is interesting to observe that $\mathscr{A}_E'\left(\mu_E\right)$ is proportional to $\frac{1}{\tau_I^2} - \left(\frac{J_{II}}{N-1}\right)^2\mathscr{A}_I'\left(\mu_{I,0}\right)\mathscr{A}_I'\left(\mu_{I,1}\right)$. Since for $\lambda_I \to 0$ we get $\mu_{I,0} \to \mu_{I,1}$, and since $\frac{1}{\tau_I^2} - \left(\frac{J_{II}}{N-1}\mathscr{A}_I'\left(\mu_{I,0}\right)\right)^2 = -\lambda_I\left(\frac{1}{\tau_I} - \frac{J_{II}}{N-1}\mathscr{A}_I'\left(\mu_{I,0}\right)\right)$, we conclude that $\lambda_I = 0$ implies $\mathscr{A}_E'\left(\mu_E\right) = 0$. In other terms, according to our analytical formula the LP curve on the secondary branches cannot exist for $\lambda_I = 0$, as it must be.

To conclude, we observe that on the LP curve we have new CP bifurcations, whose coordinates cannot be calculated analytically, as in the case of the primary branch (a numerical derivation has been implemented in S1 File).

S3.2.4 Andronov-Hopf bifurcations on the secondary branches

Similarly to SubSec. S3.2.3, the results below are valid only for $N_I = 2$. If we replace $\lambda^{\mathcal{R}} = \pm\iota\omega$ into Eq. (S47), we get the following system of equations:

$$\begin{cases} \mathring{a}\omega^3 - \mathring{c}\omega = 0, \\ \mathring{b}\omega^2 - \mathring{d} = 0. \end{cases}$$

$\omega = 0$ is a solution if and only if $\mathring{d} = 0$ (this condition corresponds to the BT bifurcation that we will discuss later in SubSec. S3.2.5). Moreover, the remaining solution $\omega \neq 0$ exists if and only if $\mathring{a}\mathring{d} - \mathring{b}\mathring{c} = 0$. By means of Eq. (S48), after some algebra this last condition can be written more explicitly as $\grave{a}\left(\mathscr{A}_E'\left(\mu_E\right)\right)^2 + \grave{b}\mathscr{A}_E'\left(\mu_E\right) + \grave{c} = 0$, where:

$$\begin{aligned}
\grave{a} =& \frac{N_E - 1}{N-1} J_{EE} \left[\frac{2}{\tau_I}\frac{N_E-1}{N-1}J_{EE} + \frac{N_E}{(N-1)^2}J_{EI}J_{IE}\left(\mathscr{A}_I'\left(\mu_{I,0}\right) + \mathscr{A}_I'\left(\mu_{I,1}\right)\right)\right], \\
\grave{b} =& 2\frac{N_E}{(N-1)^3}J_{EI}J_{IE}J_{II}\mathscr{A}_I'\left(\mu_{I,0}\right)\mathscr{A}_I'\left(\mu_{I,1}\right) - \left(\frac{1}{\tau_E} + \frac{1}{\tau_I}\right)\left[\frac{4}{\tau_I}\frac{N_E-1}{N-1}J_{EE} + \frac{N_E}{(N-1)^2}J_{EI}J_{IE}\left(\mathscr{A}_I'\left(\mu_{I,0}\right) + \mathscr{A}_I'\left(\mu_{I,1}\right)\right)\right], \\
\grave{c} =& \frac{2}{\tau_I}\left[\left(\frac{1}{\tau_E} + \frac{1}{\tau_I}\right)^2 - \left(\frac{J_{II}}{N-1}\right)^2 \mathscr{A}_I'\left(\mu_{I,0}\right)\mathscr{A}_I'\left(\mu_{I,1}\right)\right].
\end{aligned} \tag{S58}$$

Therefore we get:

$$\mathscr{A}_E'\left(\mu_E^{\pm}\right) = \frac{-\grave{b} \pm \sqrt{\grave{b}^2 - 4\grave{a}\grave{c}}}{2\grave{a}}. \tag{S59}$$

Now we can follow the same procedure explained in SubSec. S3.2.3 for the LP curve, ending up with a parametric formula for the H curves on the secondary branches.

As usual, we need to find also the range of the parameter $\mathfrak{v} = \mu_{I,0}$. As for the LP curve, this range cannot be calculated analytically. However, in some cases one of the extremes of the range can be found. Indeed, in Fig. 10 of the main text we showed that when inhibition and excitation are strong enough, the H curves of the secondary branches are connected to the BP curves through the same ZH bifurcation points that we calculated in SubSec. S3.2.2, and whose inhibitory membrane potentials are known (see Eq. (S54)). First of all we want to prove briefly that the H curves of the secondary branches actually converge to the ZH points, when they exist. For $\lambda_I \to 0$ we know that $\mu_{I,0}$ and $\mu_{I,1}$ converge to the inhibitory potential μ_I of the primary branch, therefore the reduced Jacobian matrix (S46) becomes:

$$\mathcal{J}^{\mathcal{R}} \to \overline{\mathcal{J}}^{\mathcal{R}} = \begin{bmatrix} -\frac{1}{\tau_E} + \frac{N_E-1}{N-1}J_{EE}\mathscr{A}_E'\left(\mu_E\right) & \frac{J_{EI}}{N-1}\mathscr{A}_I'\left(\mu_I\right) & \frac{J_{EI}}{N-1}\mathscr{A}_I'\left(\mu_I\right) \\ \frac{N_E}{N-1}J_{IE}\mathscr{A}_E'\left(\mu_E\right) & -\frac{1}{\tau_I} & -\frac{1}{\tau_I} \\ \frac{N_E}{N-1}J_{IE}\mathscr{A}_E'\left(\mu_E\right) & -\frac{1}{\tau_I} & -\frac{1}{\tau_I} \end{bmatrix}.$$

It is easy to prove that this matrix has eigenvalues $\overline{\lambda}^{\mathcal{R}}_{0,1} = \lambda^{\mathcal{R}}_{0,1}$ and $\overline{\lambda}^{\mathcal{R}}_{2} = \lambda_I = 0$, where $\lambda^{\mathcal{R}}_{0,1}$ are given by Eq. (S31). In other words, for $\lambda_I \to 0$ the two complex-conjugate purely imaginary eigenvalues $\overline{\lambda}^{\mathcal{R}}_{0,1}$ converge to $\lambda^{\mathcal{R}}_{0,1}$. This means that the H curve of the secondary branches meets the BP curve when two eigenvalues are complex-conjugate purely imaginary and equal to those of the primary branch. As we know, the latter is the condition that defines the H curves of the primary branch. Therefore the H curves of the primary and secondary branches and the BP curve meet each other at the same point, which must be ZH, according to the results in SubSec. S3.2.2. Therefore in this case one of the extremes of the parameter for each H curve is $\mathfrak{v} = \mu_I\,(\mathrm{ZH})$, as given by Eq. (S54), while the other must be calculated numerically.

We conclude by observing that the point GH is analytically intractable, as in the case of weak inhibition.

S3.2.5 Bogdanov-Takens bifurcations on the secondary branches

The BT bifurcations occur at the intersection points between the LP and H curves, therefore they are defined by Eqs. (S56) + (S59). From them we obtain the condition:

$$\grave{a}\acute{b}^2 + \acute{a}\grave{b}\acute{b} + \acute{a}^2\grave{c} = 0. \tag{S60}$$

Now, these parameters are functions of $\mu_{I,0}$ and $\mu_{I,1}$ (see Eqs. (S55) + (S58)), therefore from Eqs. (S49) + (S60) we obtain two equations in two unknowns, from which in principle we should be able to derive the inhibitory membrane potentials. However, these equations turn out to be analytically intractable. For this reason, the BT bifurcations can be calculated only numerically (see S1 File). The simplest way is by checking when the condition $\mathring{d} = 0$ is satisfied on the H curves of the secondary branches, because this corresponds to find the points where the H and LP curves meet each other. Alternatively, it also possible to check the condition $\mathring{c} = 0$, since $\mathring{a}\mathring{d} - \mathring{b}\mathring{c} = 0$ on the H curves and $\mathring{b} \neq 0$ (with the exception of the ZH points, where $\mathring{b} = -\mathrm{tr}\left(\mathcal{J}^{\mathcal{R}}\right) = -\left(0 + \lambda^{\mathcal{R}}_0 + \lambda^{\mathcal{R}}_1\right) = 0$).

S4 Examples of dynamics from the codimension two bifurcation diagram

The codimension two bifurcation diagrams shown in Figs. 7 and 10 of the main text contain almost all the information concerning the dynamics the model is able to exhibit for different values of $I_{E,I}$ and J_{II}. However, the knowledge about the amplitude of the oscillations, as well as the value of the membrane potentials at the equilibrium points and their stability, is carried out by the codimension one diagrams. For this reason, by following the same logical path as in [2], in this section we integrate the results shown in the main text by providing the codimension one bifurcation diagrams for several values of I_I in both the weak and strong-inhibition regimes (see SubSecs. S4.1 and S4.2 respectively).

S4.1 Weak-inhibition regime

Let us first consider the weak-inhibition regime $J_{II} = -10$. Due to the high symmetry of the bifurcation diagram of the network (see Fig. 7), we focus on its upper-half part, as shown in S4 Fig. The codimension two bifurcation points, with the addition of the relative maxima and minima of the H and the limit point of cycles curves (identified by the labels p1, p2 and p3), provide a subdivision of the $I_E - I_I$ plane, which is also detectable by white and gray backgrounds. Specifically, we find nine areas, identified by the letters A-I, where the system exhibits qualitatively similar dynamics. Overall, the system displays temporal

behavior which is of considerable physiological interest. In particular, we stress the presence of damped oscillations, stable oscillations and hysteresis. Damped oscillations are involved in the thalamus, in the olfactory bulb and in the cortex [3–6] as a reaction to impulse stimulation. Moreover, stable oscillatory activity is an emerging property of the thalamocortical system and can be observed at the mesoscopic scale in EEG signals [7]. Oscillations are commonly classified in the frequency bands delta (1-4 Hz), theta (4-8 Hz), alpha (8-13 Hz), beta (13-30 Hz) and gamma (30-80 Hz). Slow and infra-slow oscillations (0.1-1 Hz and 0.02-0.1 Hz respectively) can also be observed, as well as fast and ultra-fast rhythms (80-200 Hz and 200-600 Hz) [8].

It is important to observe that our model, as described by Eq. (3) in the main text, is dimensionless. In order to match it with neurophysiological recordings, it is natural to express the membrane potentials in mV. Moreover, since in our analysis we chose $\tau_E = \tau_I = 1$ (see Table 1), and since the membrane time constant of biological neurons is of the order of 10 ms (see for example [9]), we conclude that in our simulations time is expressed in centiseconds. For this reason, if numerical simulations exhibits oscillations with dimensionless frequency f_{num}, the corresponding frequency in physical units is $100 \times f_{\text{num}}$ Hz.

By going into the details of our analysis, below we list all the areas highlighted in S4 Fig, and we describe their corresponding codimension one bifurcation diagrams. The collection of these diagrams is shown in S5 Fig, where each panel describes the equilibrium points in the excitatory population as a function of I_E, while I_I is held constant (the equilibria of the inhibitory population have been omitted due to the similarity with those of the excitatory population). In S4 Fig, we identify the bounds of each area on the I_I-axis by the notation $I_I(\mathcal{B})$, where $\mathcal{B}$ represents the bifurcation acronym; moreover, $I_I(\text{LB}) = -17$ and $I_I(\text{UB}) = -2$ characterize the lower and upper bounds, respectively, of the inhibitory current of the whole diagram.

[A-B] For $I_I(\text{CPC}) < I_I < I_I(\text{UB})$ (where CPC is the acronym for the cusp bifurcation of cycles), areas A and B are characterized by non-oscillating activity (motivated by the absence of stable limit cycles) and bistability. Nonetheless, stable equilibrium points, both foci and nodes, exist. The unstable equilibrium points and the unstable limit cycles create basin separatrixes that determine, depending on the initial conditions, the equilibrium to which the solutions converge. In particular, the lowest stable portion of the equilibrium curve is characterized by a low firing rate activity, and since the eigenvalues are complex-conjugate, these equilibria are foci. This means that for all the initial conditions belonging to the basin of attraction of the low-activity state, damped oscillations appear. On the contrary, the highest stable portion of the equilibrium curve corresponding to the high firing activity is characterized by real eigenvalues and thus is constituted by nodes. Examples of temporal evolution of the membrane potentials that converge to foci, as well as to stable nodes, are represented in S6 Fig, left. Furthermore, bistability is present in the codimension one bifurcation diagrams of areas A-B. As we said in the main text, in A bistability occurs in presence of hysteresis.

[C-H] Stable oscillations with different frequencies are present for all $I_I(\text{BT}_3) < I_I < I_I(\text{CPC})$. However, they are generated by different kinds of bifurcations. In more details, in area C the subcritical H bifurcation gives rise to unstable limit cycles. Due to the vicinity to the CPC bifurcation, the limit point of cycles curve is two-fold crossed and leads the unstable limit cycles to become stable and hereafter unstable again. Finally, these limit cycles vanish in a homoclinic bifurcation where the frequency of the oscillations decays to 0 Hz. Thus, in a narrow range of values of I_E, three stable solutions coexist: a damped oscillating solution and a non-oscillating solution, as in the areas A-B, together with stable oscillations in the range of about 30-128 Hz. Furthermore, in area D, stable limit cycles originate from the unstable ones after reaching the limit point of cycles curve. These stable limit cycles reach the maximum oscillation frequency just after they change stability; its value is about 150 Hz. The stable limit cycles vanish in the homoclinic bifurcation. Finally, in the remaining areas E-H, we always observe oscillatory activity described by one or two families of stable limit cycles. In all the cases, the frequencies span from the theoretical 0 Hz at the homoclinic bifurcation up to 170 Hz. Several examples of oscillations are

shown in S6 Fig, right.

[I] To conclude, for I_I (LB) $< I_I < I_I$ (BT_3) the system presents both stable equilibrium points and stable limit cycles. In particular, stable equilibria describe states with high firing rates for large I_E. On the contrary, states with low firing rates emerge for low I_E. Moreover, for intermediate values of I_E, the unstable equilibria are surrounded by stable limit cycles. In turn, the stable limit cycles are enclosed between two homoclinic bifurcations.

S4.2 Strong-inhibition regime

Let us now consider the strong-inhibition regimes $J_{II} = -34$ and $J_{II} = -100$. The codimension two bifurcation diagrams in these cases are more complex than that of the weak-inhibition regime. For this reason, we do not show systematically the codimension one bifurcation diagrams for each slice that can be identified in the whole codimension two diagrams, contrarily to what we did in the weak-inhibition regime. Nonetheless, we provide the codimension one diagrams in three different sample areas called A, B, C highlighted in S7 Fig, and specifically for $I_I = -4$, $I_I = -13.25$ and $I_I = -16$. We underline that, nearby the graphs of $\mu_E = \mu_E(I_E)$, we show also that of $\mu_I = \mu_I(I_E)$. This choice is motivated by the fact that, unlike the weak-inhibition regime, now the secondary branches of equilibria exhibit qualitative differences in excitatory and inhibitory neurons. The collection of these diagrams is shown in S8 Fig and S9 Fig.

[A] The model exhibits non-oscillating or oscillating activity depending on the inhibitory strength. On the one hand, for $J_{II} = -34$ the model exhibits non-oscillating activity on both the primary and the secondary branches since stable limit cycles do not exist. The primary branch is characterized by stable foci (nodes) for low (high) values of I_E. These equilibria describe low- and high-activity states, respectively. For intermediate values of I_E, the solutions of the model converge to the stable equilibria of the secondary branches. However, bistability is present. On the other hand, for $J_{II} = -100$ the H bifurcation gives rise to stable limit cycles that represent oscillating activity with frequencies from 0 Hz at the homoclinic bifurcation up to 89 Hz.

[B] For both $J_{II} = -34$ and $J_{II} = -100$, the system presents both stable equilibria (nodes and foci) and stable limit cycles. As before, stable equilibria describe states with high firing rates for large I_E, while states with low firing rates emerge for low I_E. Moreover, for intermediate values of I_E, the unstable equilibria of both the primary and secondary branches are surrounded by stable limit cycles. In particular, for $J_{II} = -34$, the high-amplitude stable oscillations produced by the H bifurcation on the primary branch exhibit frequencies from 0 Hz at the homoclinic bifurcation up to nearly 90 Hz. On the contrary, the low-amplitude oscillations originated from the secondary branches display frequencies from 124 to 154 Hz. For $J_{II} = -100$, the oscillations originated from the H bifurcation on the secondary branches span from 70 to 102 Hz.

[C] This third case turns out to be similar to the previous one. However, for both the inhibition strengths considered here, new stable limit cycles arise on the primary branch for low I_E, and vanish at the homoclinic bifurcation. In particular, for $J_{II} = -34$, their frequencies lie from 0 to 142 Hz. A similar frequency range (0-130 Hz) characterizes the limit cycles for high I_E. On the contrary, we find a narrow frequency range for the low-amplitude oscillations on the second branches (152-164 Hz). Finally, for $J_{II} = -100$, the oscillations on the primary branch exhibit frequency from 0 Hz at the homoclinic bifurcation to 73 Hz, while those on the secondary branches show frequency in the narrow range from 73 to 89 Hz. It is important to underline that, for both the inhibitory strengths, the high-frequency limit cycles display a torus bifurcation (gray loop in S8 Fig and S9 Fig). Close to this bifurcation the spectrum of the oscillations contains two incommensurable frequencies, therefore the neural network undergoes a quasiperiodic motion, as shown in S10 Fig.

S5 How the network size and sparse connections influence the branching points

It is interesting to investigate how the network parameters affect the BP bifurcations. Particularly important is to study the role of the network size N, because this allows us to compare our finite-size model with its mean-field approximation. In SubSec. S5.1 we show that the branching points disappear in the thermodynamic limit $N \to \infty$, which proves that the mean-field theory provides only an approximation of the real behavior of the network. This is a consequence of the all-to-all connectivity of the network, therefore it is natural to wonder what is the behavior of the network when its synaptic connections are sparse. In SubSec. S5.2 we argue that, contrary to the fully-connected case, the BP bifurcations may occur also in large networks if the synaptic connections are sparse enough.

S5.1 Differences between our approach and the mean-field theory

In this subsection we explain intuitively why the mean-field theory does not account for the formation of the branching points. The mean-field theory makes the assumption that within each population the membrane potentials are independent and identically distributed. Therefore, by hypothesis, it forbids the presence of heterogeneous solutions, like those that emerge from the BP bifurcations. Due to this assumption, according to the mean-field theory developed by McKean, Tanaka, Sznitman, and others [10–17], Eq. (3) in the main text can be reduced to a system of two differential equations:

$$\begin{cases} \frac{dV_E(t)}{dt} = & -\frac{1}{\tau_E} V_E(t) + R_E J_{EE} \mathbb{E}\left[\mathscr{A}_E\left(V_E(t)\right)\right] + R_I J_{EI} \mathbb{E}\left[\mathscr{A}_I\left(V_I(t)\right)\right] + I_E, \\ \frac{dV_I(t)}{dt} = & -\frac{1}{\tau_I} V_I(t) + R_E J_{IE} \mathbb{E}\left[\mathscr{A}_E\left(V_E(t)\right)\right] + R_I J_{II} \mathbb{E}\left[\mathscr{A}_I\left(V_I(t)\right)\right] + I_I, \end{cases} \tag{S61}$$

where $R_\alpha = \lim_{N\to\infty} \frac{N_\alpha}{N}$ (namely the ratio between the size of population α and that of the whole network in the thermodynamic limit, therefore in our case $R_E = 0.8$ and $R_I = 0.2$), while V_α represents any membrane potential in the population α. Moreover, $\mathbb{E}[\cdot]$ denotes the average over trials at a given time instant, and it means that the system is generally supposed to be stochastic. Stochasticity can be introduced in different ways, for example through Brownian motions, random initial conditions, or random synaptic weights [18]. In this work we consider a deterministic system, therefore we get simply $\mathbb{E}\left[\mathscr{A}_\alpha\left(V_\alpha(t)\right)\right] = \mathscr{A}_\alpha\left(V_\alpha(t)\right)$. In this way, the neural network is described by a system of two coupled equations in the unknowns $V_{E,I}(t)$, whose Jacobian matrix is:

$$\mathcal{J}^{\mathrm{mf}} = \begin{bmatrix} -\frac{1}{\tau_E} + R_E J_{EE} \mathscr{A}_E'(\mu_E) & R_I J_{EI} \mathscr{A}_I'(\mu_I) \\ R_E J_{IE} \mathscr{A}_E'(\mu_E) & -\frac{1}{\tau_I} + R_I J_{II} \mathscr{A}_I'(\mu_I) \end{bmatrix}. \tag{S62}$$

Its characteristic equation is:

$$a^{\mathrm{mf}} \left(\lambda_{0,1}^{\mathrm{mf}}\right)^2 + b^{\mathrm{mf}} \lambda_{0,1}^{\mathrm{mf}} + c^{\mathrm{mf}} = 0,$$

where:

$$a^{\mathrm{mf}} =1,$$

$$b^{\mathrm{mf}} =\frac{1}{\tau_E} + \frac{1}{\tau_I} - R_E J_{EE} \mathscr{A}_E' (\mu_E) - R_I J_{II} \mathscr{A}_I' (\mu_I),$$

$$c^{\mathrm{mf}} =\frac{1}{\tau_E \tau_I} - \left(\frac{1}{\tau_E} R_I J_{II} \mathscr{A}_I' (\mu_I) + \frac{1}{\tau_I} R_E J_{EE} \mathscr{A}_E' (\mu_E)\right) + R_E R_I (J_{EE} J_{II} - J_{IE} J_{EI}) \mathscr{A}_E' (\mu_E) \mathscr{A}_I' (\mu_I).$$

From Eq. (S31) it easy to see that $\lim_{N\to\infty} \lambda_{0,1}^{\mathcal{R}} = \lambda_{0,1}^{\mathrm{mf}}$. The only difference between $\lambda_{0,1}^{\mathcal{R}}$ and $\lambda_{0,1}^{\mathrm{mf}}$ is in the ratios that multiply the synaptic weights ($\frac{N_\alpha - 1}{N-1}$ or $\frac{N_\alpha}{N-1}$ for the finite-size network, and R_α in the mean-field case). This difference, which is due to the absence of self-connections, is small for large networks. Therefore, when compared to a finite-size network, the mean-field approximation takes into account only the information provided by $\lambda_{0,1}^{\mathcal{R}}$, and neglects that of $\lambda_{E,I}$. Clearly λ_E is always negative, therefore it never affects the changes of dynamics of the system. However, in a finite-size network λ_I can change sign, generating a BP bifurcation. The mean-field approximation neglects this information, and this is a consequence of the fact that $\lim_{N\to\infty} \lambda_I = -\frac{1}{\tau_I}$. In other words, in the thermodynamic limit the eigenvalue λ_I is always negative. For this reason, it cannot generate BP bifurcations, which is consistent with the hypothesis of identical neurons. In mathematically more rigorous terms, we get that the *center manifold* [19] of the network is not affected anymore by λ_I for $N \to \infty$, so that the dynamics is governed only by $\lambda_{0,1}^{\mathcal{R}}$. This proves that the mean-field approximation oversimplifies the description of the network, since it takes into account only the primary branch.

The LP and H bifurcations on the primary branch undergo only small quantitative changes in the thermodynamic limit, since the ratios $\frac{N_E - 1}{N-1}$ and $\frac{N_I - 1}{N-1}$ in Eqs. (16) and (20) of the main text converge quickly to 0.8 and 0.2 respectively for $N \to \infty$. Quantitative small changes occur also in the curves of the global bifurcations.

S5.2 Finite-size effects are stronger for sparse anatomical connections

According to the definition of ψ (see Eq. (4) in the main text), the formation of the branching points is determined by the ratio $\frac{|J_{II}|}{N-1}$. It is natural to argue that, more generally, in a network with sparse connections their formation is determined by the ratio $\frac{|J_{II}|}{M_I}$, where M_I is the mean number of incoming connections per neuron of the inhibitory population. For this reason, in this subsection we extend our analysis to the case of sparse connectivity matrices (to simplify matters, we consider a purely inhibitory network, since inhibition is sufficient to generate BP bifurcations). For example, we can consider the block-circulant topology $\mathcal{BC}_{F,G} (\mathcal{M}_0, \ldots, \mathcal{M}_{F-1})$ with circulant-band blocks introduced in [18]:

$$
\begin{aligned}
J = & J_{II} \begin{bmatrix} \mathfrak{B}^{(0)} & \mathfrak{B}^{(1)} & \cdots & \mathfrak{B}^{(F-1)} \\ \mathfrak{B}^{(F-1)} & \mathfrak{B}^{(0)} & \cdots & \mathfrak{B}^{(F-2)} \\ \vdots & \vdots & \ddots & \vdots \\ \mathfrak{B}^{(1)} & \mathfrak{B}^{(2)} & \cdots & \mathfrak{B}^{(0)} \end{bmatrix}, \\
\mathfrak{B}^{(i)} = & \begin{bmatrix}
1-\delta_{i0} & 1 & \cdots & 1 & 0 & \cdots & 0 & 1 & \cdots & 1 \\
1 & 1-\delta_{i0} & \ddots & & \ddots & \ddots & & \ddots & \ddots & \vdots \\
\vdots & \ddots & \ddots & \ddots & & \ddots & \ddots & & \ddots & 1 \\
1 & & \ddots & \ddots & \ddots & & \ddots & \ddots & & 0 \\
0 & \ddots & & \ddots & \ddots & \ddots & & \ddots & \ddots & \vdots \\
\vdots & & \ddots & & \ddots & \ddots & \ddots & & \ddots & 0 \\
0 & & & \ddots & & \ddots & \ddots & \ddots & & 1 \\
1 & \ddots & & & \ddots & & \ddots & \ddots & \ddots & \vdots \\
\vdots & \ddots & \ddots & & & \ddots & & \ddots & 1-\delta_{i0} & 1 \\
1 & \cdots & 1 & 0 & \cdots & 0 & 1 & \cdots & 1 & 1-\delta_{i0}
\end{bmatrix},
\end{aligned}
\tag{S63}
$$

where $\mathfrak{B}^{(0)}, ..., \mathfrak{B}^{(F-1)}$ are $G \times G$ circulant matrices (so that $FG = N$), with bandwidth $2\xi_i + 1$, for $i = 0, ..., F-1$. The network equipped with these synaptic connections can be interpreted as a collection of F neural masses with G neurons each. If we define:

$$
H(x) = \begin{cases} 0, & x \leq 0, \\ 1, & x > 0, \end{cases}
$$

then $\mathcal{M}_0 \stackrel{\text{def}}{=} 2\xi_0 - H\left(\xi_0 - \left\lfloor \frac{G}{2} \right\rfloor + (-1)^G\right)$ is the number of connections that every neuron in a given mass receives from the neurons in the same mass. Furthermore, $\mathcal{M}_i \stackrel{\text{def}}{=} 2\xi_i + 1 - H\left(\xi_i - \left\lfloor \frac{G}{2} \right\rfloor + (-1)^G\right)$, for $i = 1, ..., F-1$, is the number of connections that every neuron in the jth mass receives from the neurons in the $(i+j)$th mod F mass, for $j = 0, ..., F-1$. Therefore we conclude that $M_I = F - 1 + \sum_{i=0}^{F-1} \left[2\xi_i - H\left(\xi_i - \left\lfloor \frac{G}{2} \right\rfloor + (-1)^G\right)\right]$.

Now, if we suppose that the membrane potentials are homogeneous, the eigenvalues of the corresponding Jacobian matrix are:

$$\lambda_{mG+n} = \begin{cases} -\frac{1}{\tau_I} + \frac{J_{II}}{M_I}\left[F - 1 + \sum_{i=0}^{F-1} g\left(n, \xi_i, G\right)\right] \mathscr{A}_I'\left(\mu_I\right), & m = 0,\ \forall n, \\ -\frac{1}{\tau_I} + \frac{J_{II}}{M_I}\left[-1 + \sum_{i=0}^{F-1} e^{\frac{2\pi}{F} mi\iota} g\left(n, \xi_i, G\right)\right] \mathscr{A}_I'\left(\mu_I\right), & m \neq 0,\ \forall n, \end{cases}$$

$$g\left(n, \xi_i, G\right) = \begin{cases} 2\xi_i - H\left(\xi_i - \left\lfloor \frac{G}{2} \right\rfloor + (-1)^G\right), & n = 0,\ \forall \xi_i, \\ -1, & n \neq 0,\ \xi_i = \left\lfloor \frac{G}{2} \right\rfloor, \\ \frac{\sin\left(\frac{\pi n (2\xi_i + 1)}{G}\right)}{\sin\left(\frac{\pi n}{G}\right)} - 1, & n \neq 0,\ \xi_i < \left\lfloor \frac{G}{2} \right\rfloor, \end{cases} \tag{S64}$$

with $m = 0, ..., F - 1$ and $n = 0, ..., G - 1$. They depend on the ratio $\frac{J_{II}}{M_I}$, where now M_I does not necessarily diverge in the thermodynamic limit (consider for example the case when $G \to \infty$ for F fixed, and the parameters ξ_i are finite and independent from G). In other terms, the eigenvalues λ_{mG+n} do not necessarily converge to $-\frac{1}{\tau_I}$ for $N \to \infty$ (compare with SubSec. S5.1). Therefore this topology exhibits stronger finite-size effects than the fully-connected network, thus we expect that in the case of sparse connections the BP bifurcations may occur also in large networks.

We also underline that, according to [18], if M_I does not diverge for $N \to \infty$, the neurons do not become independent, therefore Sznitman's mean-field theory cannot be used to simplify the description of the network, even in the thermodynamic limit. This further underlines the importance of a formalism that allows the study of networks composed of an arbitrary number of neurons.

References

[1] Hermite C. Sur la résolution de l'équation du cinquieme degré. C R Acad Sci. 1858;46:508–515. Available from: http://dx.doi.org/10.1017/CBO9780511702761.003.

[2] Touboul J, Wendling F, Chauvel P, Faugeras O. Neural mass activity, bifurcations, and epilepsy. Neural Comput. 2011;23(12):3232–3286. Available from: http://dx.doi.org/10.1162/NECO_a_00206.

[3] Andersen P, Eccles J. Inhibitory phasing of neuronal discharge. Nature. 1962;17(196):645–647. Available from: http://dx.doi.org/10.1038/196645a0.

[4] Freeman WJ. Analog simulation of prepyriform cortex in the cat. Math Biosci. 1968;2:181–190. Available from: http://dx.doi.org/10.1016/0025-5564(68)90017-5.

[5] Freeman WJ. Patterns of variation in waveform of averaged evoked potentials from prepyriform cortex in cats. J Neurophysiol. 1968;31:1–13. Available from: http://jn.physiology.org/content/31/1/1.

[6] Wilson HR, Cowan JD. Excitatory and inhibitory interactions in localized populations of model neurons. Biophys J. 1972;12(1):1–24. Available from: http://dx.doi.org/10.1016/S0006-3495(72)86068-5.

[7] Steriade M, McCormick DA, Sejnowski TJ. Thalamocortical oscillations in the sleeping and aroused brain. Science. 1993;262:679–685. Available from: http://dx.doi.org/10.1126/science.8235588.

[8] Buzsáki G, Draguhn A. Neuronal oscillations in cortical networks. Science. 2004;304:1926–1929. Available from: http://dx.doi.org/10.1126/science.1099745.

[9] Diesmann M, Gewaltig MO, Aertsen A. Stable propagation of synchronous spiking in cortical neural networks. Nature. 1999;402:529–533. Available from: http://dx.doi.org/10.1038/990101.

[10] McKean H. A class of Markov processes associated with nonlinear parabolic equations. PNAS. 1966;56(6):1907–1911. Available from: http://dx.doi.org/10.1073/pnas.56.6.1907.

[11] McKean H. Propagation of chaos for a class of non-linear parabolic equations. In: Stochastic differential equations (lecture series in differential equations, session 7, Catholic Univ., 1967). Air Force Office Sci. Res., Arlington, Va.; 1967. p. 41–57.

[12] Tanaka H. Probabilistic treatment of the Boltzmann equation of Maxwellian molecules. Probab Theory Rel. 1978;46:67–105. Available from: http://dx.doi.org/10.1007/BF00535689.

[13] Tanaka H. Central limit theorem for a simple diffusion model of interacting particles. Hiroshima Math J. 1981;11(2):415–423.

[14] Tanaka H. Some probabilistic problems in the spatially homogeneous Boltzmann equation. In: Kallianpur G, editor. Theory and application of random fields. vol. 49 of Lect. Notes Contr. Inf. Sci. Springer Berlin Heidelberg; 1983. p. 258–267. Available from: http://dx.doi.org/10.1007/BFb0044698.

[15] Sznitman A. Nonlinear reflecting diffusion process, and the propagation of chaos and fluctuations associated. J Funct Anal. 1984;56:311–336. Available from: http://dx.doi.org/10.1016/0022-1236(84)90080-6.

[16] Sznitman A. A propagation of chaos result for Burgers' equation. Probab Theory Rel. 1986;71:581–613. Available from: http://dx.doi.org/10.1007/BF00699042.

[17] Sznitman A. Topics in propagation of chaos. In: Hennequin PL, editor. Ecole d'eté de probabilités de Saint-Flour XIX – 1989. vol. 1464 of Lect. Notes Math. Springer Berlin Heidelberg; 1991. p. 165–251. Available from: http://dx.doi.org/10.1007/bfb0085169.

[18] Fasoli D, Faugeras O, Panzeri S. A formalism for evaluating analytically the cross-correlation structure of a firing-rate network model. J Math Neurosci. 2015;5:6. Available from: http://dx.doi.org/10.1186/s13408-015-0020-y.

[19] Kuznetsov YA. Elements of applied bifurcation theory. vol. 112 of Appl. Math. Sci. Springer New York; 1998. Available from: http://dx.doi.org/10.1007/978-1-4757-3978-7.
