# Supplementary figures and images for: The Complexity of Dynamics in Small Neural Circuits
Source: PLoS Comput Biol. 2016 Aug 5;12(8):e1004992. doi: 10.1371/journal.pcbi.1004992 (PMC4975407; doi:10.1371/journal.pcbi.1004992)

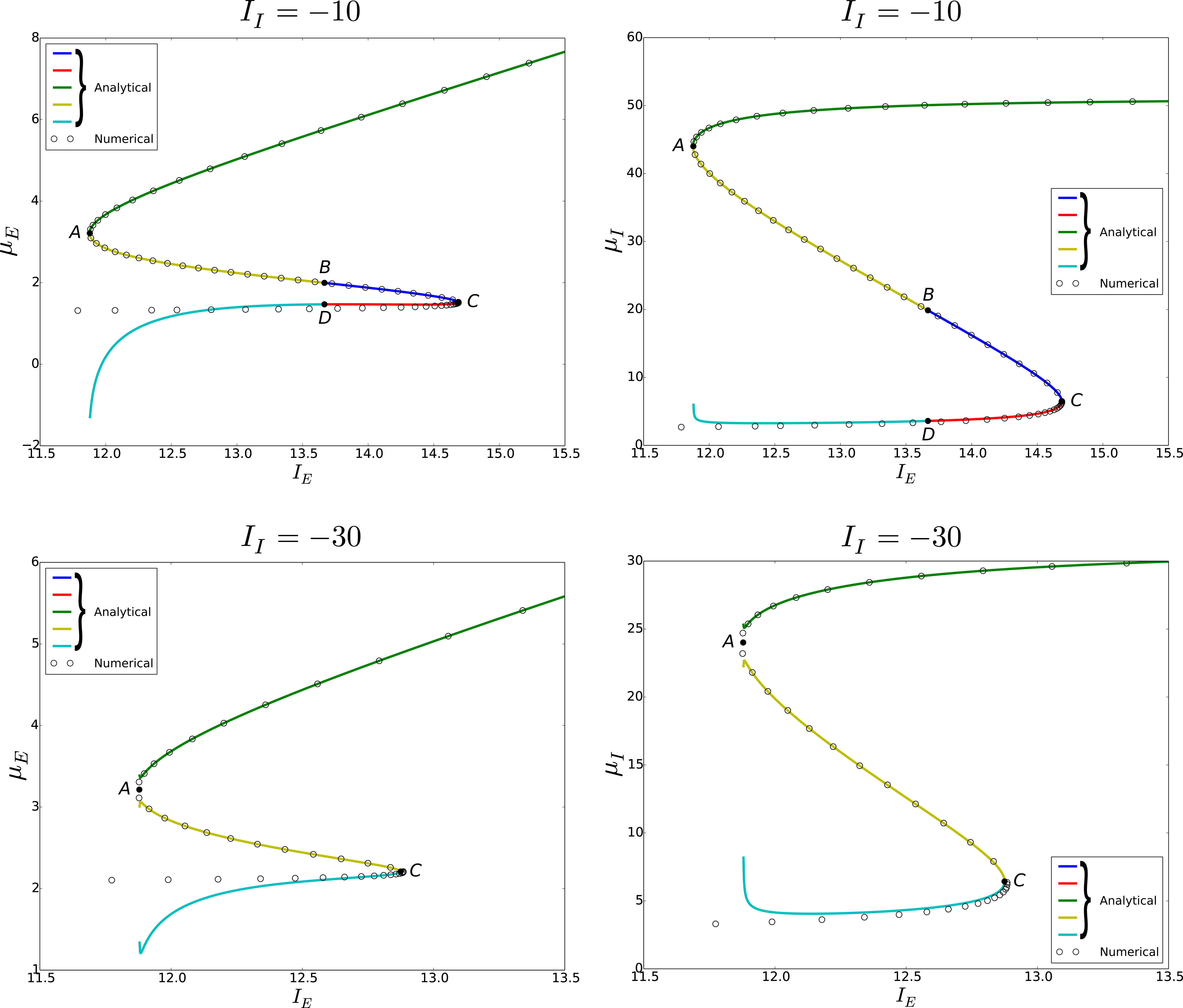

Supplement: S1 Fig — The panels on the top represent the solutions μE,I for II = −10, while those at the bottom are the solutions for II = −30. The figure shows a good agreement with the numerical solutions provided by Cl_MatCont on all the portions of the primary branch with the exception of most of the cyan colored curve, and also the green and yellow ones close to point A, where the first-order perturbative approximation does not work anymore due to the divergence of μE(1). For II = −10 the curves μE,I are made of 5 portions (green, yellow, blue, red, cyan), while for II = −30 the blue and red portions disappear, see text. (TIF) [file pcbi.1004992.s002.tif]

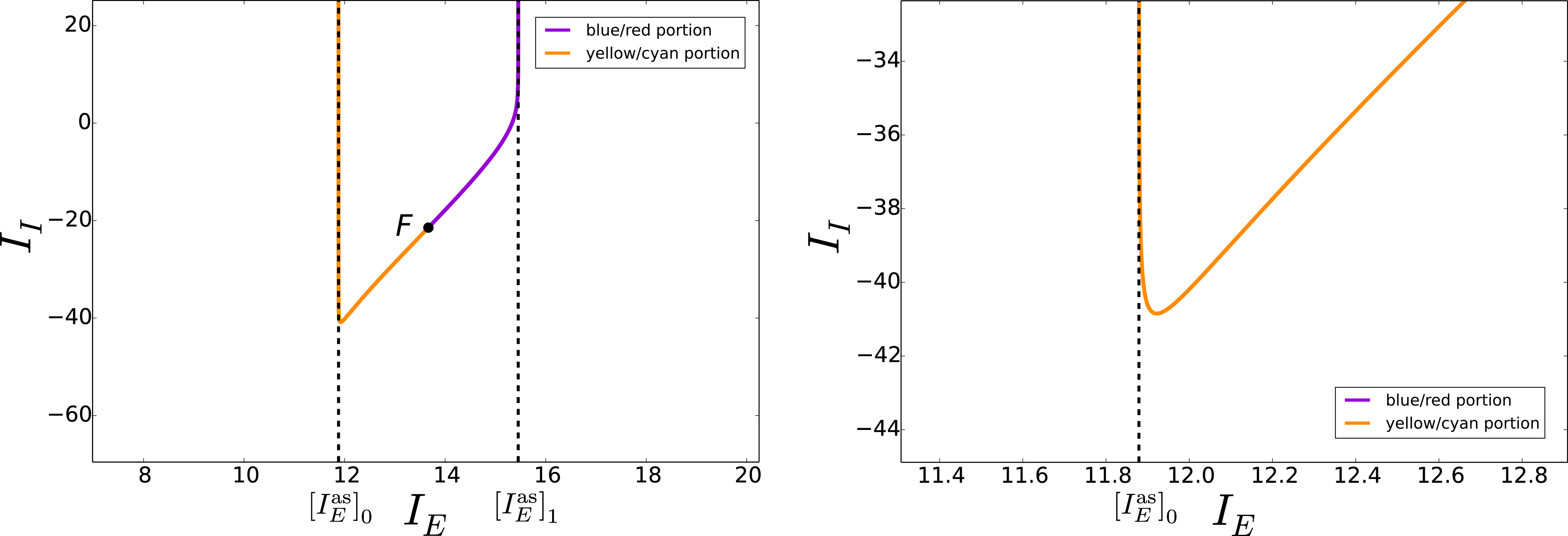

Supplement: S2 Fig — The zoom in the right-hand side of the figure shows that the approximation does not describe the cusp bifurcation (CP for short), even if the overall LP curve corresponds qualitatively to that shown in Fig 7 in the main text. (TIF) [file pcbi.1004992.s003.tif]

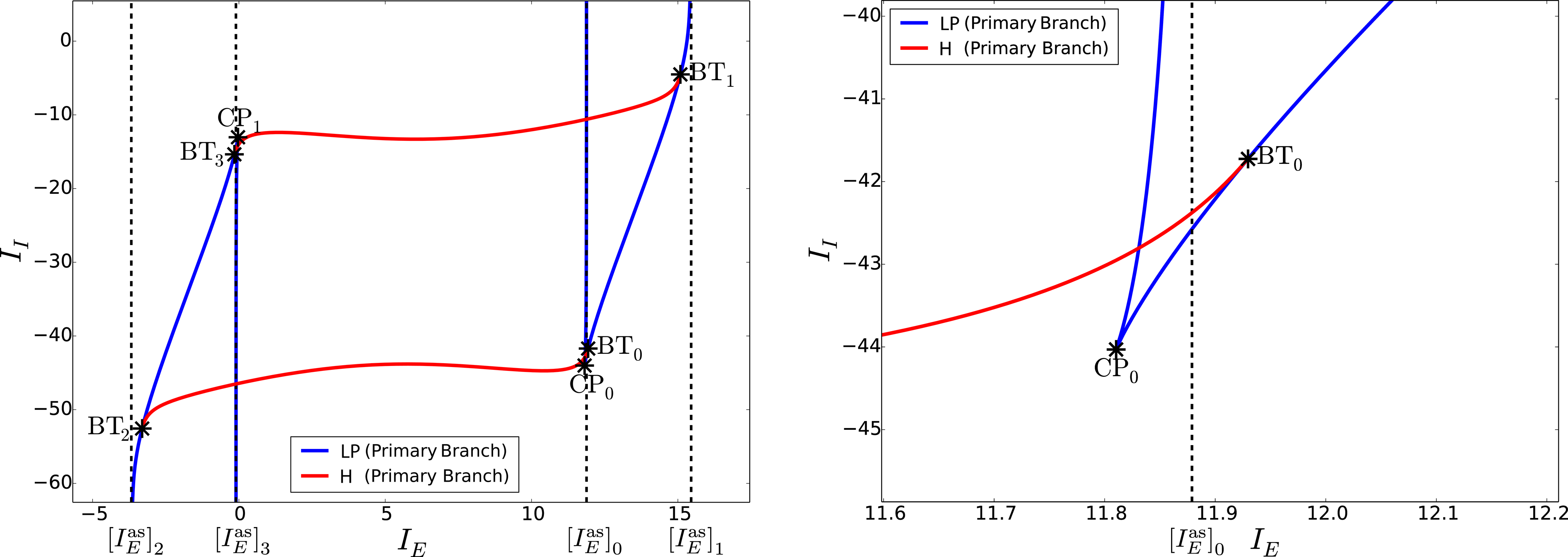

Supplement: S3 Fig — The zoom in the right-hand side of the figure shows the CP bifurcation, which is not predicted by the first-order perturbative expansion (compare with S2 Fig). (TIF) [file pcbi.1004992.s004.tif]

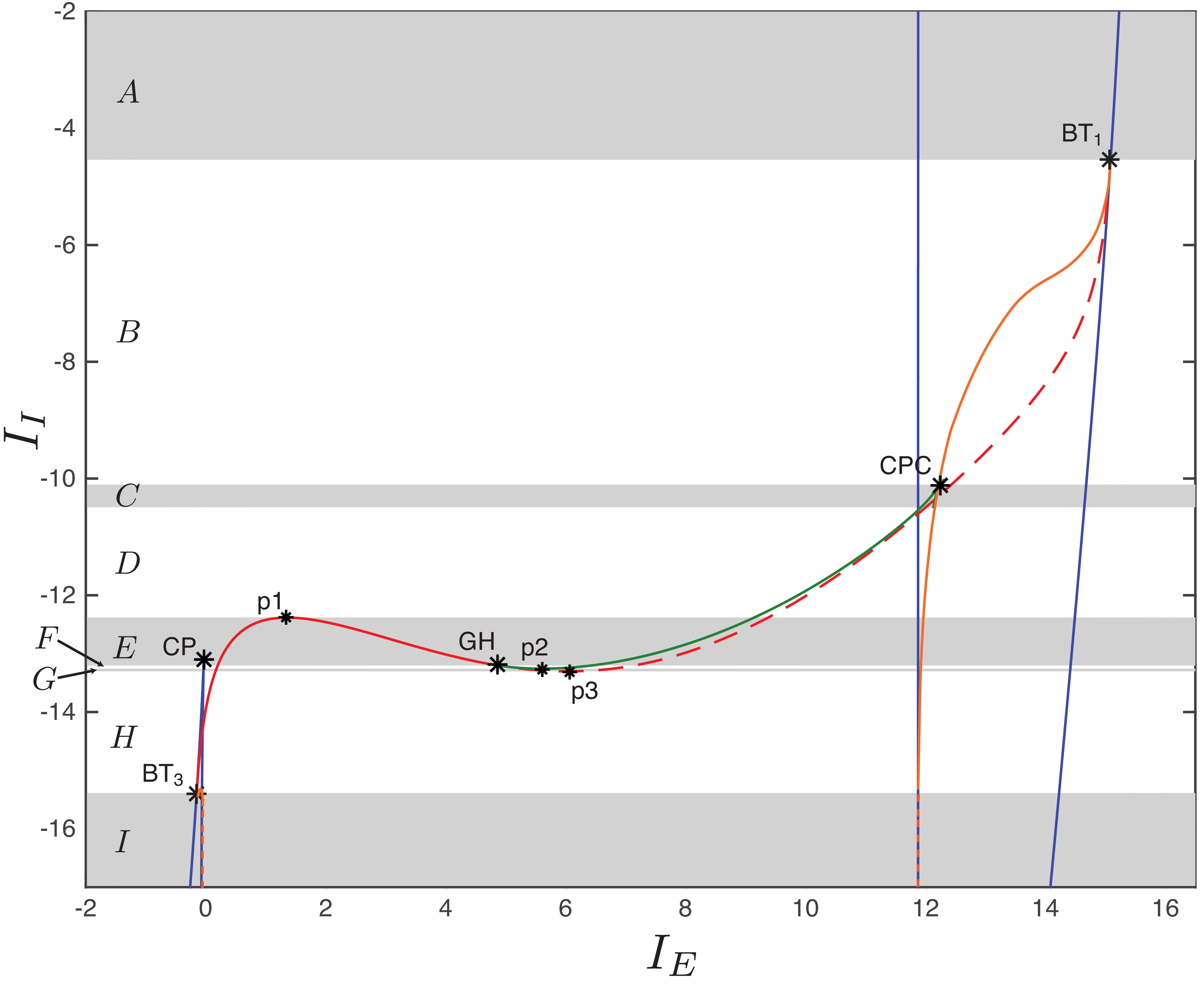

Supplement: S4 Fig — Due to the high symmetry of the codimension two bifurcation diagram shown in Fig 7 in the main text, we focus here on its upper-half part. In addition to the codimension two bifurcations presented in Fig 7, we consider three additional points p1, p2, p3 that allow us to divide the diagram horizontally in nine areas, identified by the letters A-I. The codimension one bifurcation diagram of each slice is shown in S5 Fig. For the meaning of the line colors see the main text. (TIF) [file pcbi.1004992.s005.tif]

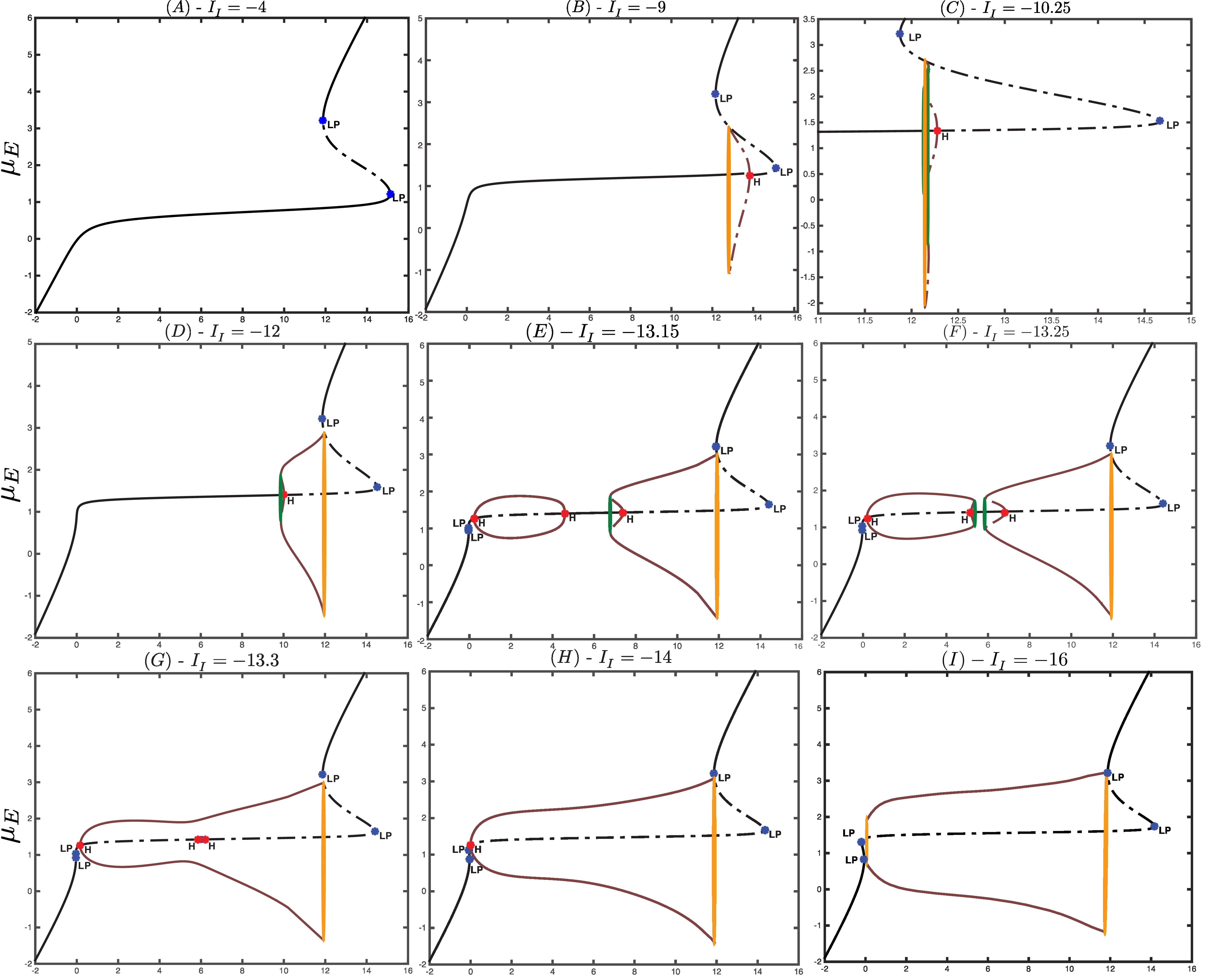

Supplement: S5 Fig — Each panel of this figure describes μE on the vertical axis as a function of IE on the horizontal axis (the panels of μI have been omitted, see text), for different values of II in areas A-I. The stable/unstable equilibrium curves are described by plain/dashed black lines. Saddle-node (LP), as well as Andronov-Hopf (H) bifurcations, lie on the equilibrium curve. Supercritical/subcritical H bifurcations give rise to stable/unstable limit cycles, whose maxima and minima are described by plain/dashed brown curves. Homoclines, which are characterized by large amplitude limit cycles with infinite period, are described by orange loops. The dark green loops identify the values of the current at which the limit cycles cross the limit point of cycles bifurcations. Here, limit cycles change stability. (TIF) [file pcbi.1004992.s006.tif]

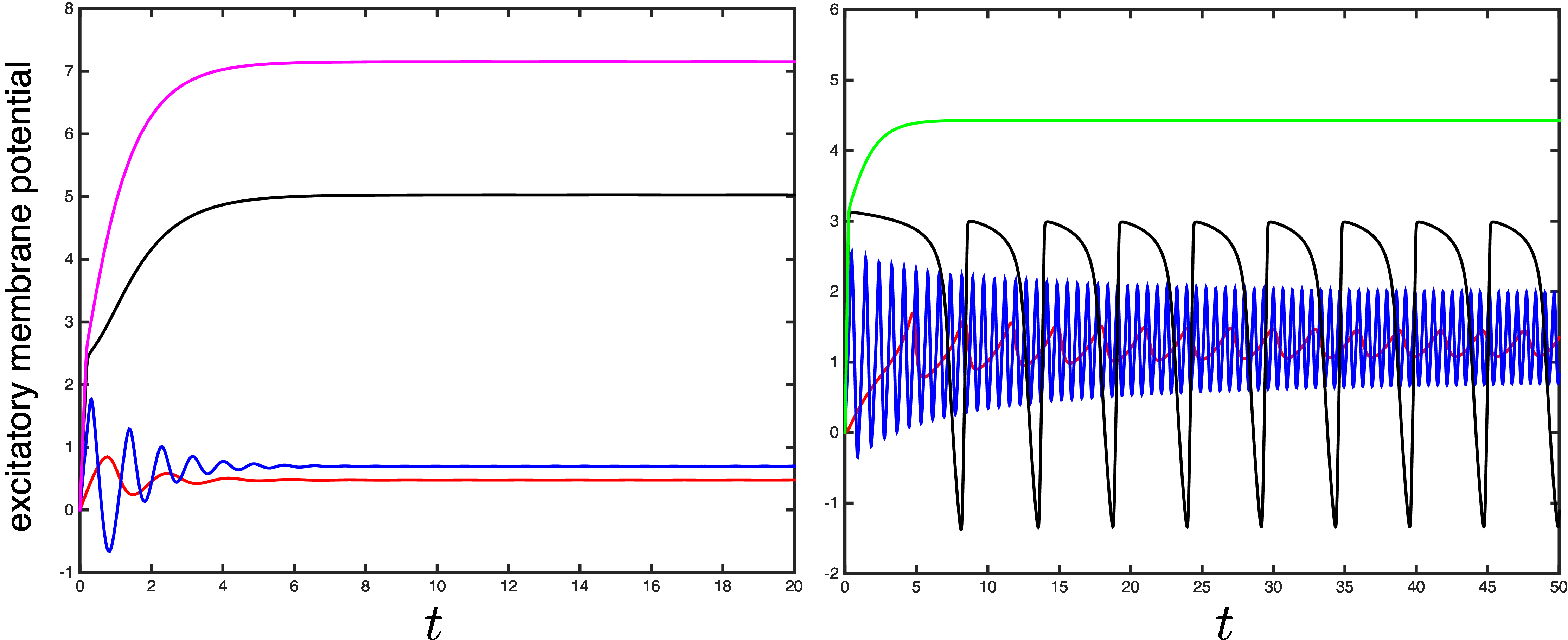

Supplement: S6 Fig — This figure shows the time evolution of the excitatory membrane potentials Vi(t) (for any i = 0,…, NE−1), obtained from Eq (3) of the main text for different values of the external currents. Left, we fix II = −4 (area A in S5 Fig). For IE = 2 and IE = 7 the solutions converge to stable foci, giving rise to damped oscillations (red and blue curve, respectively). For IE = 13 and IE = 15 the solutions converge to stable nodes (black and purple curve, respectively). Right, we fix II = −13.3 (area G in S5 Fig). For IE = 0.216 we find low-amplitude oscillations of about 35 Hz (red curve). For IE = 5.564 the amplitude of the oscillations is larger than the previous case, and the frequency increases up to 160 Hz (blue curve). High-amplitude oscillations occur for IE = 11.85; since this current is close to the homoclinic bifurcation, the frequency decays to 19 Hz (black curve). Finally, for IE = 12.5 the system reaches a stable node (green curve). (TIF) [file pcbi.1004992.s007.tif]

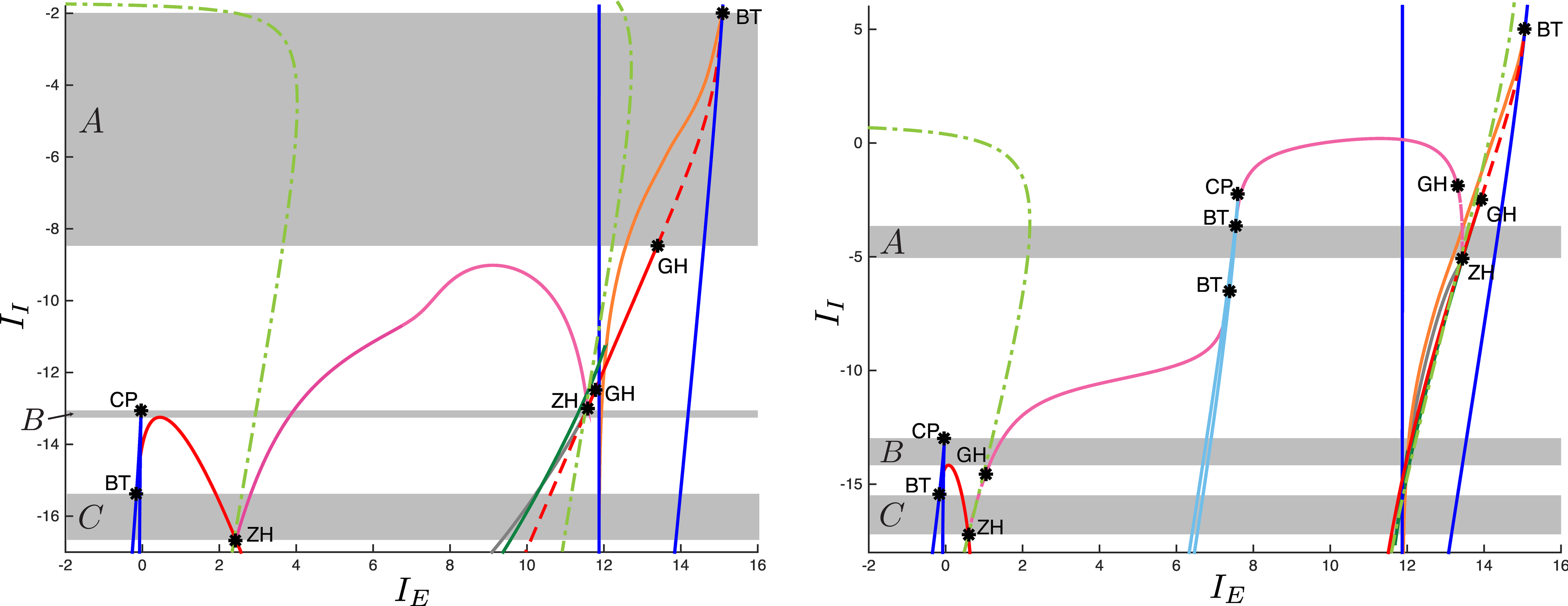

Supplement: S7 Fig — As in S4 Fig, here we focus on the upper-half part of the codimension two bifurcation diagrams obtained for JII = −34 (left), and JII = −100 (right). Specifically, we identify three regions in both of them, represented by gray backgrounds, whose corresponding codimension one diagrams are shown in S8 and S9 Figs. (TIF) [file pcbi.1004992.s008.tif]

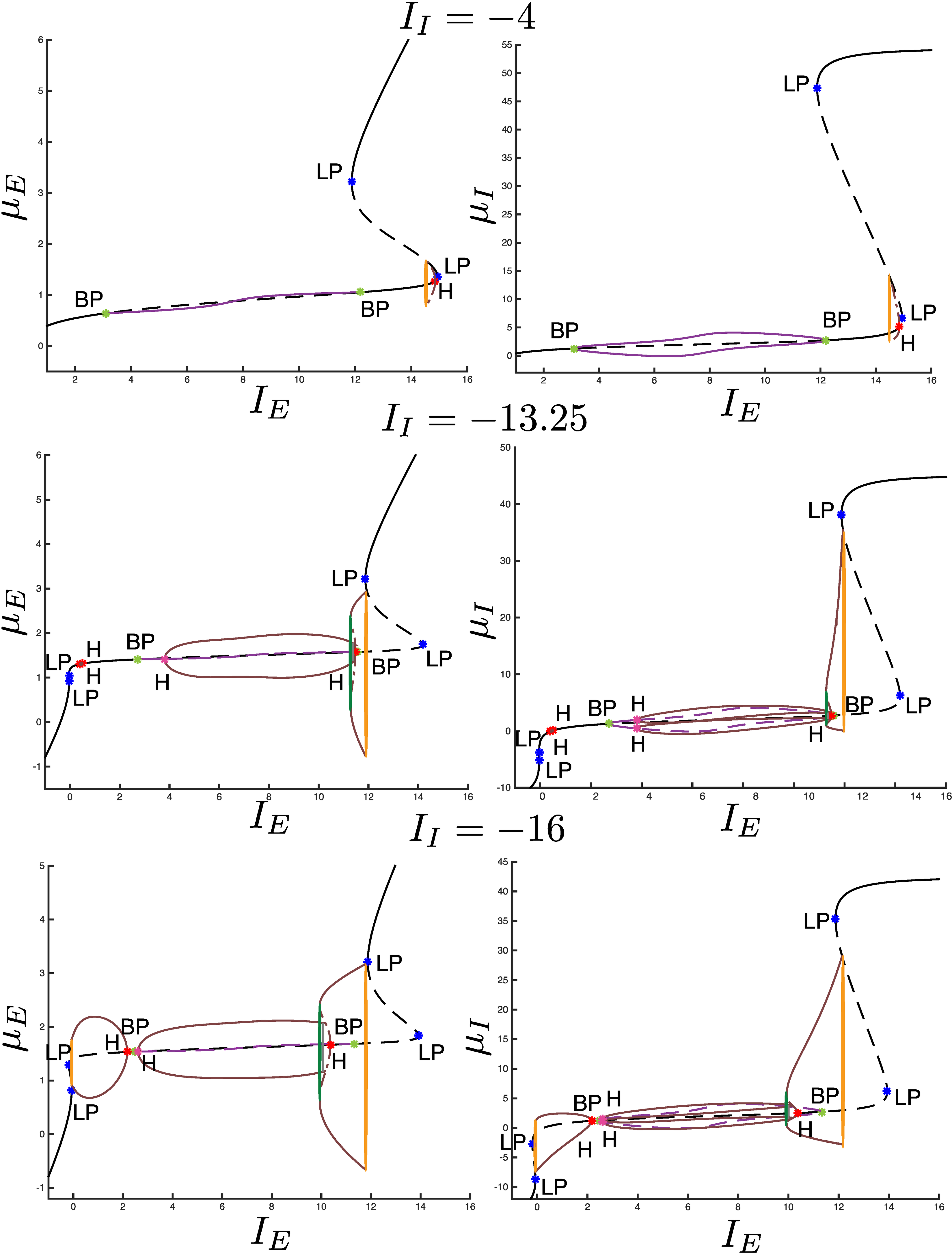

Supplement: S8 Fig — Each row of this figure describes μE and μI for JII = −34 as a function of IE, for three different values of II. (TIF) [file pcbi.1004992.s009.tif]

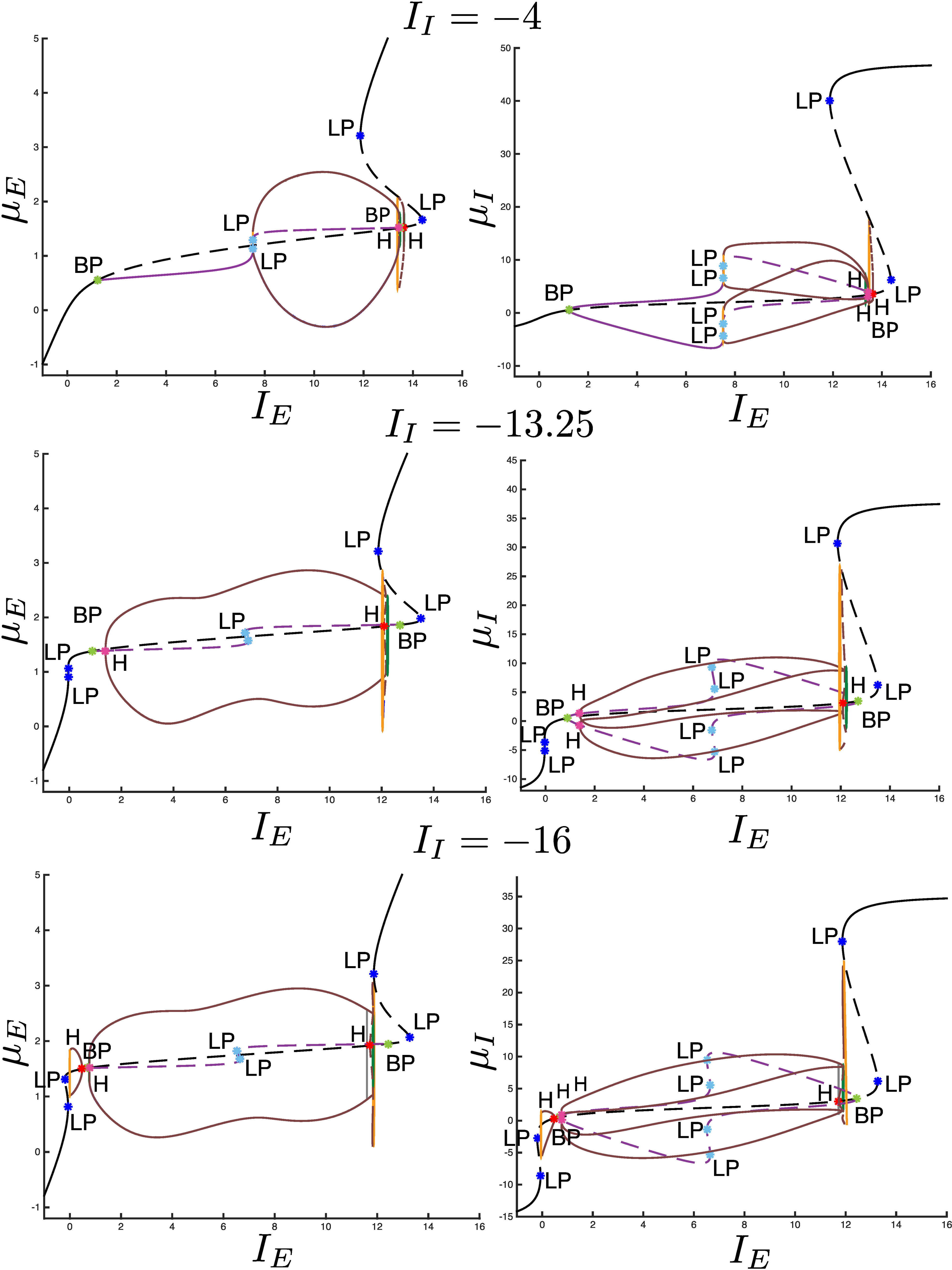

Supplement: S9 Fig — As in S8 Fig, but for stronger inhibition. (TIF) [file pcbi.1004992.s010.tif]

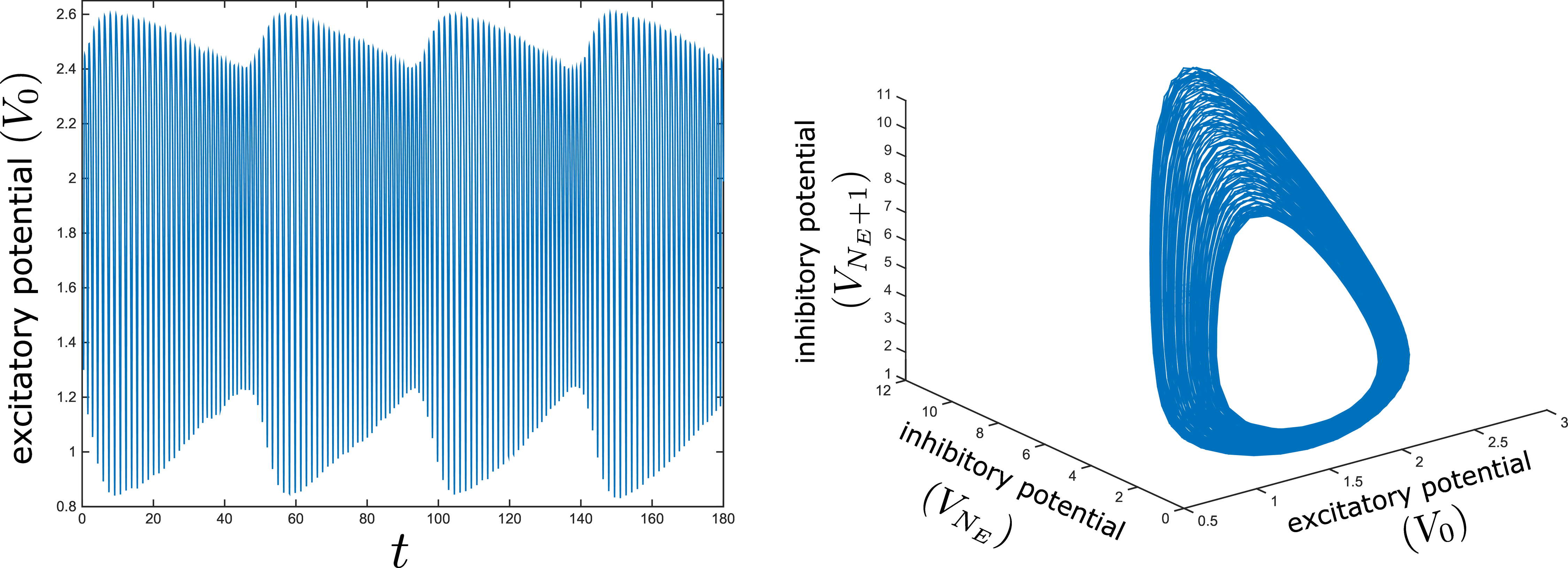

Supplement: S10 Fig — This figure shows the time evolution of the excitatory membrane potentials at the torus bifurcation (left) and the corresponding trajectory in the phase space (right). Both the panels have been obtained for JII = −100, II = −16 (area C in S7 Fig) and IE ≈ 11.804. From the left panel it is easy to see that the time evolution on the torus is characterized by two (incommensurable) frequencies. (TIF) [file pcbi.1004992.s011.tif]

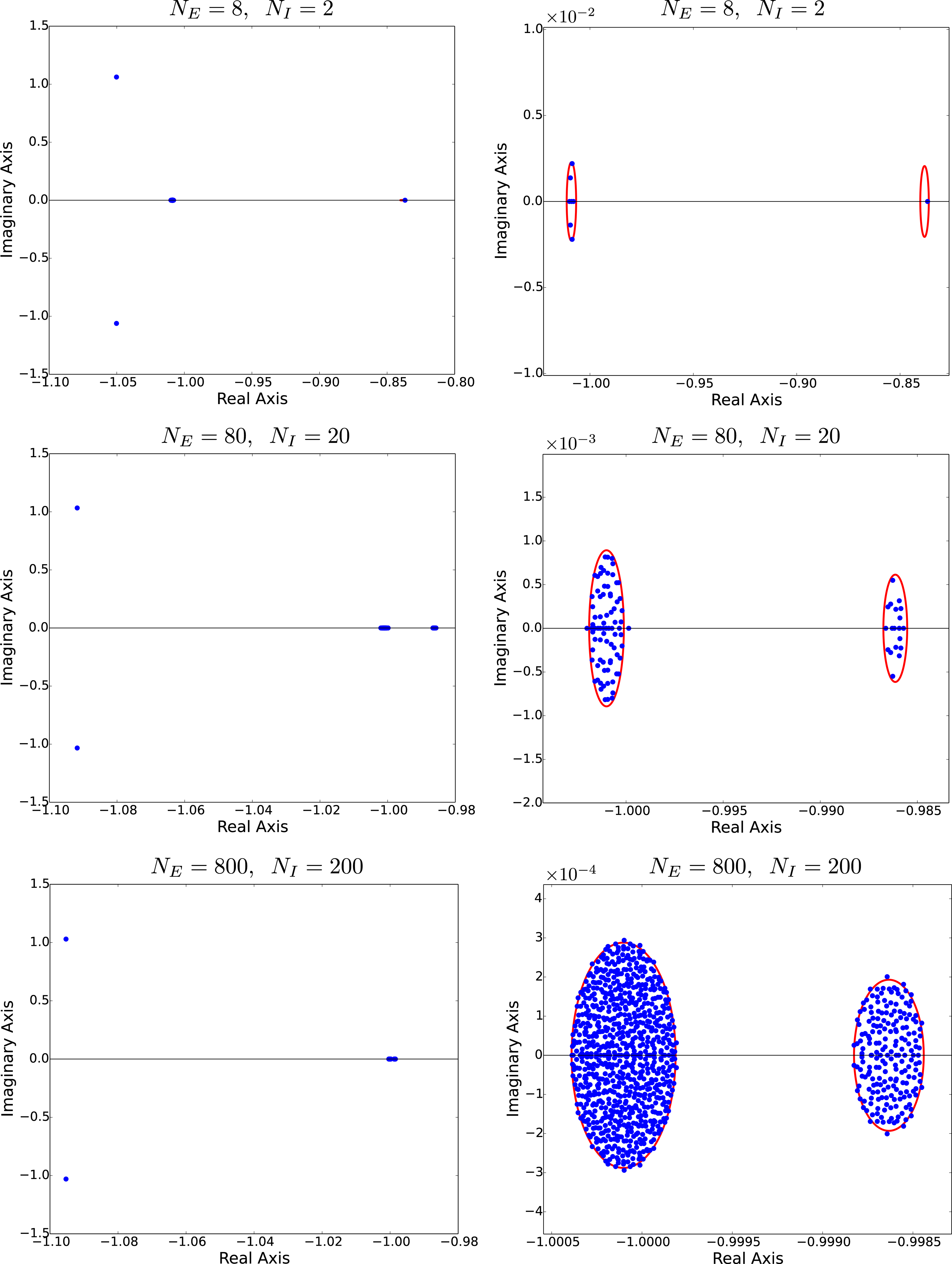

Supplement: S11 Fig — The panels on the left column show the whole set of eigenvalues (blue dots) of the Jacobian matrix for increasing network’s size (N = 10, 100 and 1,000), while the panels on the right column represent a zoom of the eigenvalues close to the x-axis in the complex plane. The eigenvalues have been calculated numerically for J¯EE=10, J¯EI=-70, J¯IE=70, J¯II=-10, σJEE = σJIE = 1, σJEI = σJII = 0.1, and IE = II = 0, while the remaining parameters have been chosen according to Table 1 in the main text. The panels on the right show that the eigenvalues λE and λI split into NE−1 and NI−1 eigenvalues respectively, which are distributed according to a circular law. The red circles represent the theoretical prediction of the support of the distribution according to random matrix theory, see text. (TIF) [file pcbi.1004992.s012.tif]

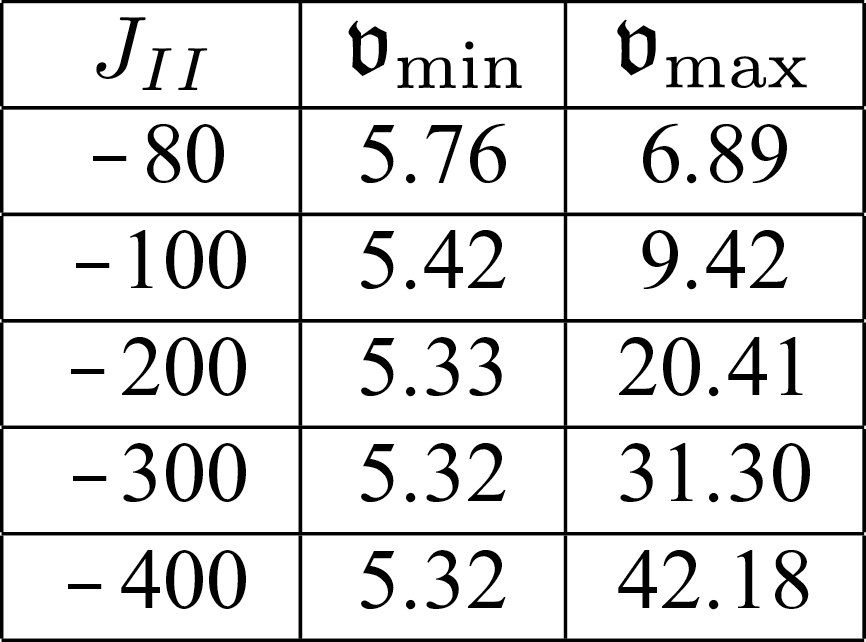

Supplement: S1 Table — This table reports the range of the parameter v=defμI,0 obtained numerically from the system of inequalities (S57) in S1 Text, for different values of JII. We observe that for large ∣JII∣ the parameter vmin reaches a constant value, while vmax increases linearly. This result suggests that simple asymptotic expressions of vmin and vmax can be derived analytically. Nevertheless this calculation is beyond the purpose of the article, and is left to the interested reader. (TIF) [file pcbi.1004992.s013.tif]
